# Supplementary material for: Estimating sleep parameters using an accelerometer without sleep diary
Source: Sci Rep. 2018 Aug 28;8:12975. doi: 10.1038/s41598-018-31266-z (PMC6113241; doi:10.1038/s41598-018-31266-z)
Supplement: Supplementary file 1 — Supplementary Information [file 41598_2018_31266_MOESM1_ESM.pdf]

Supplementary information

Manuscript title: Estimating sleep parameters using an accelerometer without sleep diary

Authors: V.T. van Hees[1*], S. Sabia[2,3], S.E. Jones[4], A.R. Wood[4], K.N. Anderson[5], M. Kivimäki[3], T.M. Frayling[4], A. I. Pack[6], M Bucan[7,8], M.I. Trenell[9], Diego R. Mazzotti[6], P. R. Gehrman[6,8], B. A. Singh-Manoux[2,3], M. N. Weedon[4]

1. Netherlands eScience Center, Amsterdam, Netherlands
2. INSERM U1018, Centre for Research in Epidemiology and Population Health, Université Paris-Saclay, France
3. Department of Epidemiology & Public Health, University College London (UCL), London, United Kingdom
4. University of Exeter Medical School, Genetics of Complex Traits, Exeter
5. Regional Sleep Service, Freeman Hospital, Newcastle
6. Center for Sleep and Circadian Neurobiology, Perelman School of Medicine, University of Pennsylvania, Philadelphia, USA
7. Department of Genetics, Perelman School of Medicine, University of Pennsylvania School of Medicine, Philadelphia, Pennsylvania, United States of America
8. Department of Psychiatry, Perelman School of Medicine, University of Pennsylvania School of Medicine, Philadelphia, Pennsylvania, United States of America
9. Movelab, Newcastle University, Newcastle, UK

*Corresponding author: V.T. van Hees, v.vanhees@esciencecenter.nl, Netherlands eScience Center, Science Park 140, 1098 XG Amsterdam, The Netherlands

1 PSG sleep clinic patients (Newcastle upon tyne) with accelerometer classifcation

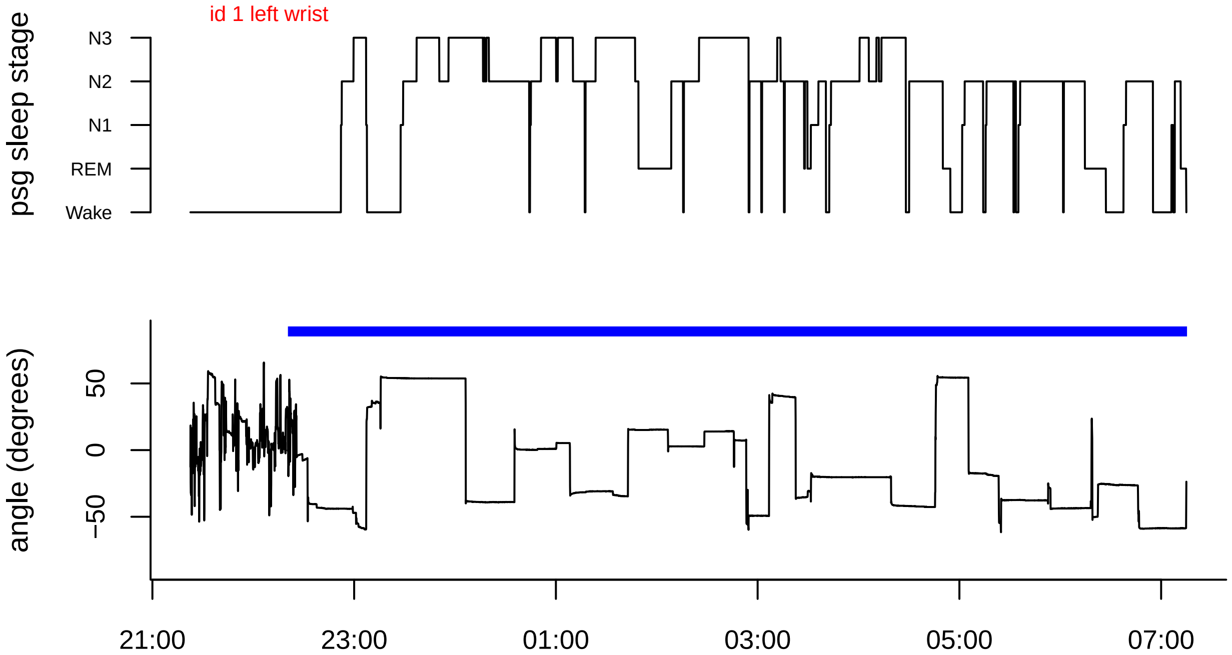

id 1 left wrist
psg sleep stage
N3
N2
N1
REM
Wake
angle (degrees)
50
0
−50
21:00
23:00
01:00
03:00
05:00
07:00

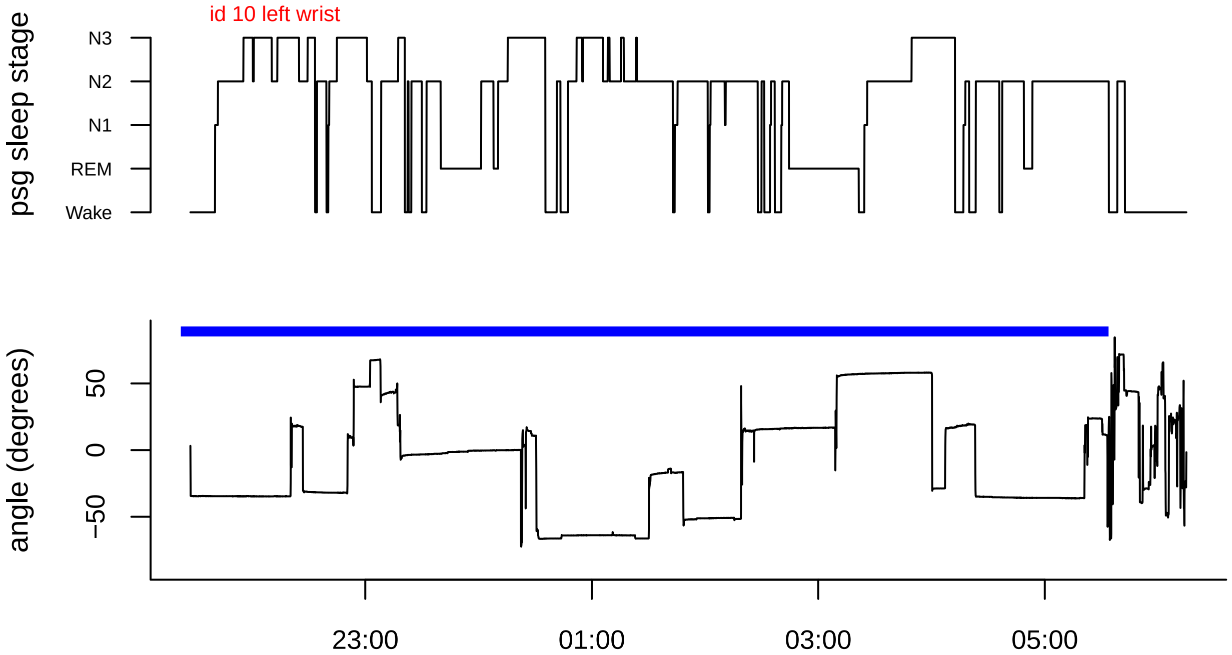

id 10 left wrist
psg sleep stage
N3
N2
N1
REM
Wake
angle (degrees)
50
0
−50
23:00
01:00
03:00
05:00

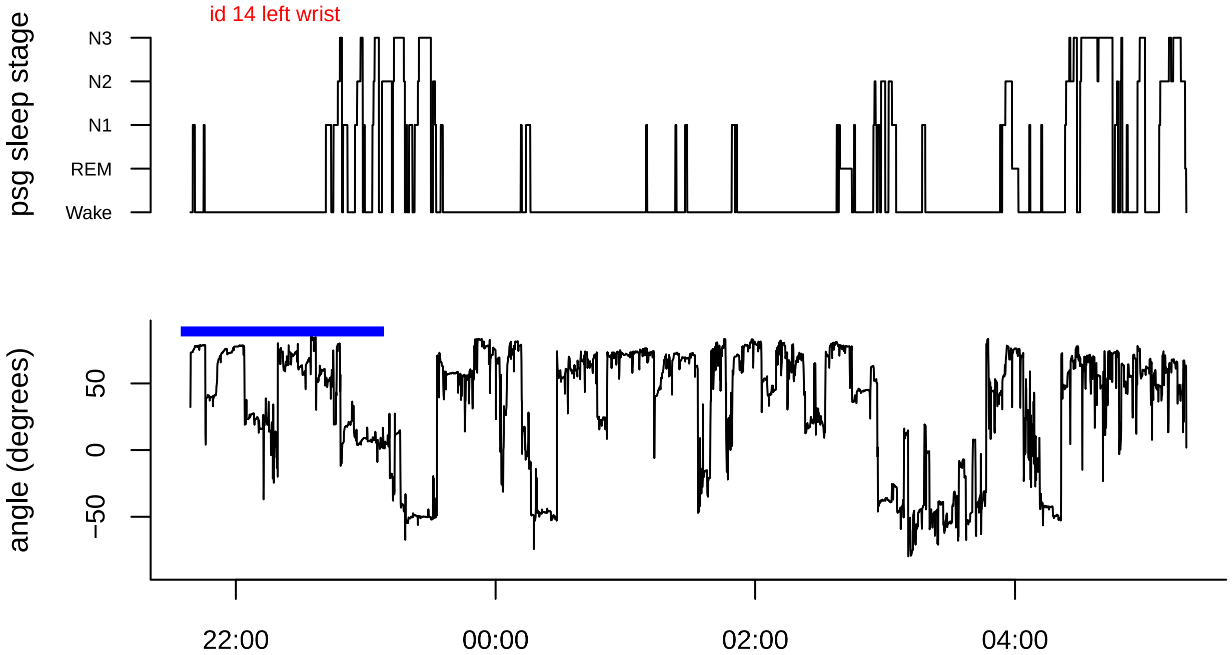

id 14 left wrist
psg sleep stage
N3
N2
N1
REM
Wake
angle (degrees)
50
0
−50
22:00
00:00
02:00
04:00

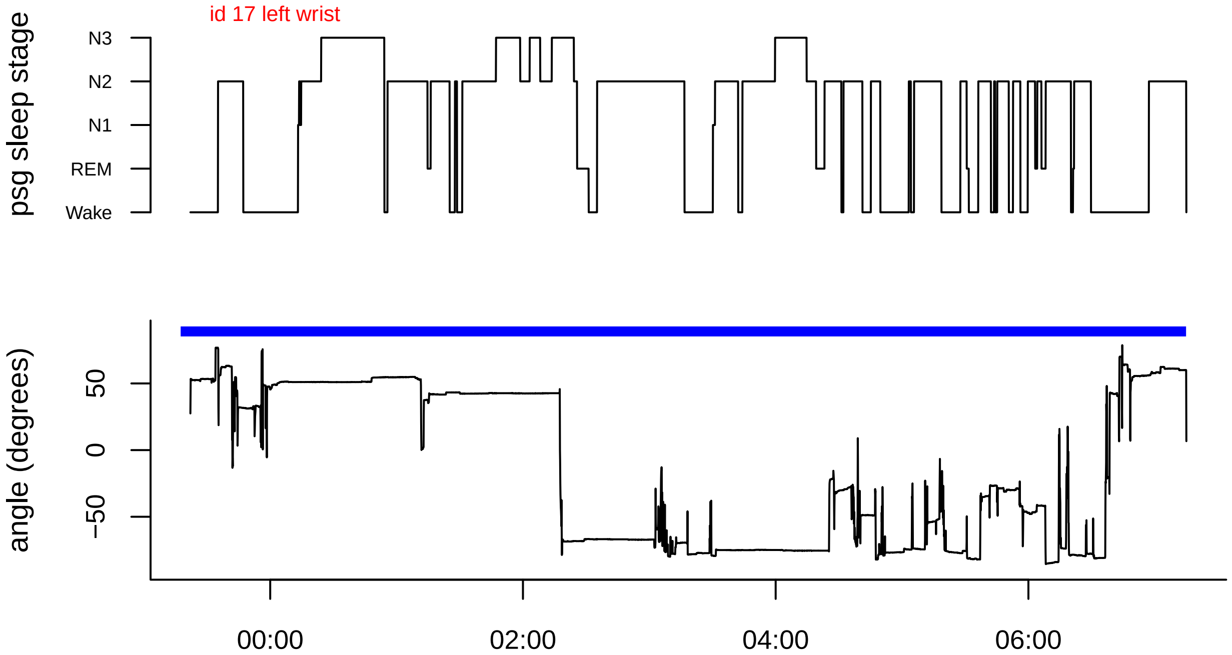

id 17 left wrist
psg sleep stage
N3
N2
N1
REM
Wake
angle (degrees)
50
0
−50
00:00
02:00
04:00
06:00

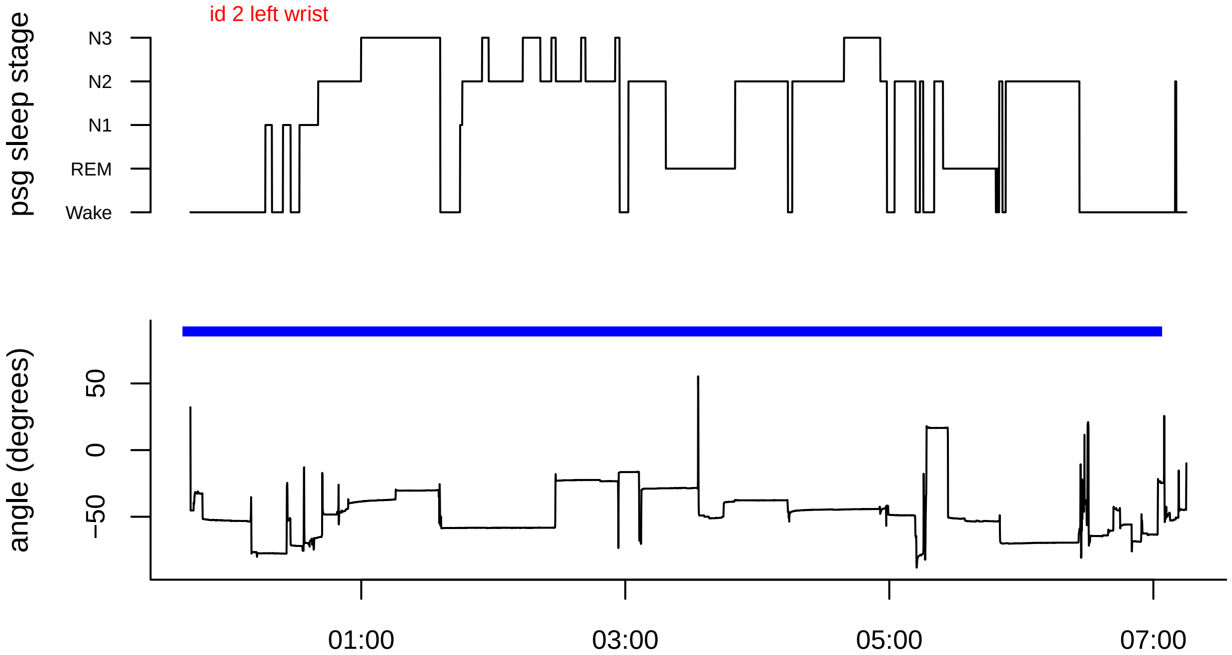

id 2 left wrist
psg sleep stage
N3
N2
N1
REM
Wake
angle (degrees)
50
0
−50
01:00
03:00
05:00
07:00

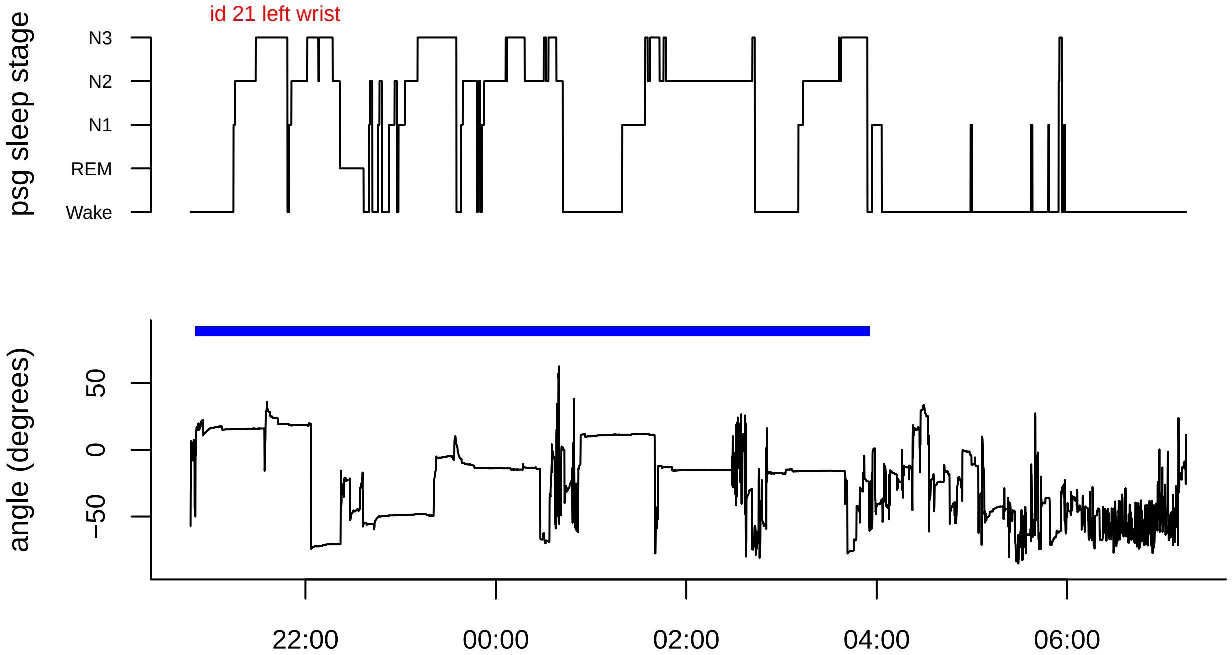
id 21 left wrist
psg sleep stage
N3
N2
N1
REM
Wake
angle (degrees)
50
0
−50
22:00
00:00
02:00
04:00
06:00

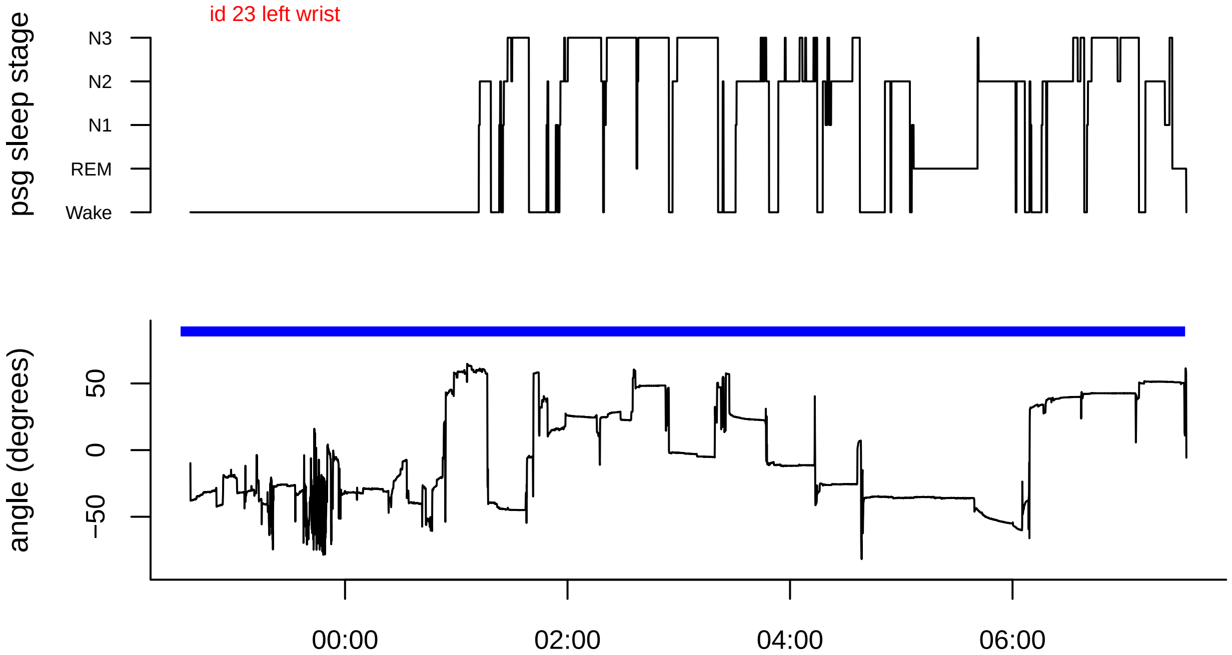

id 23 left wrist
psg sleep stage
N3
N2
N1
REM
Wake
angle (degrees)
50
0
−50
00:00
02:00
04:00
06:00

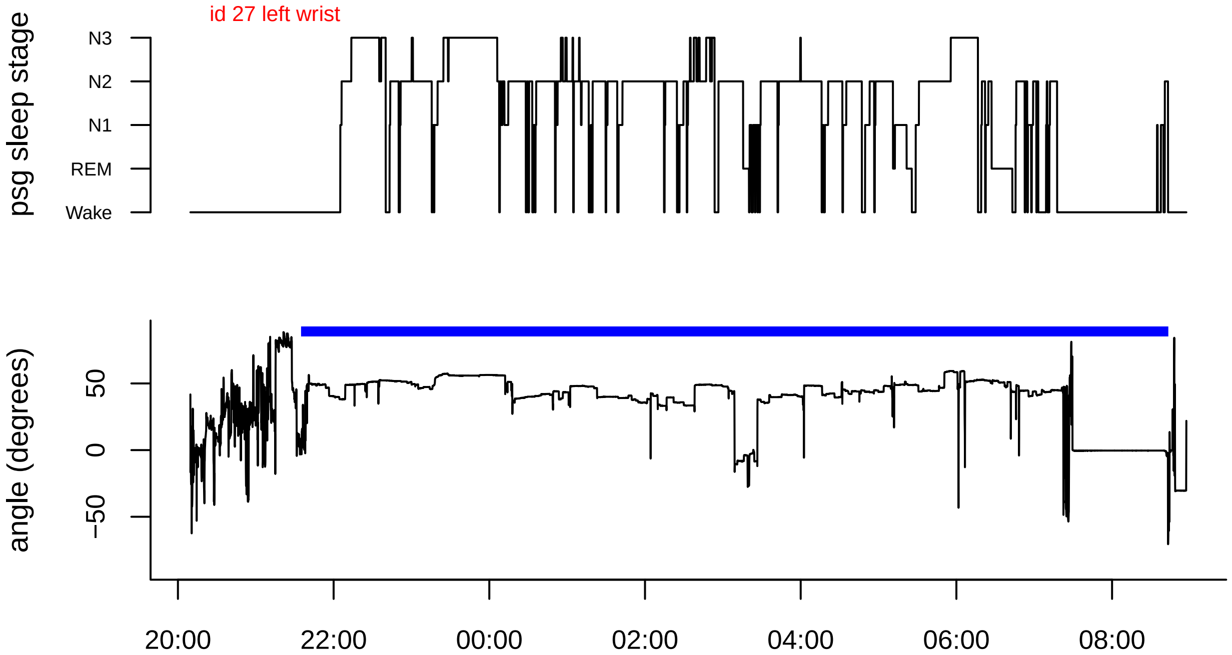
id 27 left wrist
psg sleep stage
N3
N2
N1
REM
Wake
angle (degrees)
50
0
−50
20:00
22:00
00:00
02:00
04:00
06:00
08:00

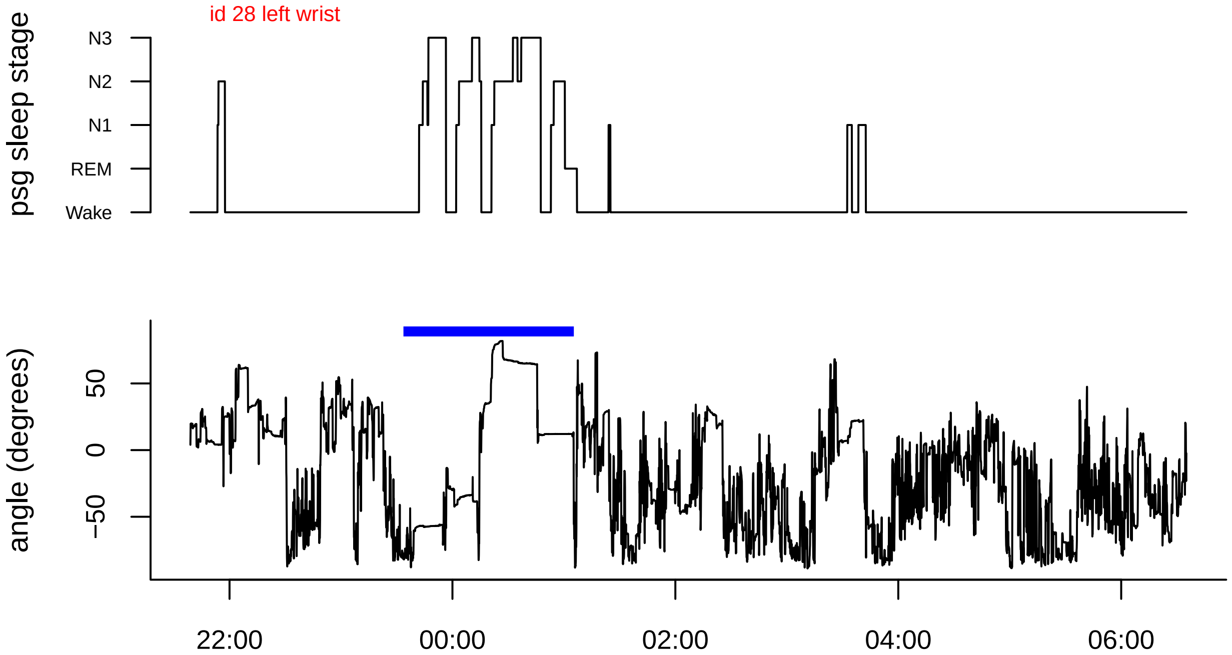

id 28 left wrist
psg sleep stage
N3
N2
N1
REM
Wake
angle (degrees)
50
0
−50
22:00
00:00
02:00
04:00
06:00

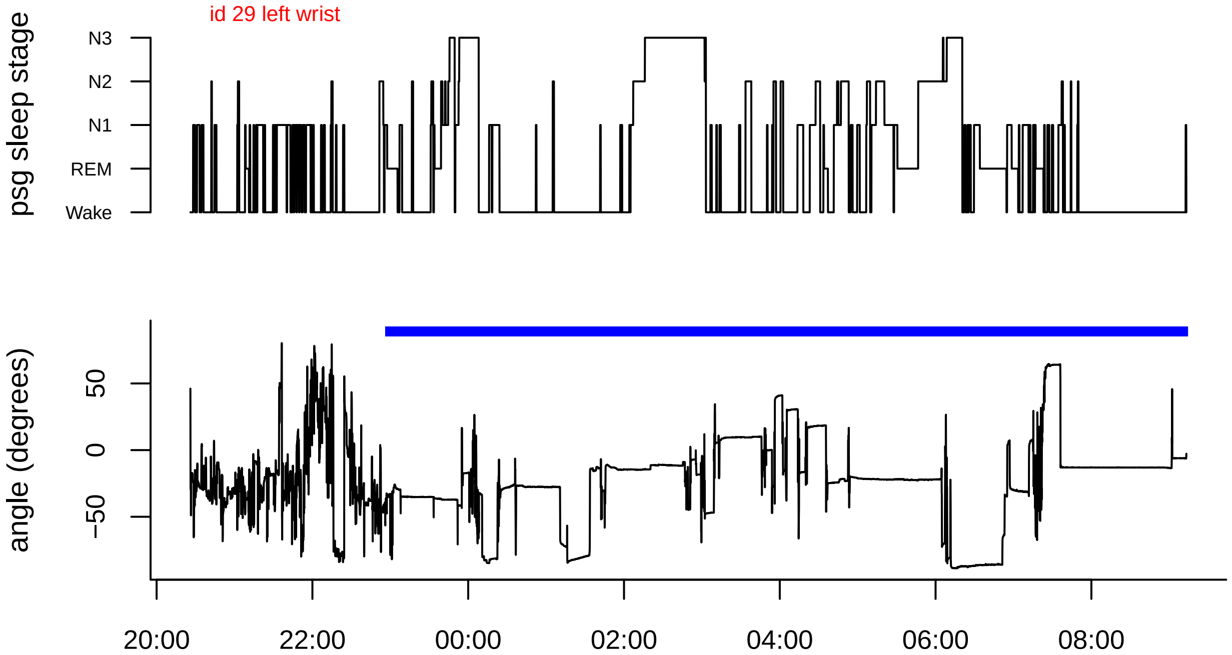
id 29 left wrist
psg sleep stage
N3
N2
N1
REM
Wake
angle (degrees)
50
0
−50
20:00
22:00
00:00
02:00
04:00
06:00
08:00

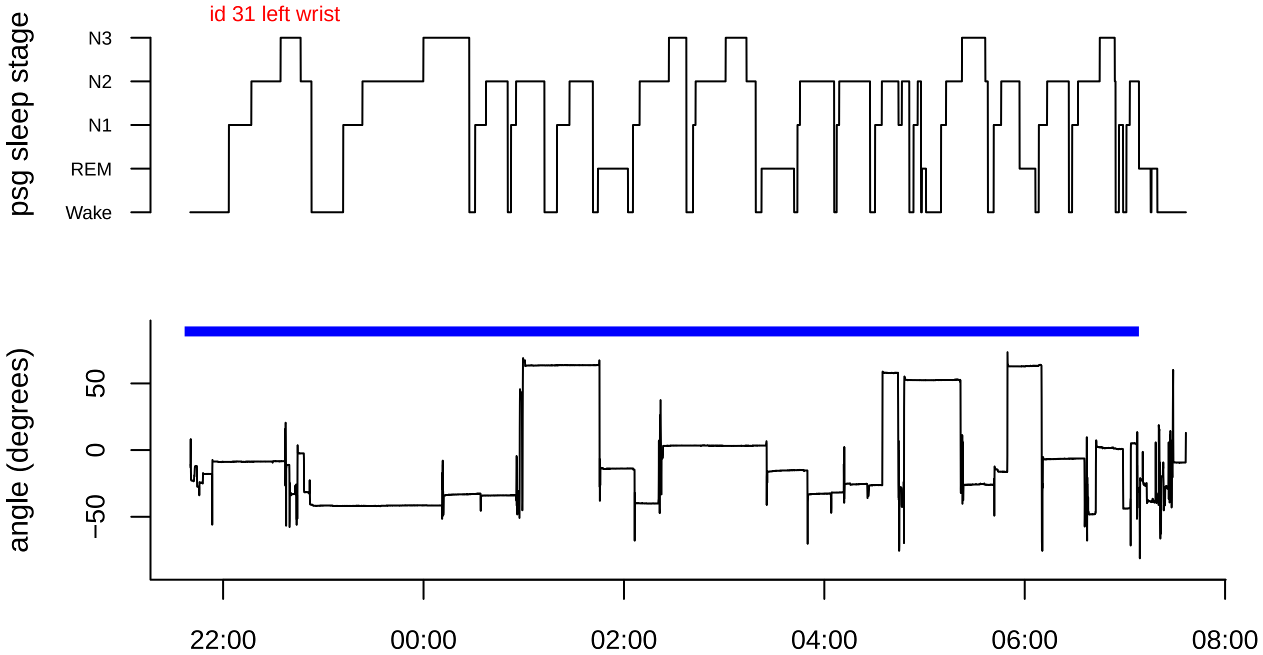

id 31 left wrist
psg sleep stage
N3
N2
N1
REM
Wake
angle (degrees)
50
0
−50
22:00
00:00
02:00
04:00
06:00
08:00

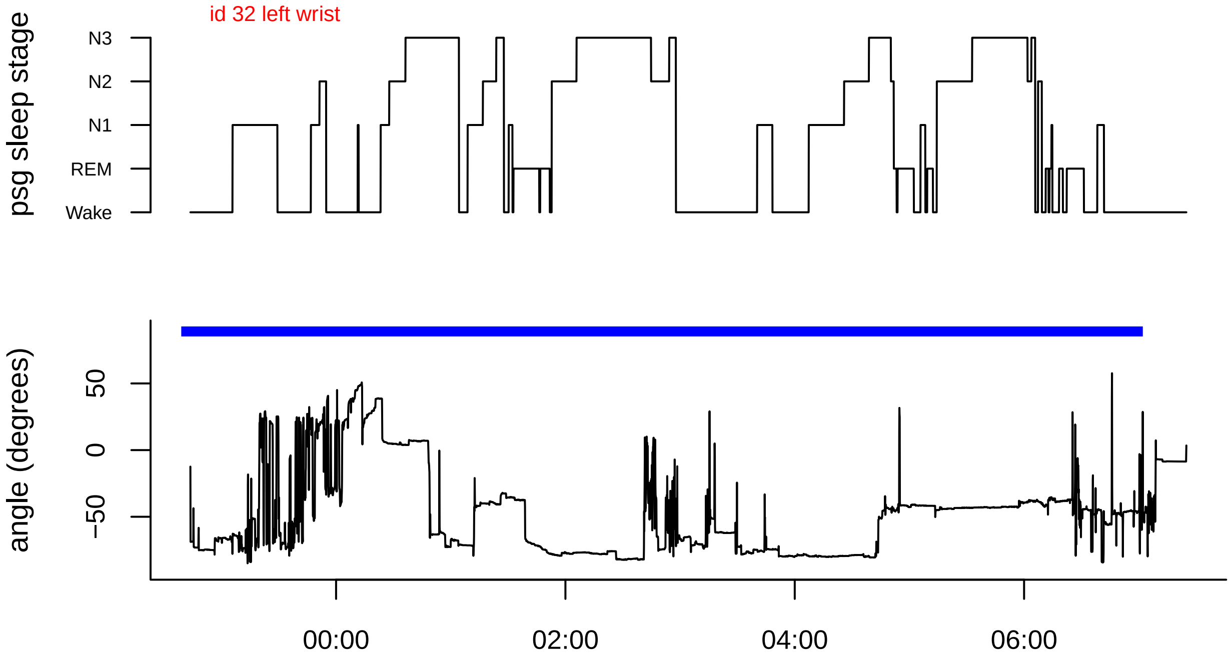

id 32 left wrist
psg sleep stage
N3
N2
N1
REM
Wake
angle (degrees)
50
0
−50
00:00
02:00
04:00
06:00

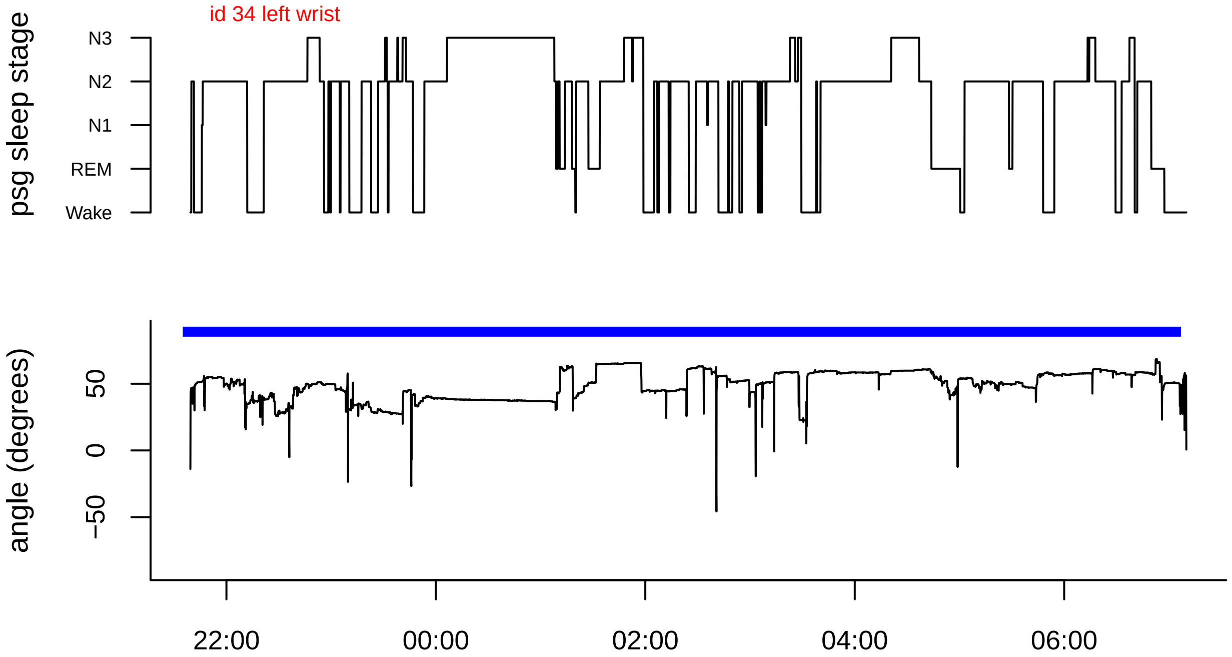

id 34 left wrist
psg sleep stage
N3
N2
N1
REM
Wake
angle (degrees)
50
0
−50
22:00
00:00
02:00
04:00
06:00

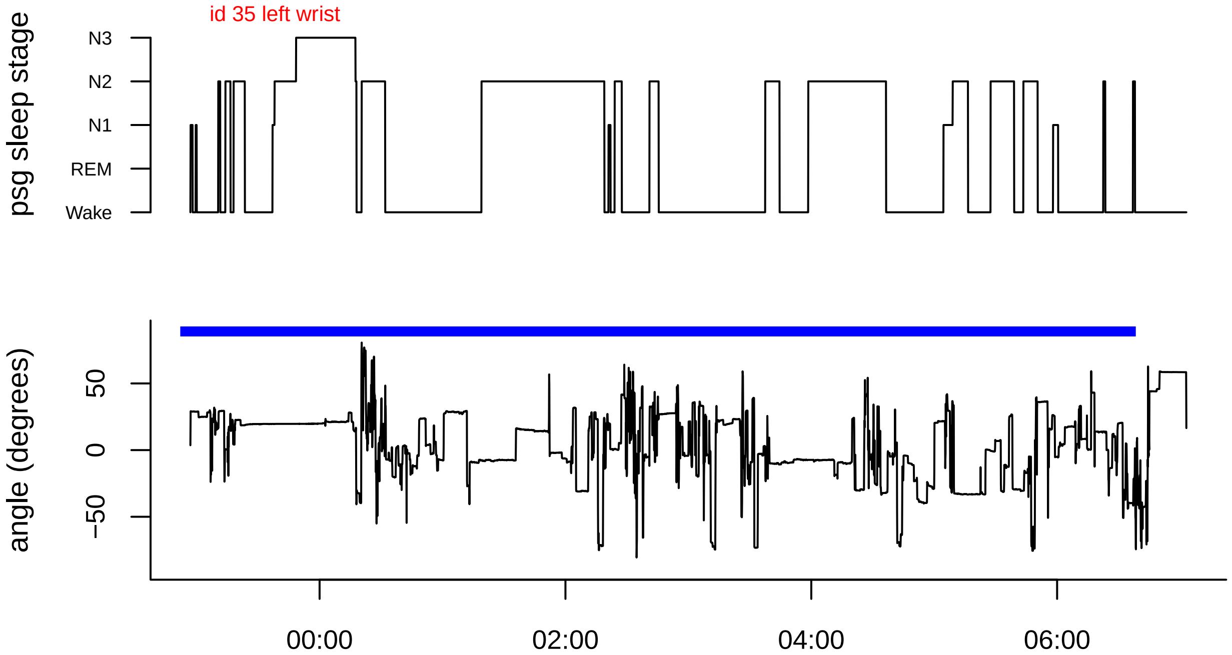
id 35 left wrist
psg sleep stage
N3
N2
N1
REM
Wake
angle (degrees)
50
0
−50
00:00
02:00
04:00
06:00

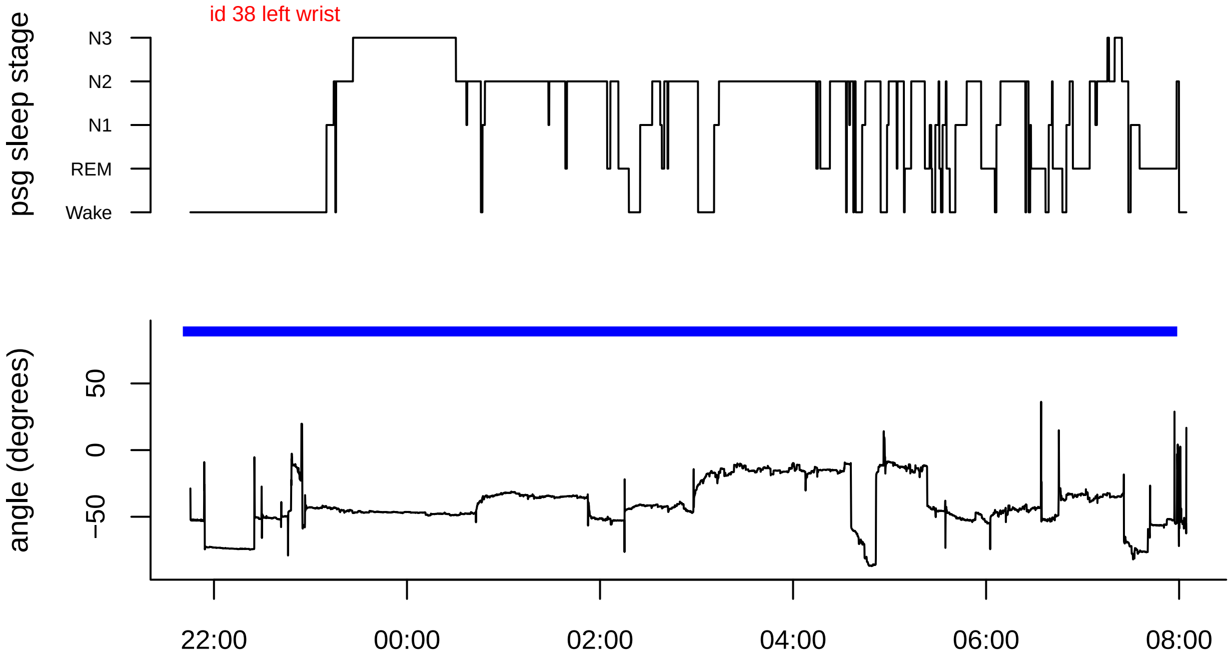

id 38 left wrist
psg sleep stage
N3
N2
N1
REM
Wake
angle (degrees)
50
0
−50
22:00
00:00
02:00
04:00
06:00
08:00

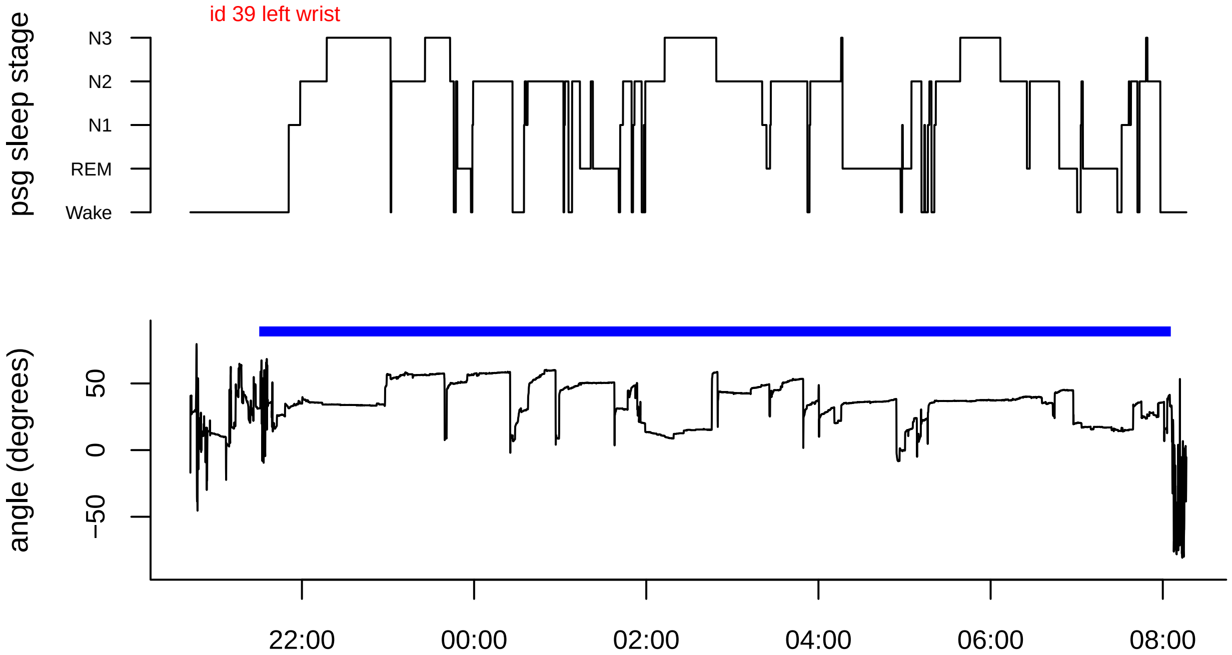
id 39 left wrist
psg sleep stage
N3
N2
N1
REM
Wake
angle (degrees)
50
0
−50
22:00
00:00
02:00
04:00
06:00
08:00

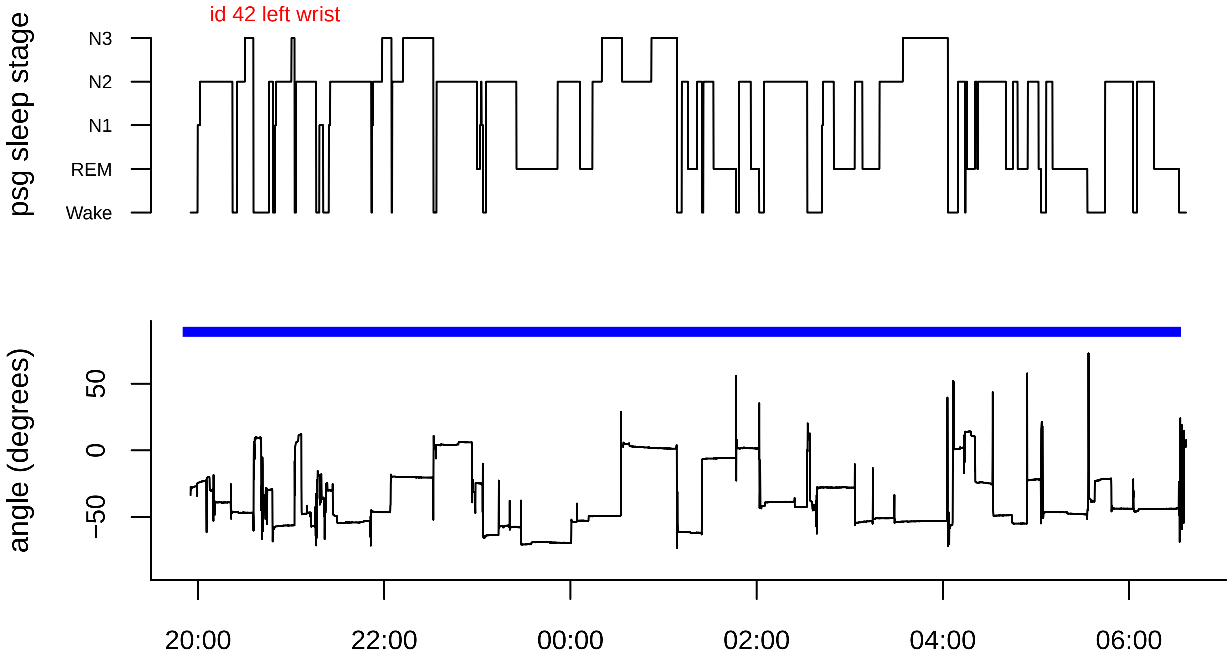
id 42 left wrist
psg sleep stage
N3
N2
N1
REM
Wake
angle (degrees)
50
0
−50
20:00
22:00
00:00
02:00
04:00
06:00

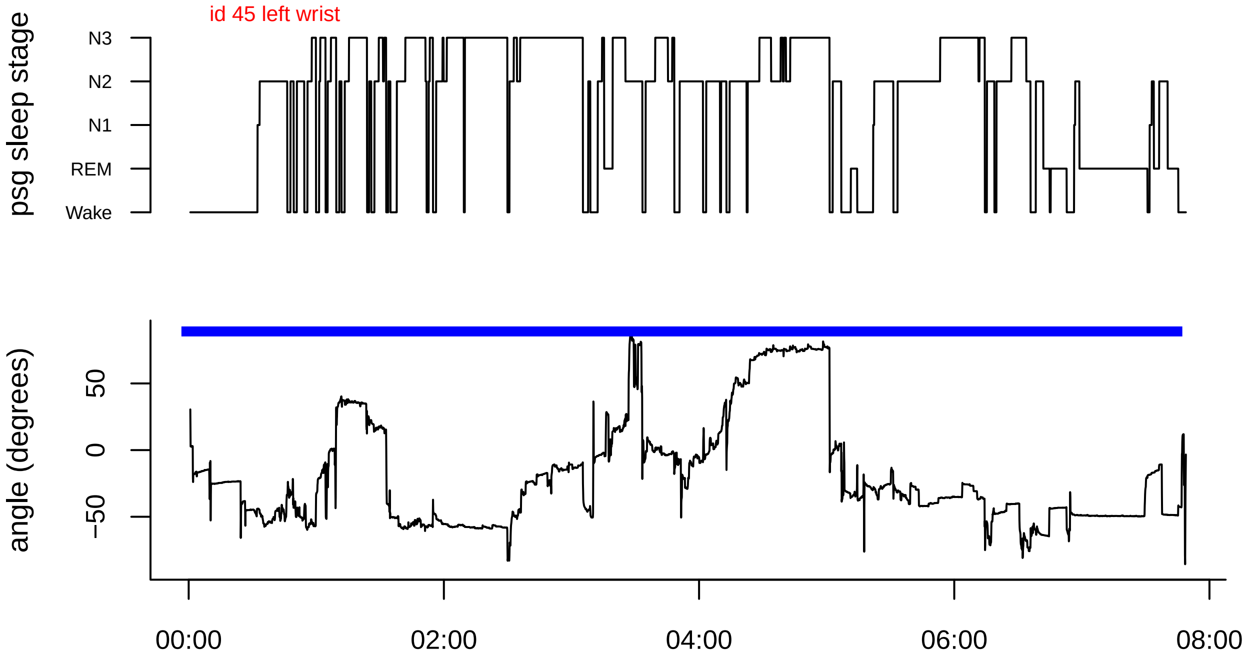

id 45 left wrist
psg sleep stage
N3
N2
N1
REM
Wake
angle (degrees)
50
0
−50
00:00
02:00
04:00
06:00
08:00

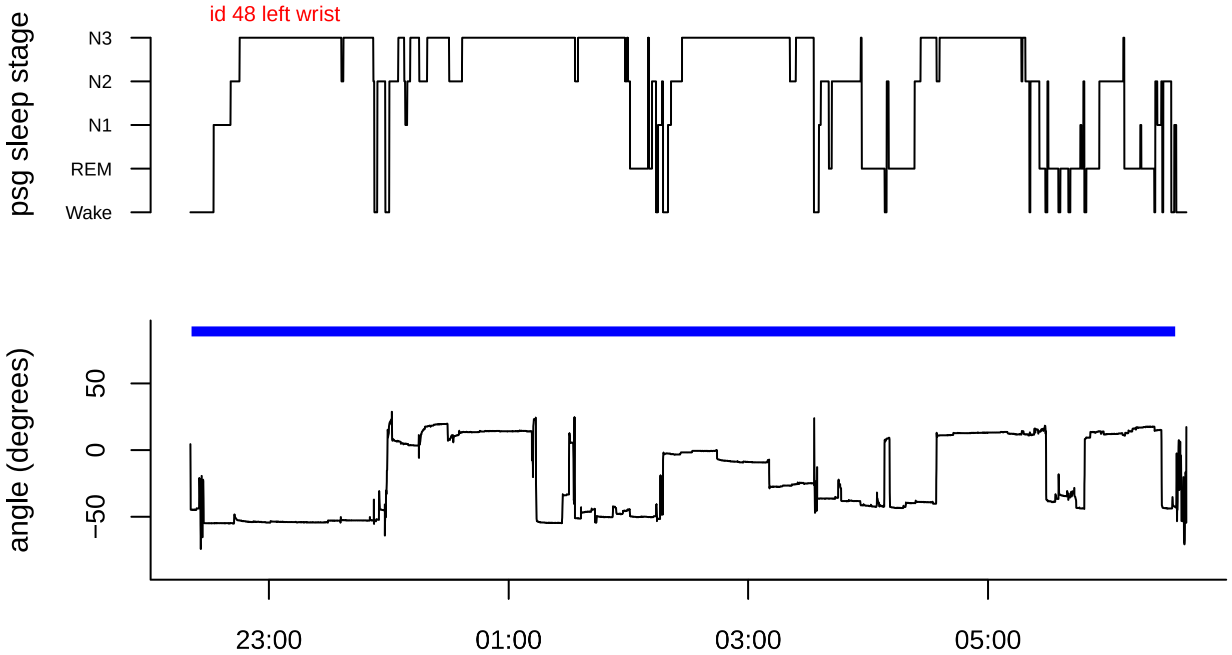

id 48 left wrist
psg sleep stage
N3
N2
N1
REM
Wake
angle (degrees)
50
0
−50
23:00
01:00
03:00
05:00

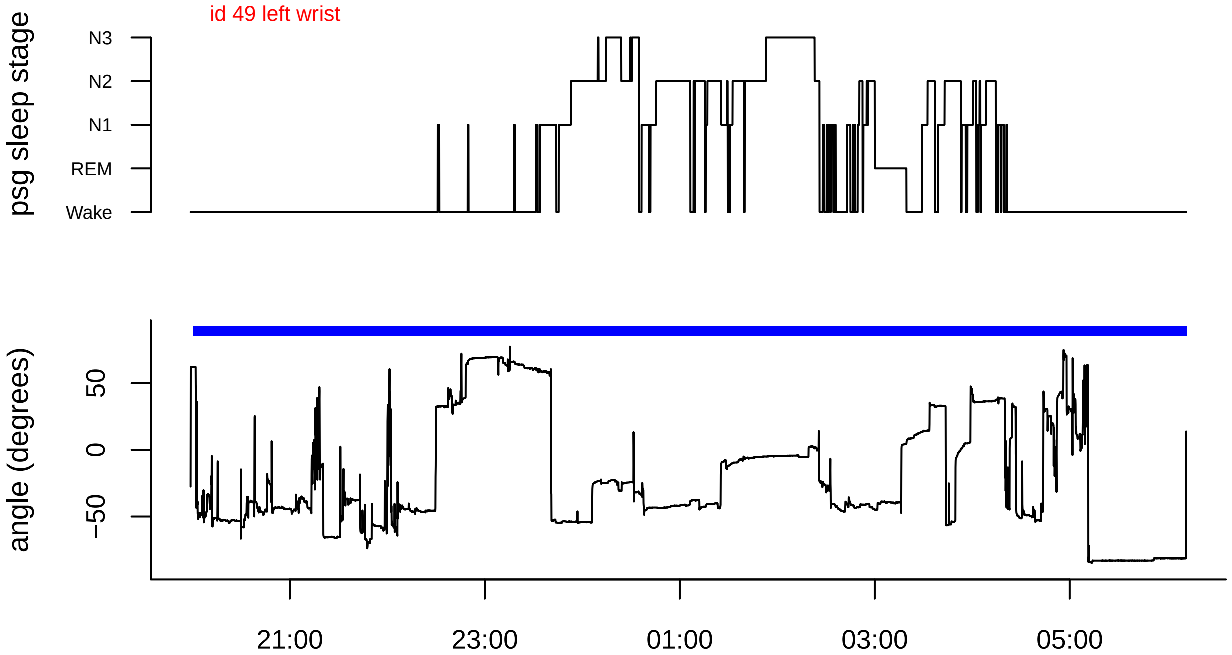
id 49 left wrist
psg sleep stage
N3
N2
N1
REM
Wake
angle (degrees)
50
0
−50
21:00
23:00
01:00
03:00
05:00

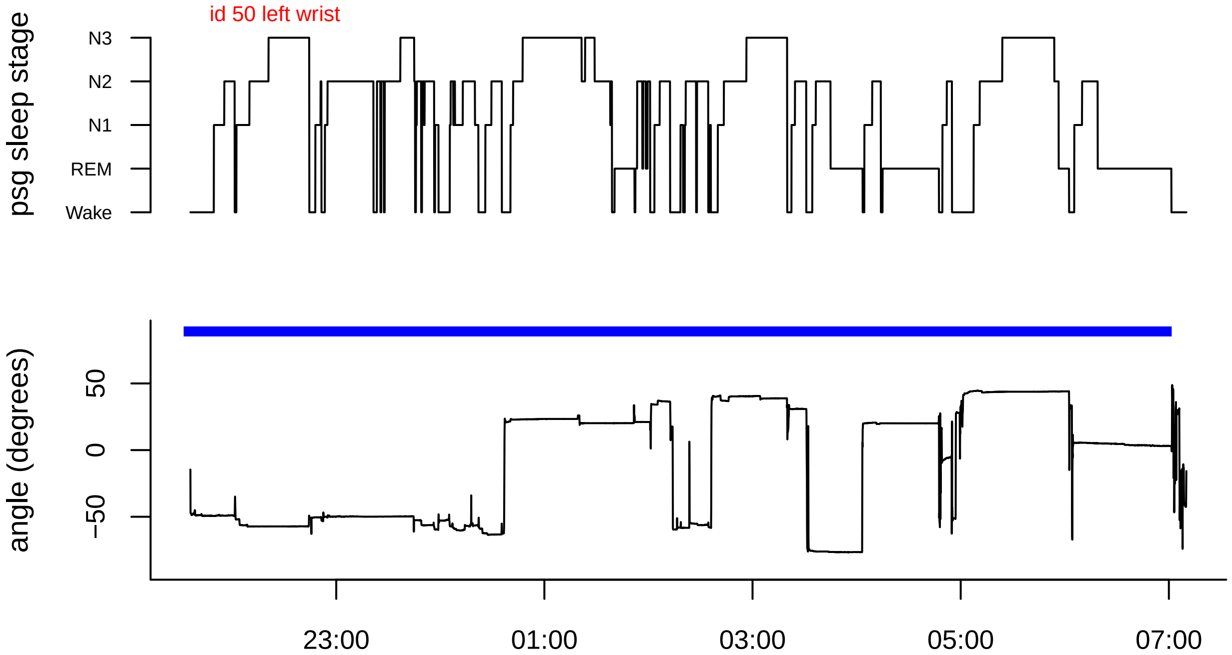
id 50 left wrist
psg sleep stage
N3
N2
N1
REM
Wake
angle (degrees)
50
0
−50
23:00
01:00
03:00
05:00
07:00

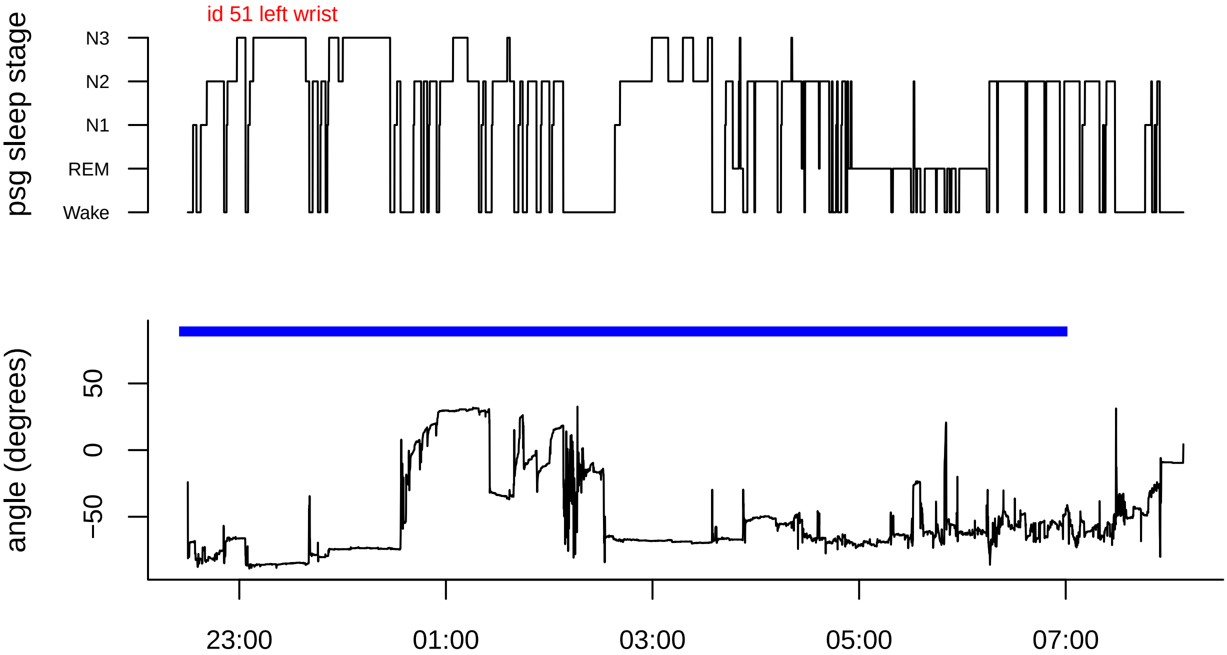
id 51 left wrist
psg sleep stage
N3
N2
N1
REM
Wake
angle (degrees)
50
0
−50
23:00
01:00
03:00
05:00
07:00

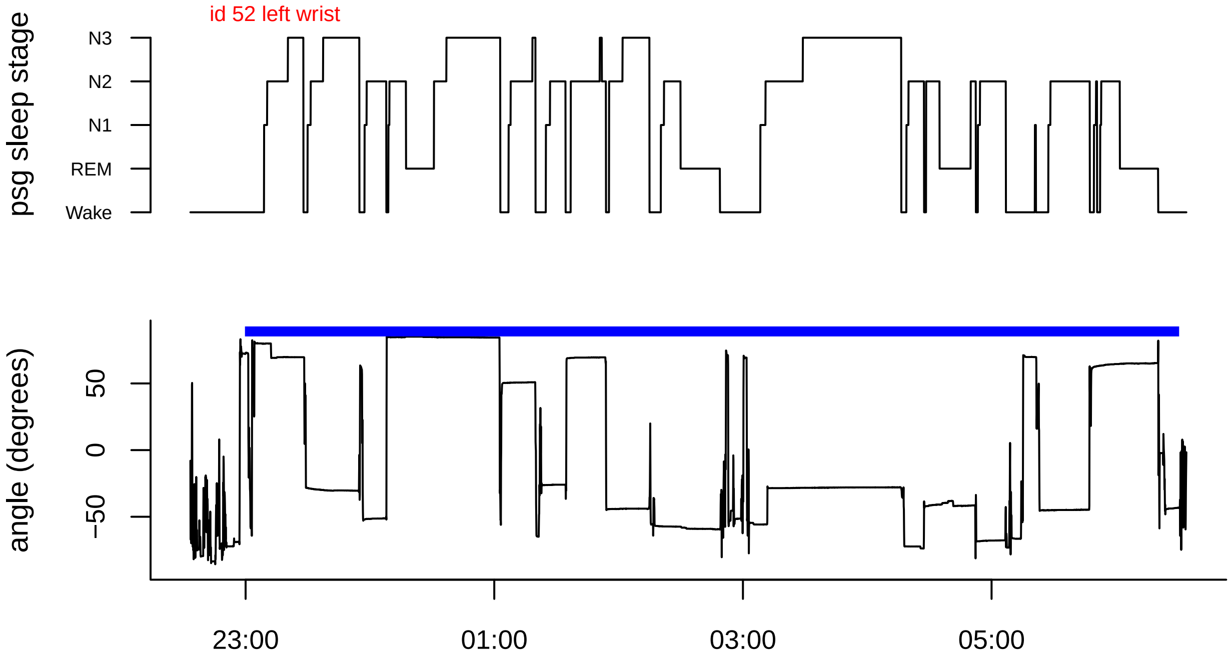
id 52 left wrist
psg sleep stage
N3
N2
N1
REM
Wake
angle (degrees)
50
0
−50
23:00
01:00
03:00
05:00

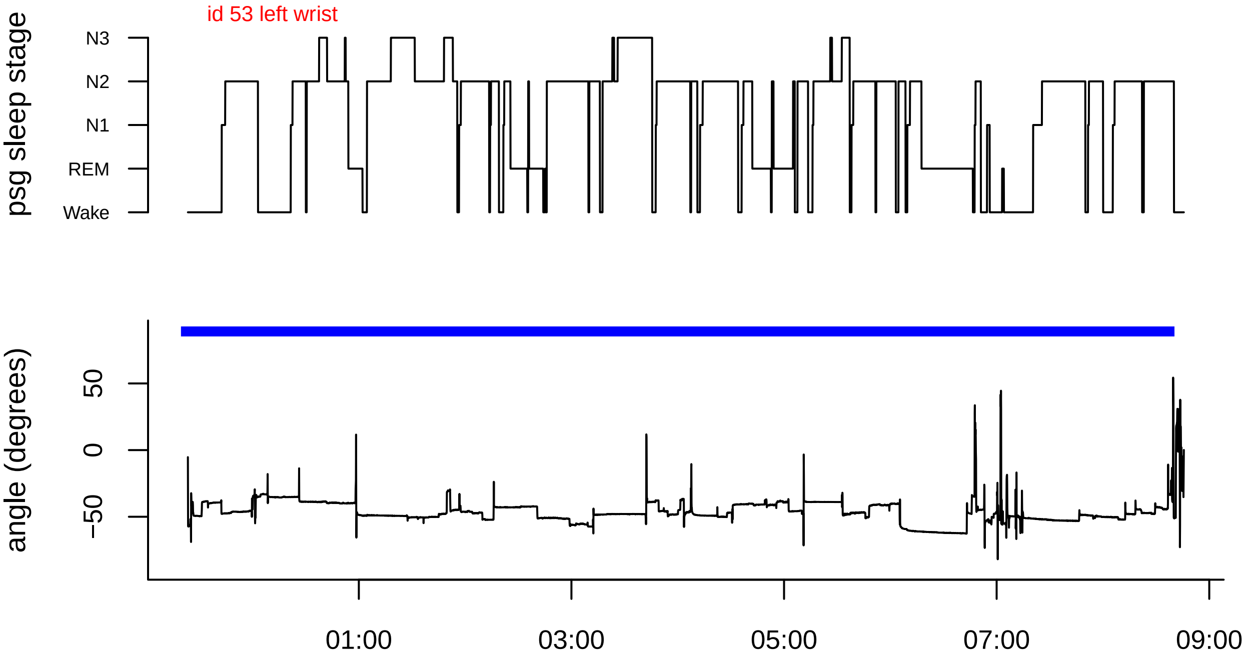

id 53 left wrist
psg sleep stage
N3
N2
N1
REM
Wake
angle (degrees)
50
0
−50
01:00
03:00
05:00
07:00
09:00

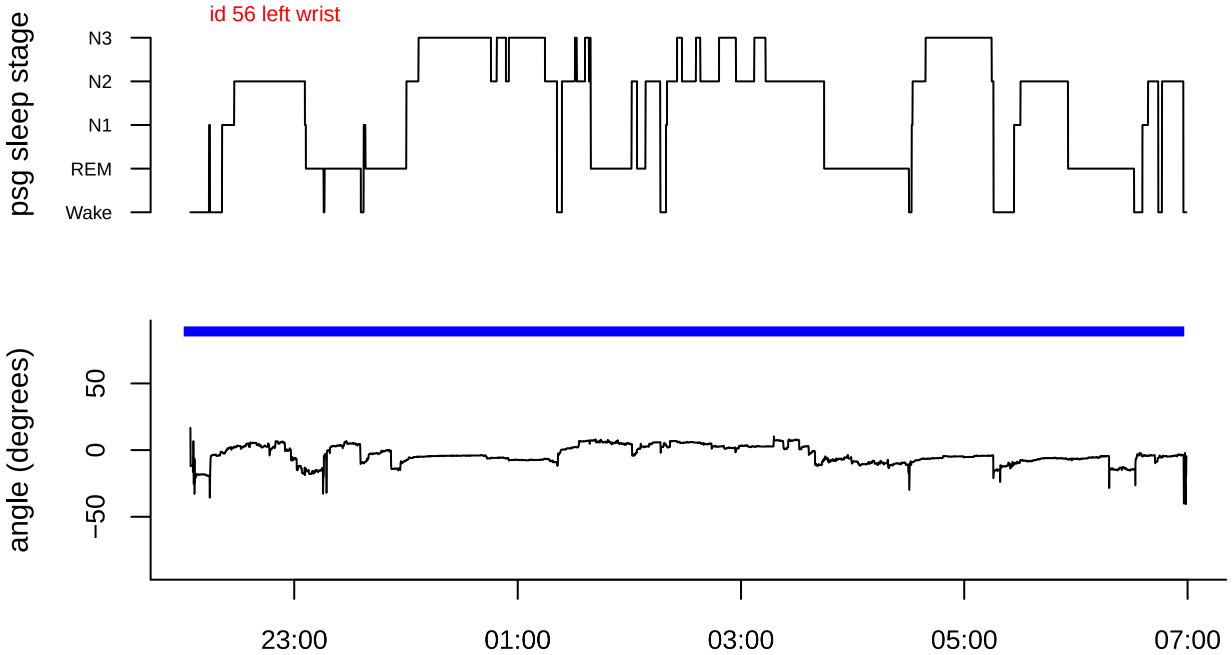

id 56 left wrist
psg sleep stage
N3
N2
N1
REM
Wake
angle (degrees)
50
0
−50
23:00
01:00
03:00
05:00
07:00

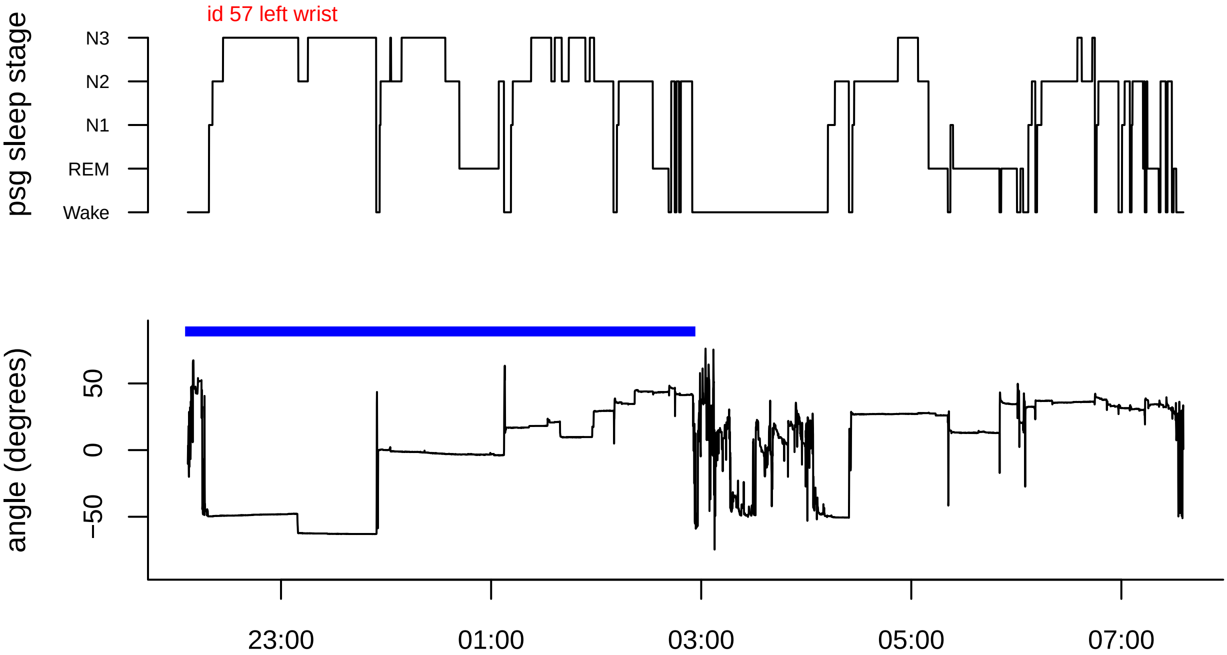

id 57 left wrist
psg sleep stage
N3
N2
N1
REM
Wake
angle (degrees)
50
0
−50
23:00
01:00
03:00
05:00
07:00

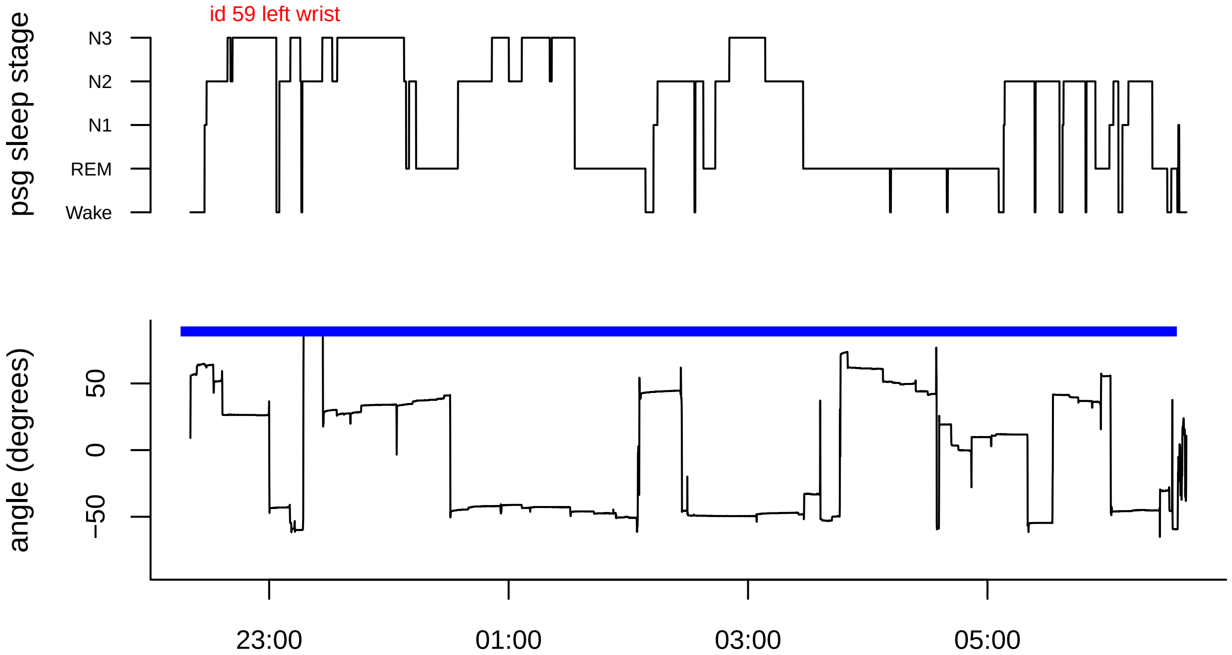

id 59 left wrist
psg sleep stage
N3
N2
N1
REM
Wake
angle (degrees)
50
0
−50
23:00
01:00
03:00
05:00

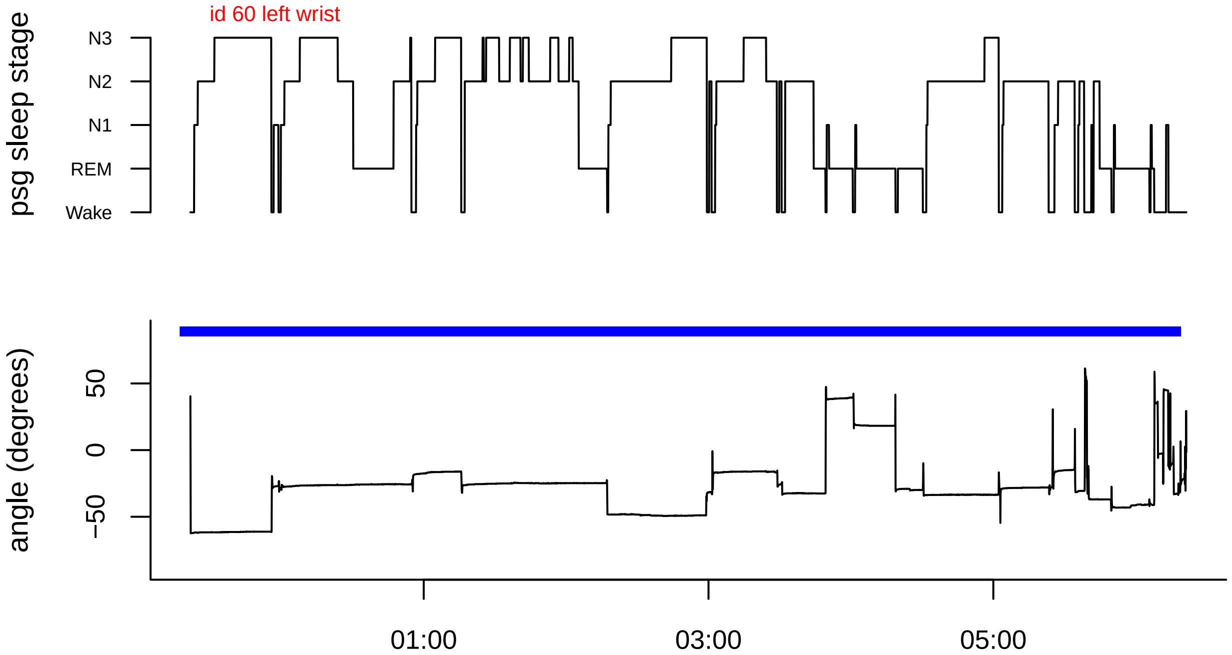

id 60 left wrist
psg sleep stage
N3
N2
N1
REM
Wake
angle (degrees)
50
0
−50
01:00
03:00
05:00

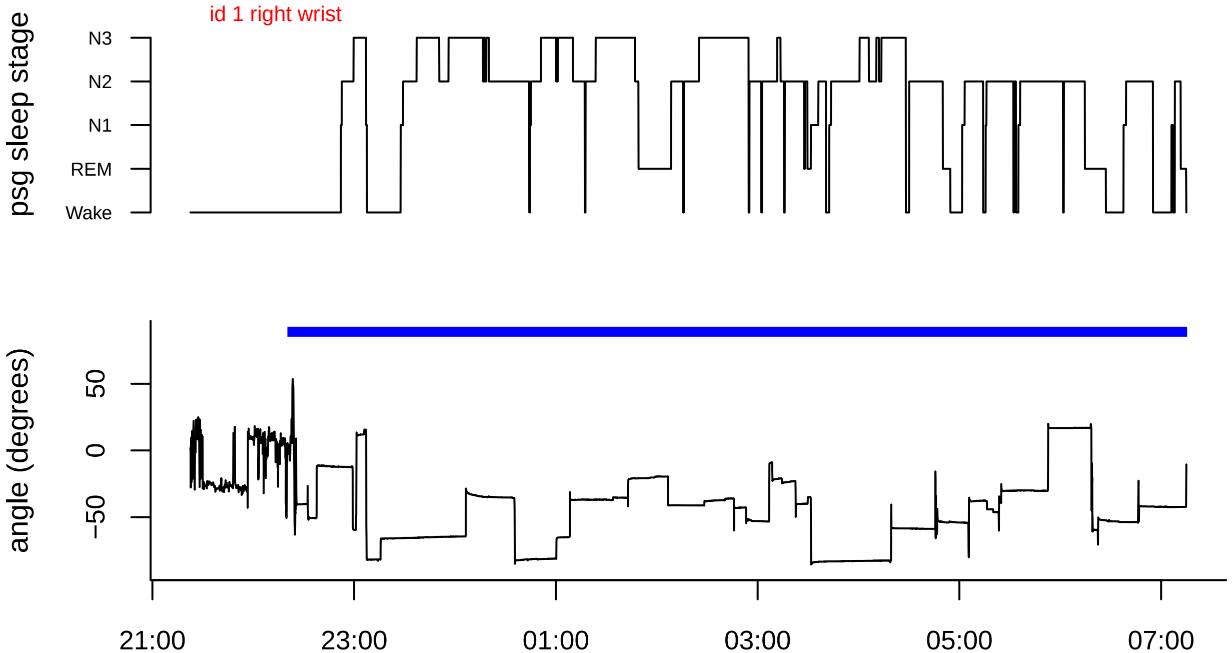

id 1 right wrist
psg sleep stage
N3
N2
N1
REM
Wake
angle (degrees)
50
0
−50
21:00
23:00
01:00
03:00
05:00
07:00

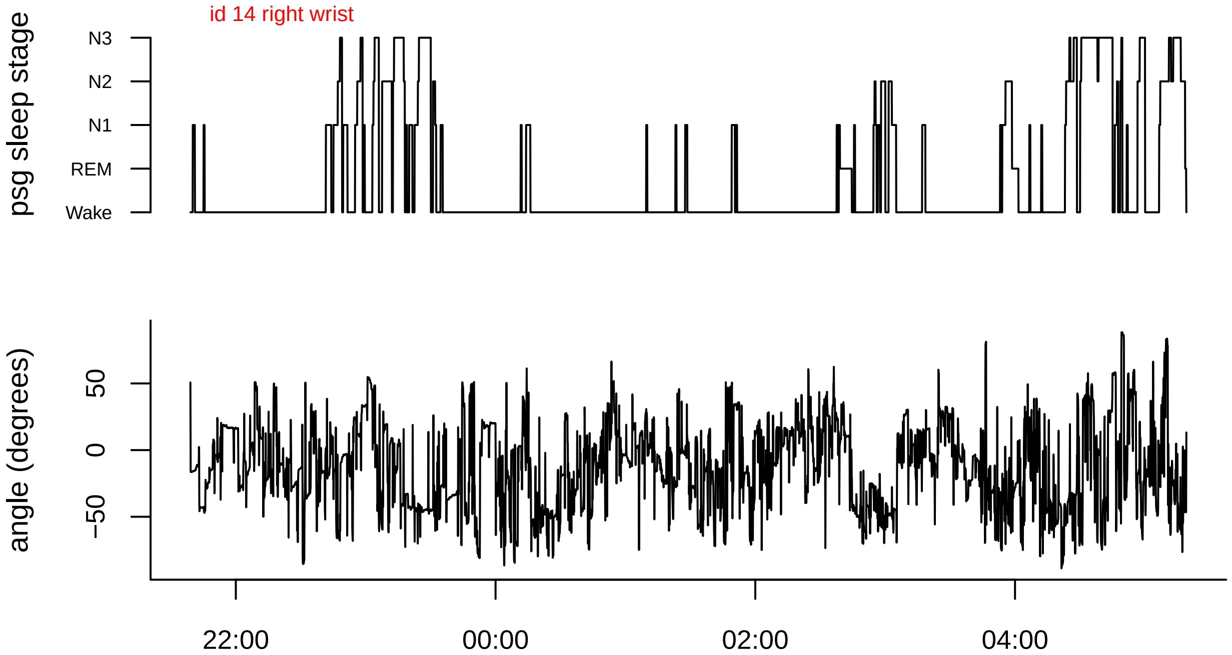

id 14 right wrist
psg sleep stage
N3
N2
N1
REM
Wake
angle (degrees)
50
0
−50
22:00
00:00
02:00
04:00

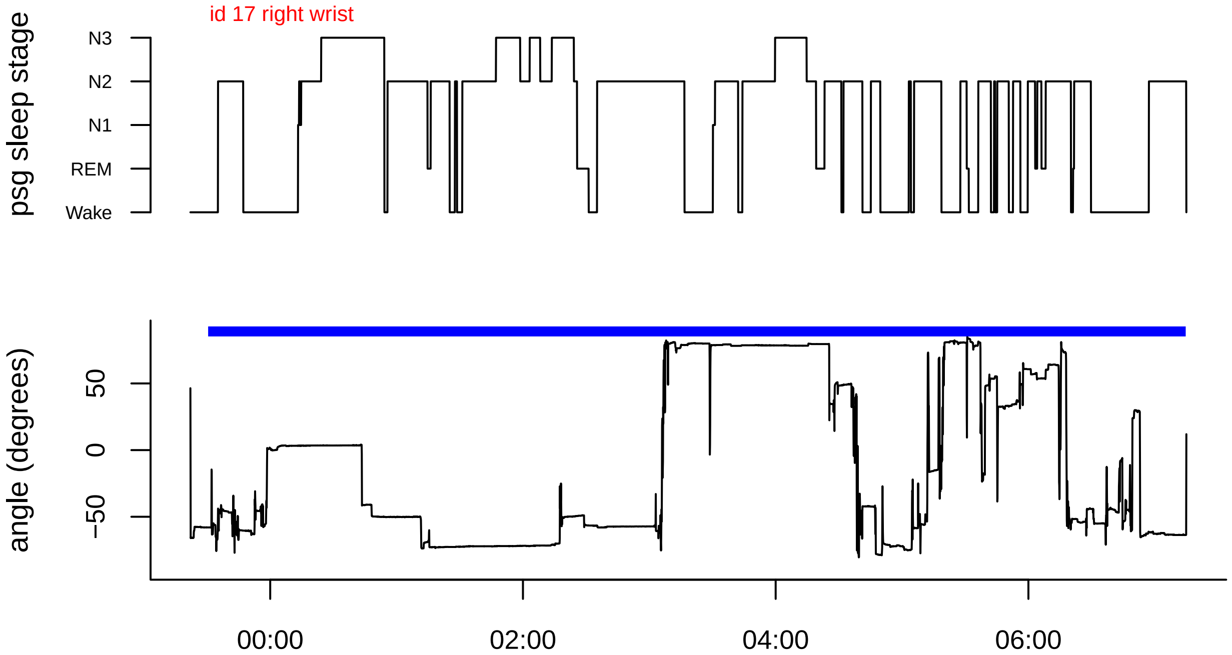

id 17 right wrist
psg sleep stage
N3
N2
N1
REM
Wake
angle (degrees)
50
0
−50
00:00
02:00
04:00
06:00

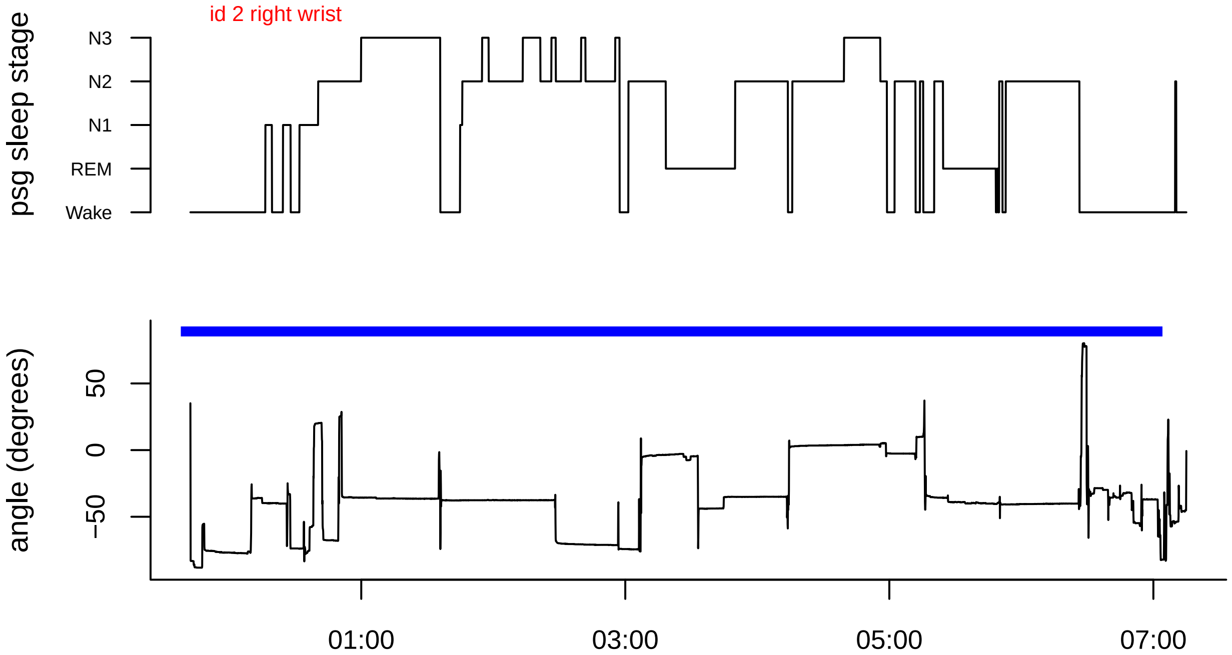
id 2 right wrist
psg sleep stage
N3
N2
N1
REM
Wake
angle (degrees)
50
0
−50
01:00
03:00
05:00
07:00

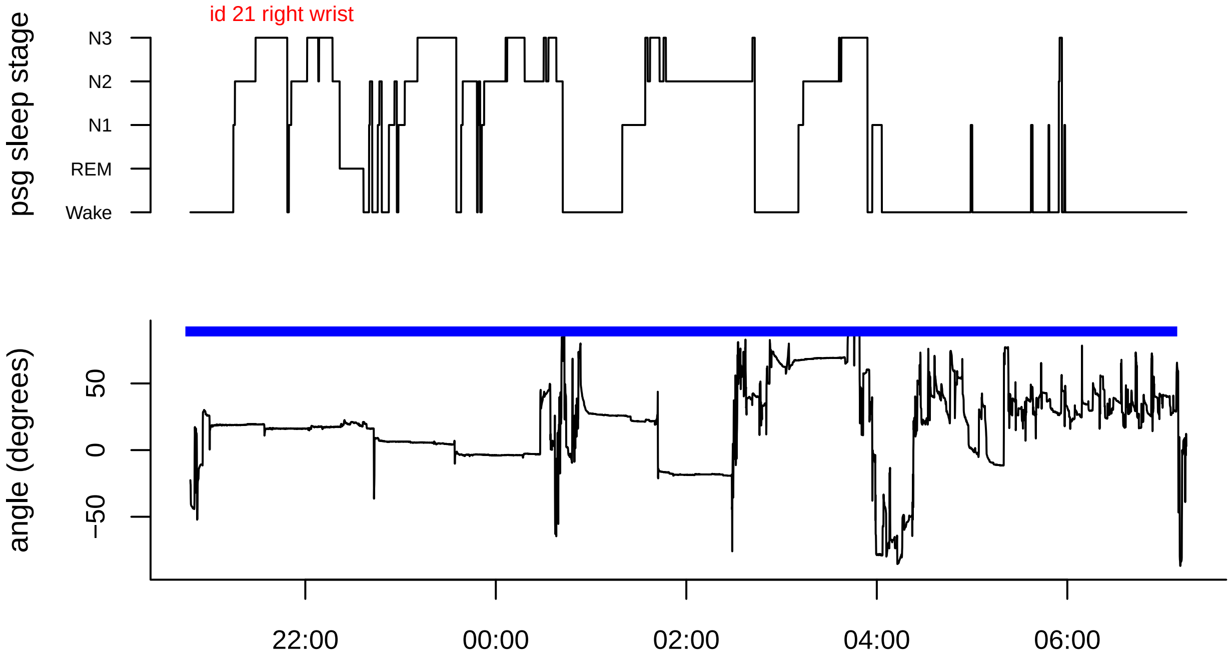
id 21 right wrist
psg sleep stage
N3
N2
N1
REM
Wake
angle (degrees)
50
0
−50
22:00
00:00
02:00
04:00
06:00

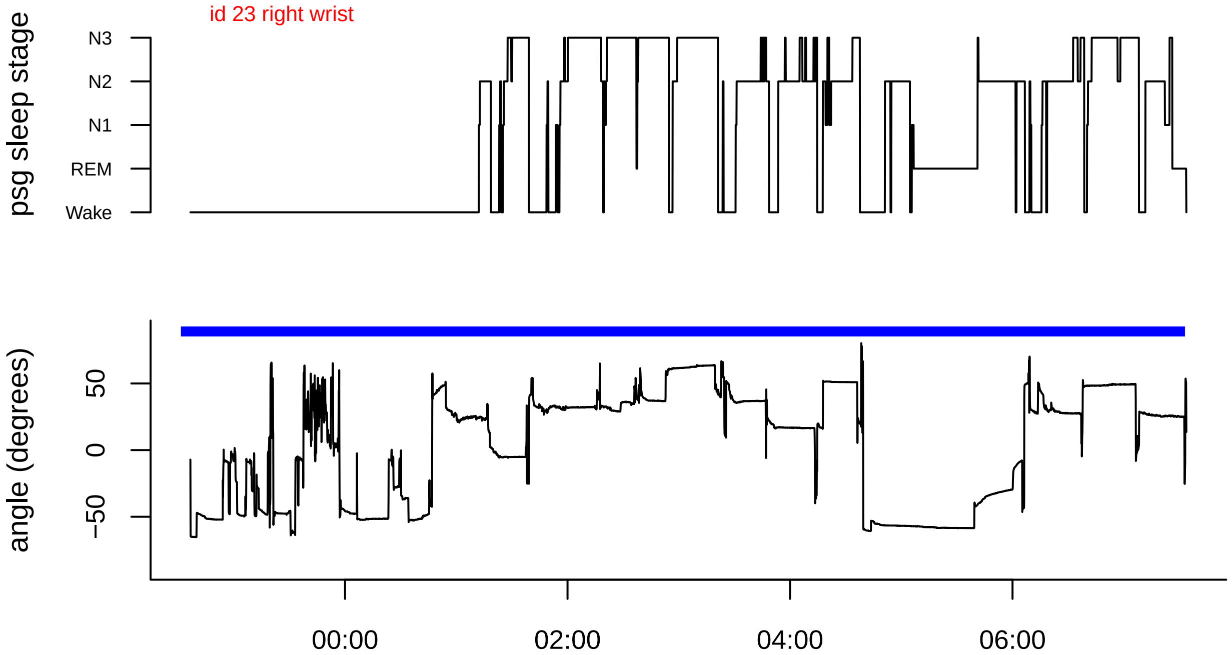

id 23 right wrist
psg sleep stage
N3
N2
N1
REM
Wake
angle (degrees)
50
0
−50
00:00
02:00
04:00
06:00

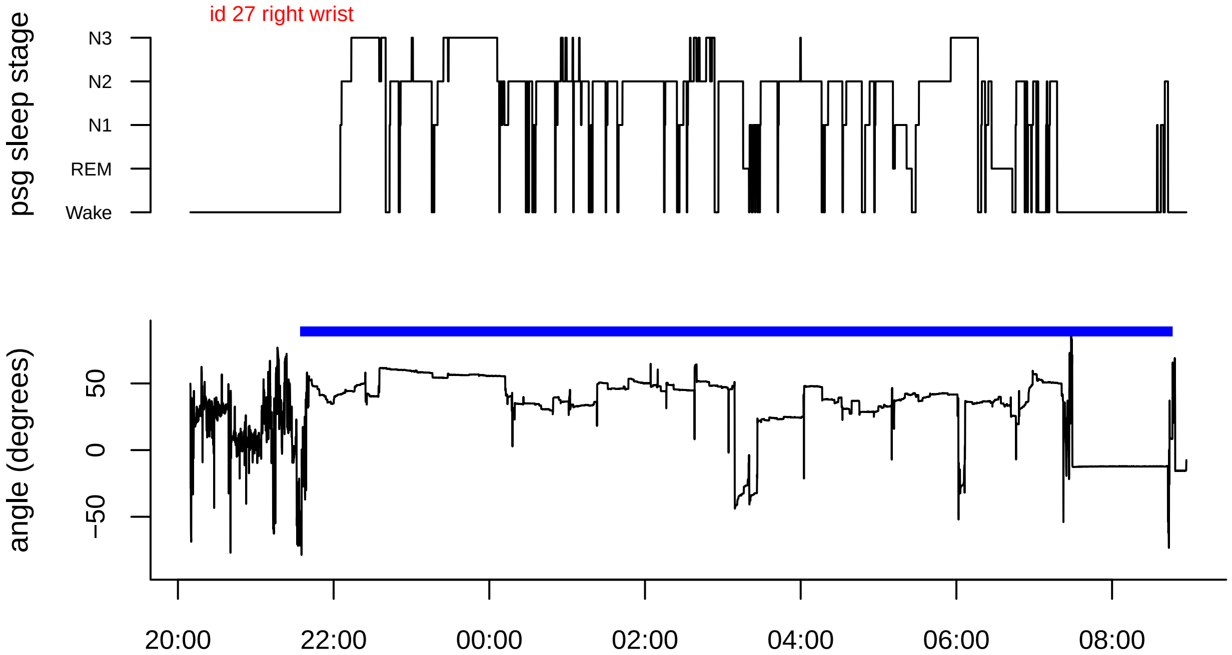

id 27 right wrist
psg sleep stage
N3
N2
N1
REM
Wake
angle (degrees)
50
0
−50
20:00
22:00
00:00
02:00
04:00
06:00
08:00

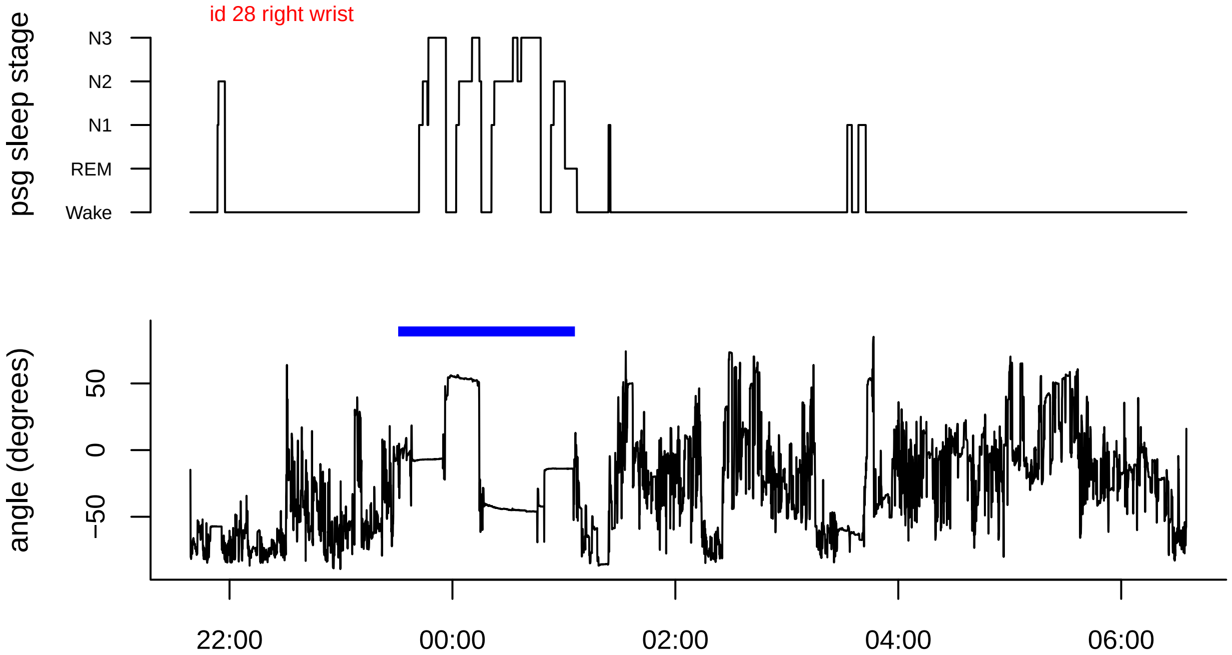

id 28 right wrist
psg sleep stage
N3
N2
N1
REM
Wake
angle (degrees)
50
0
−50
22:00
00:00
02:00
04:00
06:00

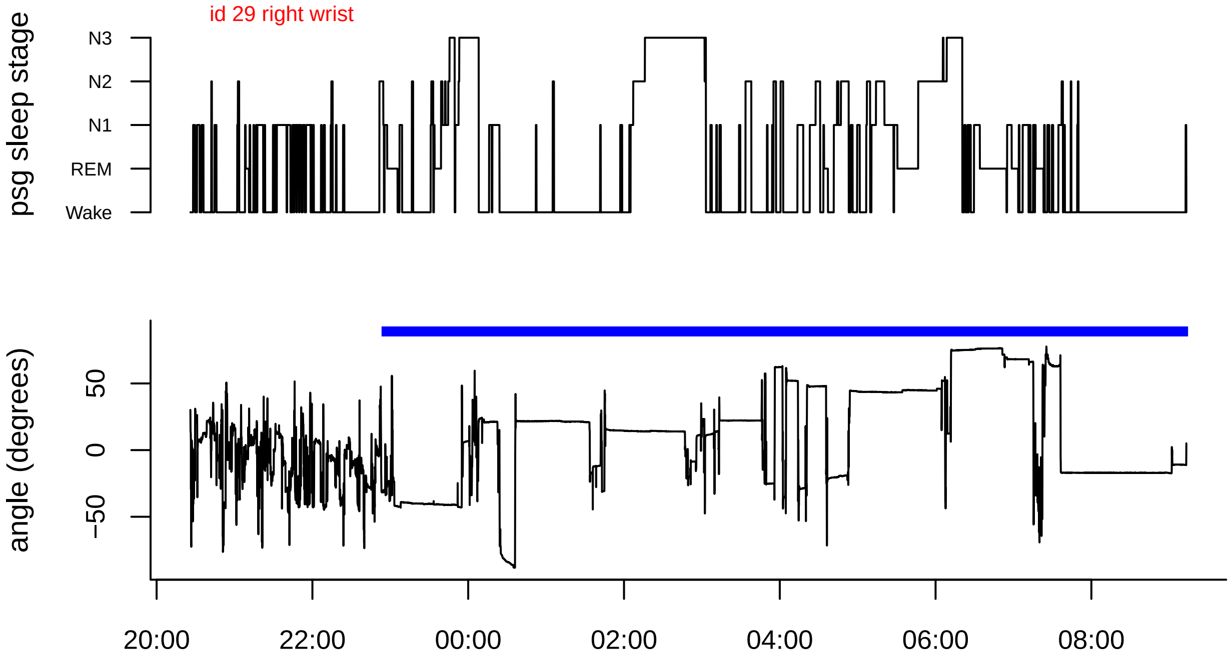
id 29 right wrist
psg sleep stage
N3
N2
N1
REM
Wake
angle (degrees)
50
0
−50
20:00
22:00
00:00
02:00
04:00
06:00
08:00

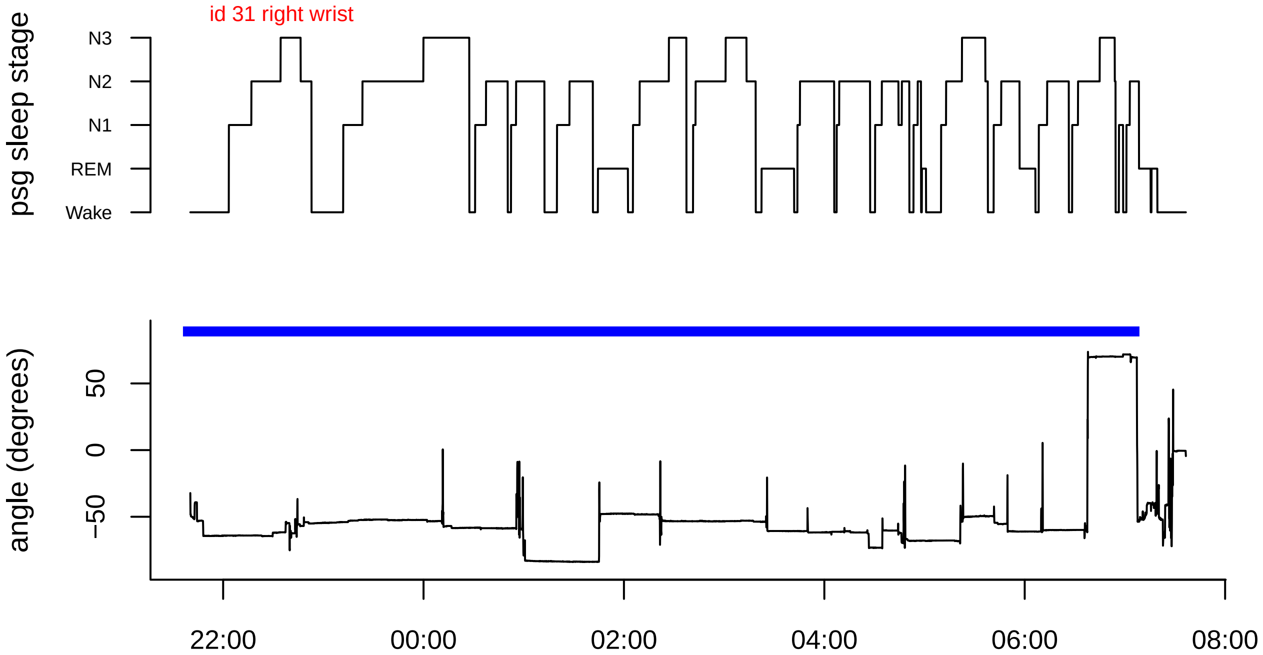
id 31 right wrist
psg sleep stage
N3
N2
N1
REM
Wake
angle (degrees)
50
0
-50
22:00
00:00
02:00
04:00
06:00
08:00

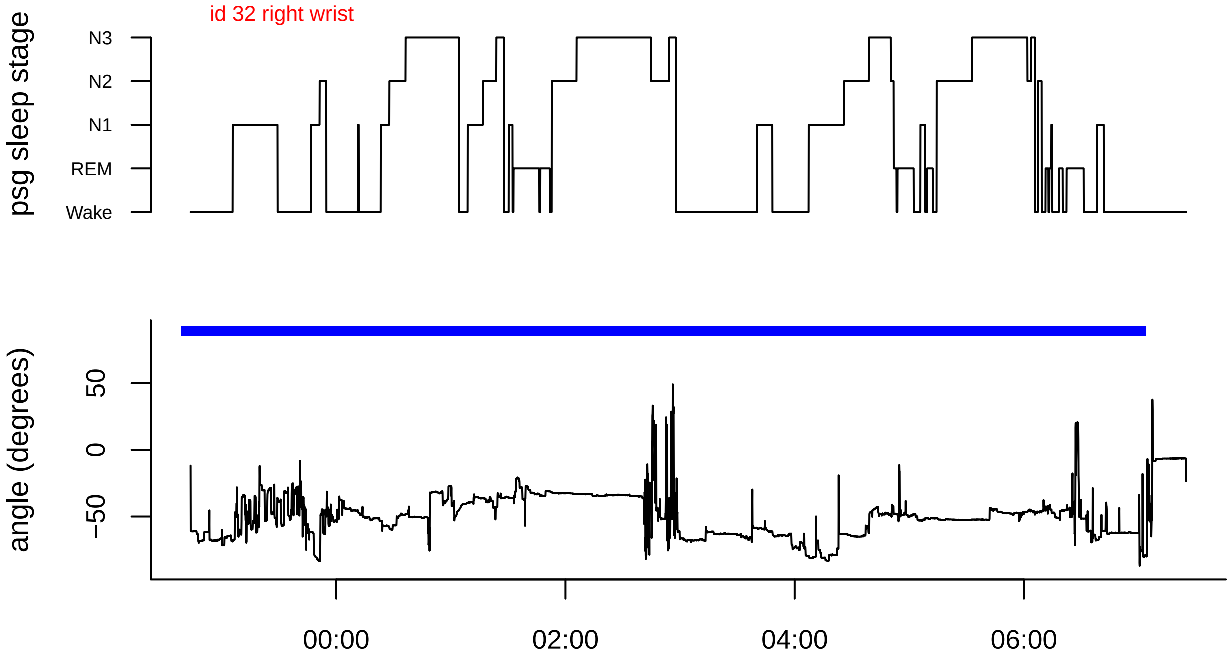

id 32 right wrist
psg sleep stage
N3
N2
N1
REM
Wake
angle (degrees)
50
0
-50
00:00
02:00
04:00
06:00

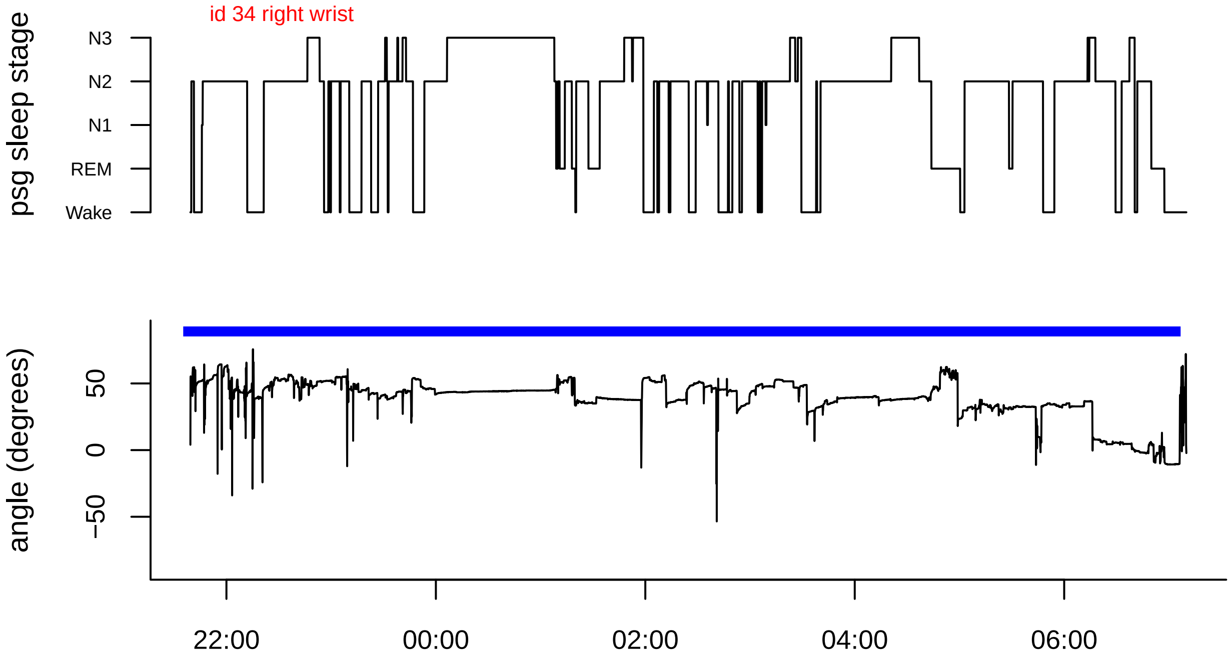
id 34 right wrist
psg sleep stage
N3
N2
N1
REM
Wake
angle (degrees)
50
0
−50
22:00
00:00
02:00
04:00
06:00

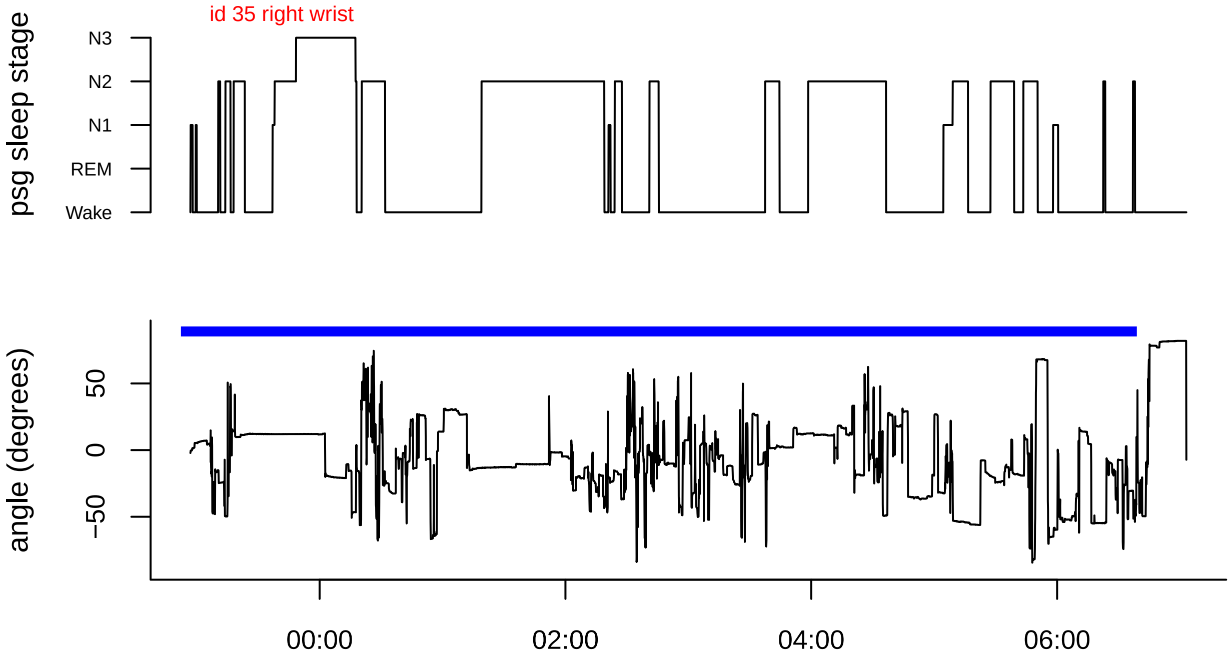

id 35 right wrist
psg sleep stage
N3
N2
N1
REM
Wake
angle (degrees)
50
0
−50
00:00
02:00
04:00
06:00

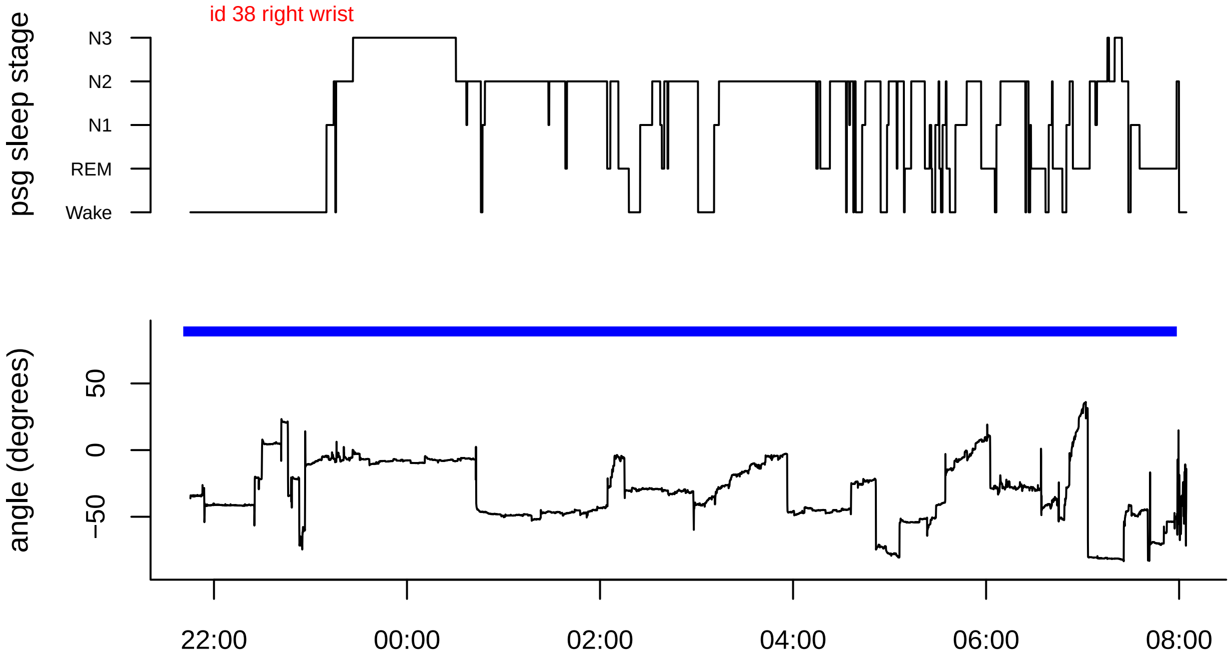

id 38 right wrist
psg sleep stage
N3
N2
N1
REM
Wake
angle (degrees)
50
0
−50
22:00
00:00
02:00
04:00
06:00
08:00

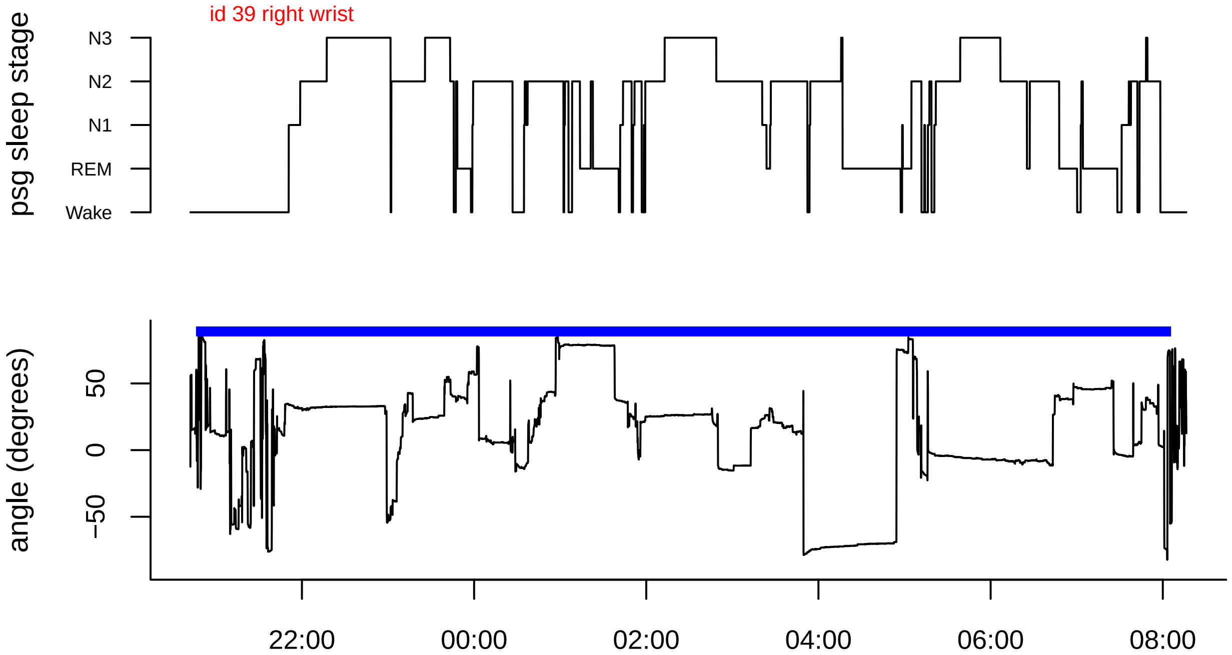
id 39 right wrist
psg sleep stage
N3
N2
N1
REM
Wake
angle (degrees)
50
0
−50
22:00
00:00
02:00
04:00
06:00
08:00

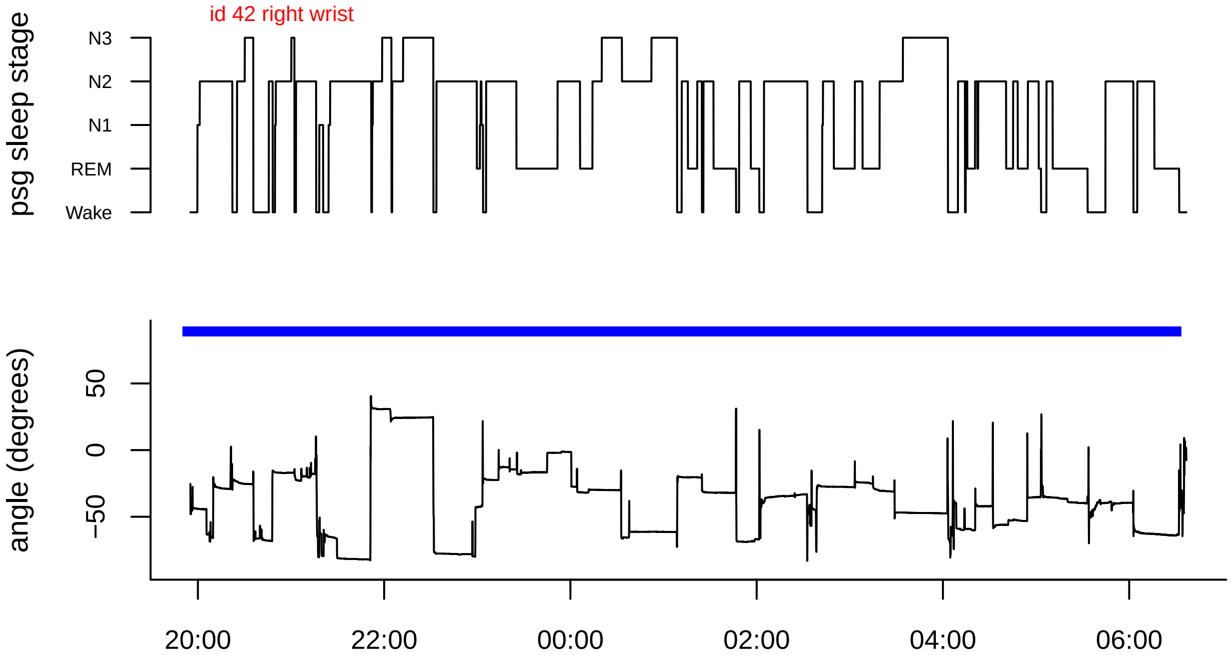

id 42 right wrist
psg sleep stage
N3
N2
N1
REM
Wake
angle (degrees)
50
0
−50
20:00
22:00
00:00
02:00
04:00
06:00

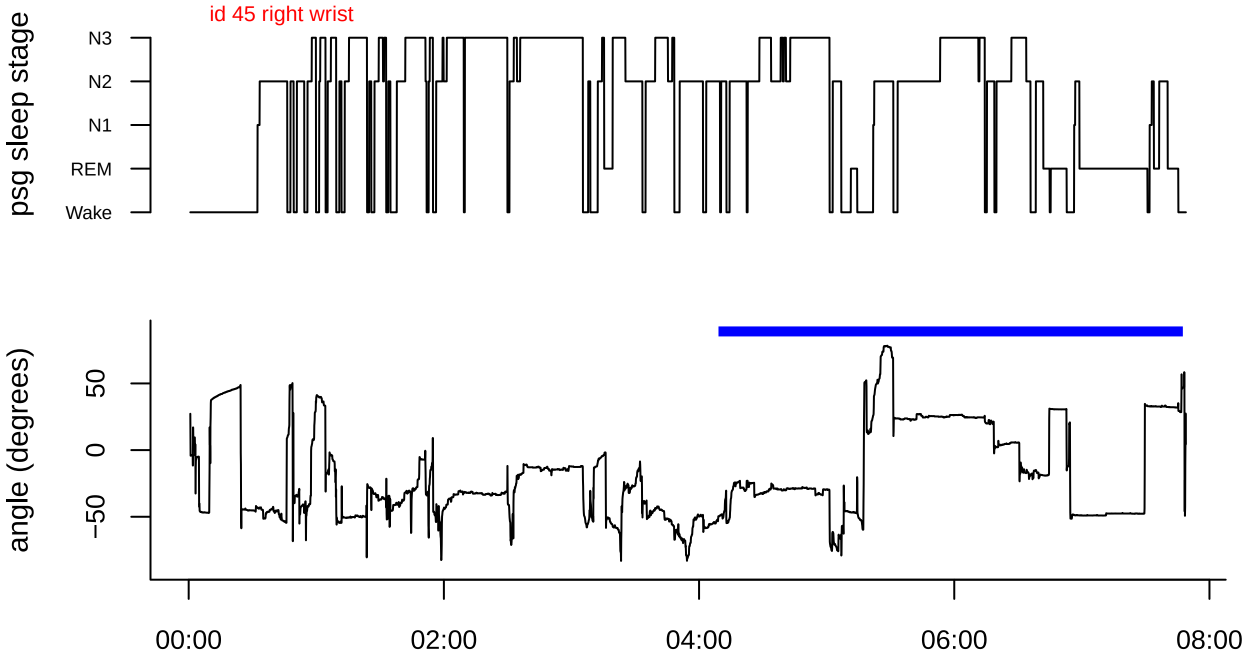

id 45 right wrist
psg sleep stage
N3
N2
N1
REM
Wake
angle (degrees)
50
0
−50
00:00
02:00
04:00
06:00
08:00

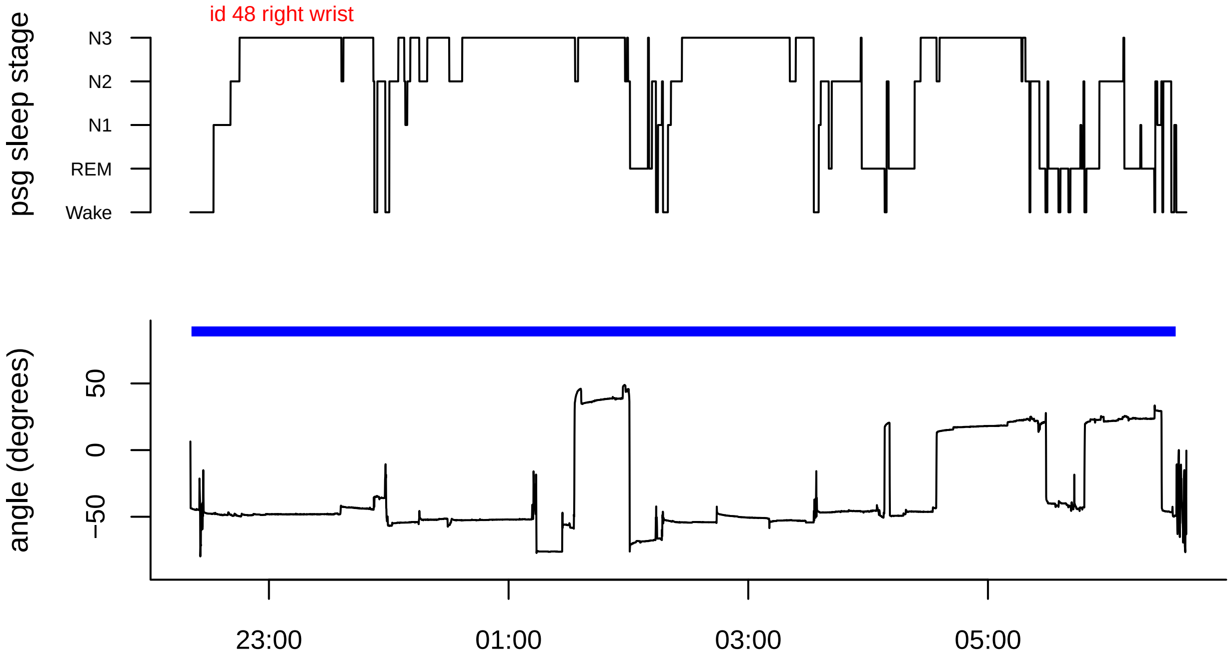

id 48 right wrist
psg sleep stage
N3
N2
N1
REM
Wake
angle (degrees)
50
0
−50
23:00
01:00
03:00
05:00

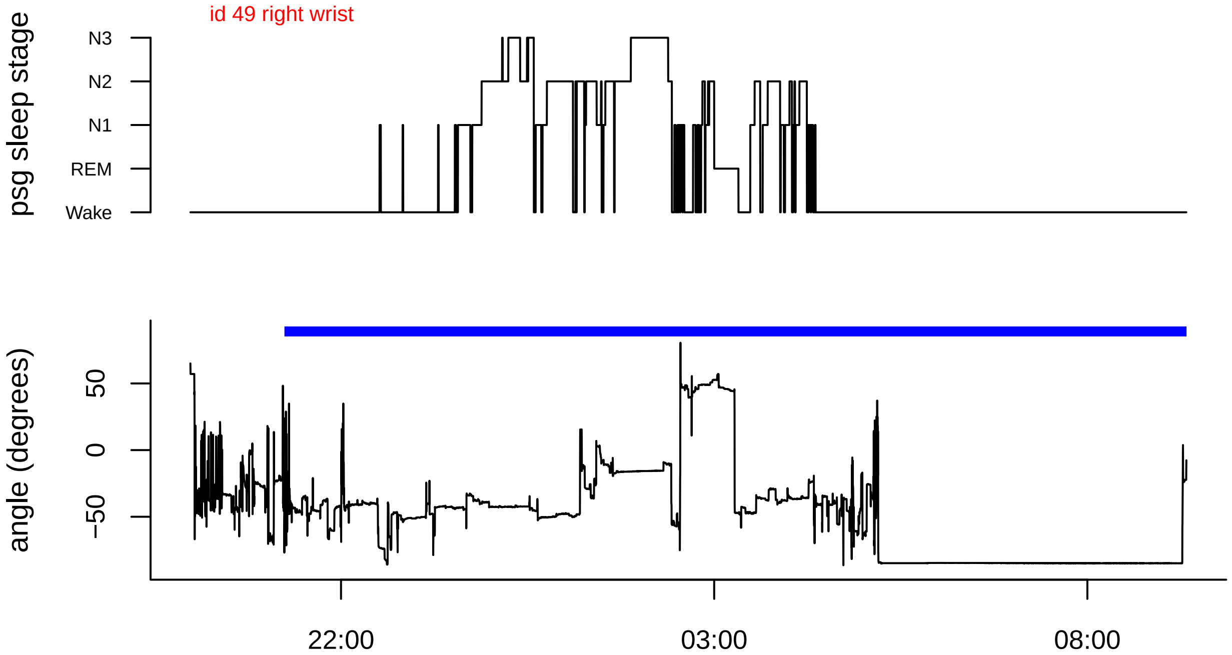

id 49 right wrist
psg sleep stage
N3
N2
N1
REM
Wake
angle (degrees)
50
0
−50
22:00
03:00
08:00

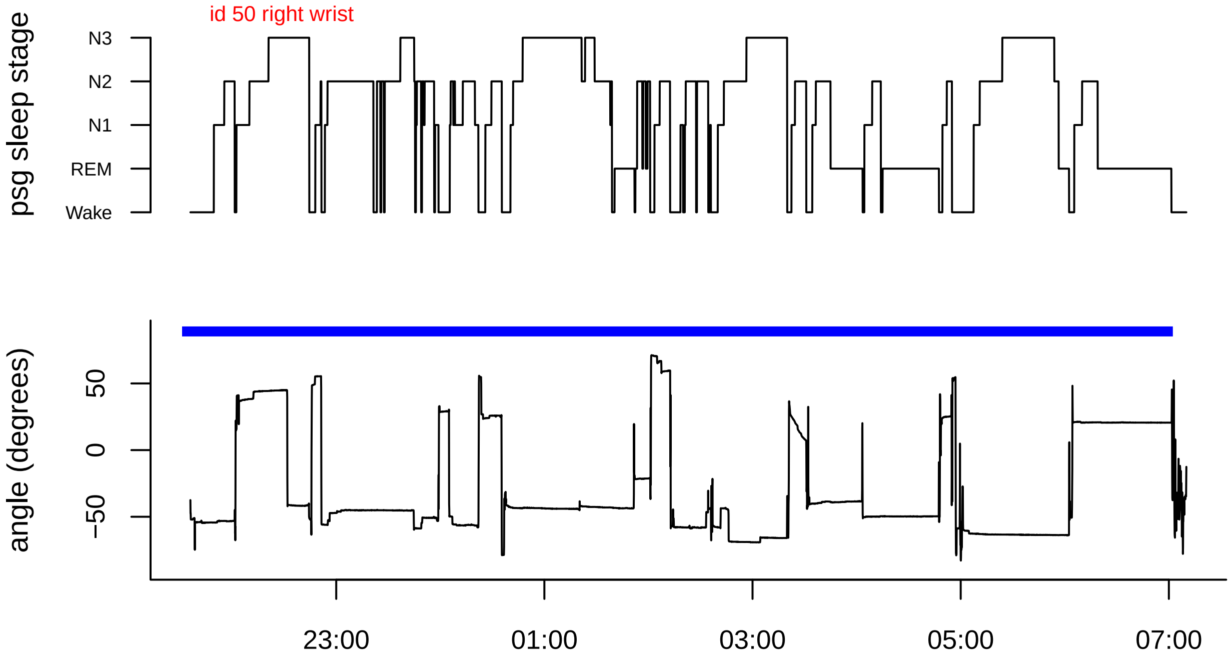

id 50 right wrist
psg sleep stage
N3
N2
N1
REM
Wake
angle (degrees)
50
0
−50
23:00
01:00
03:00
05:00
07:00

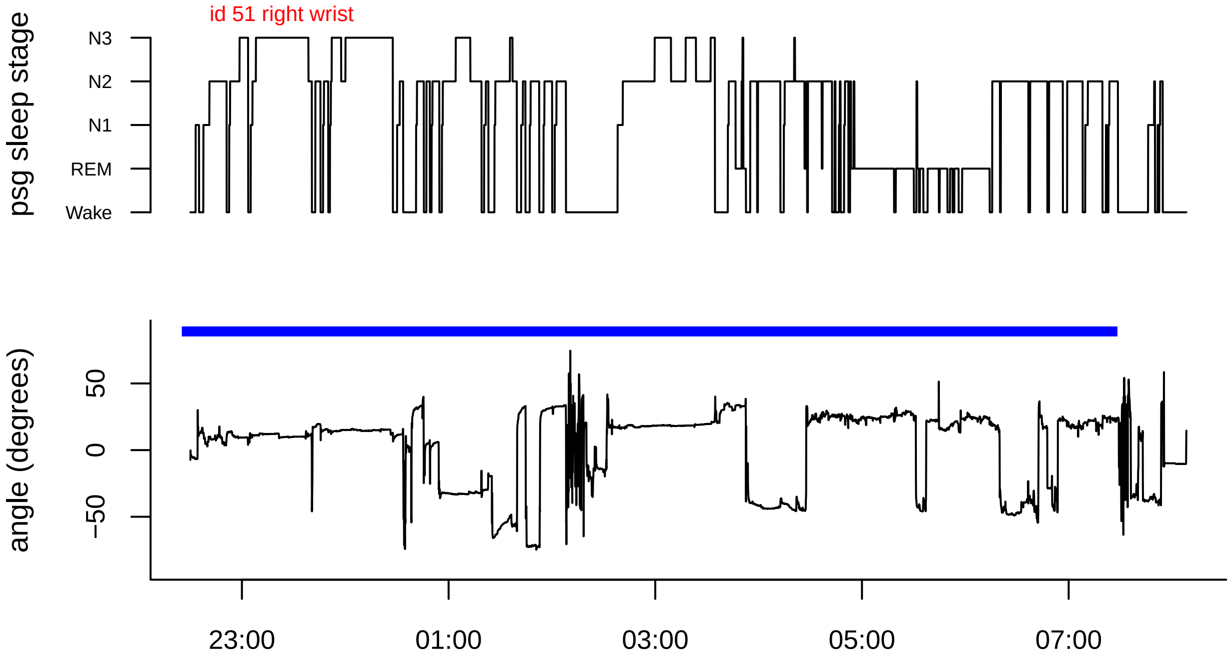
id 51 right wrist
psg sleep stage
N3
N2
N1
REM
Wake
angle (degrees)
50
0
−50
23:00
01:00
03:00
05:00
07:00

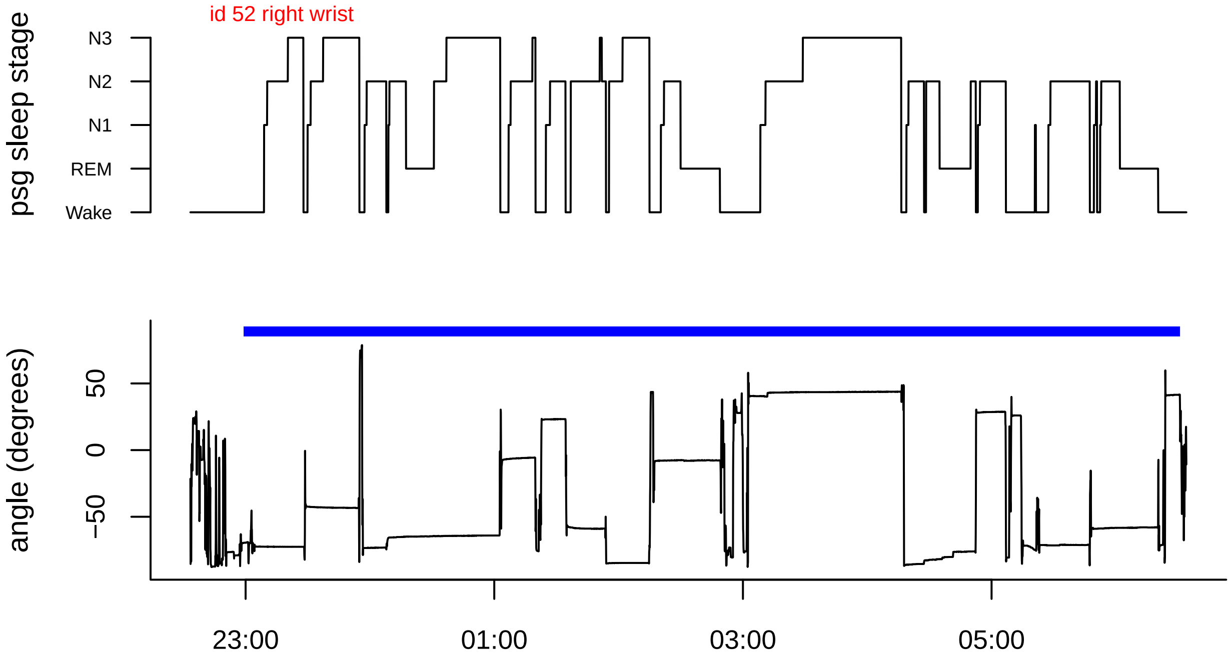
id 52 right wrist
psg sleep stage
N3
N2
N1
REM
Wake
angle (degrees)
50
0
−50
23:00
01:00
03:00
05:00

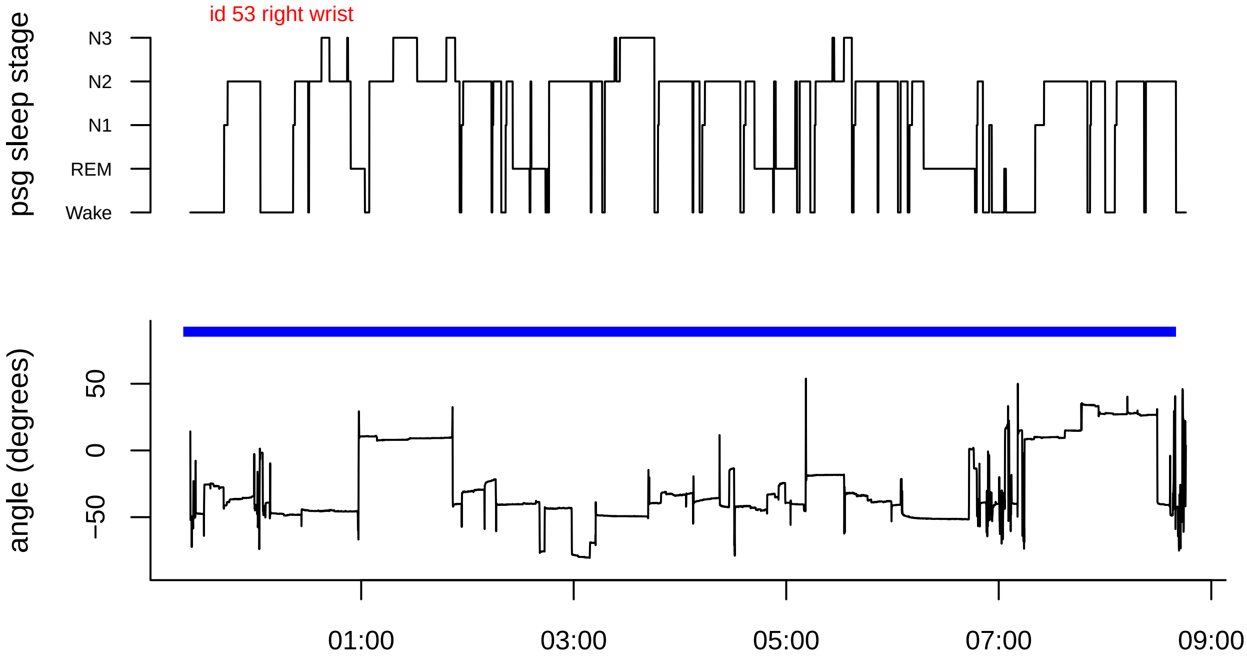
id 53 right wrist
psg sleep stage
N3
N2
N1
REM
Wake
angle (degrees)
50
0
−50
01:00
03:00
05:00
07:00
09:00

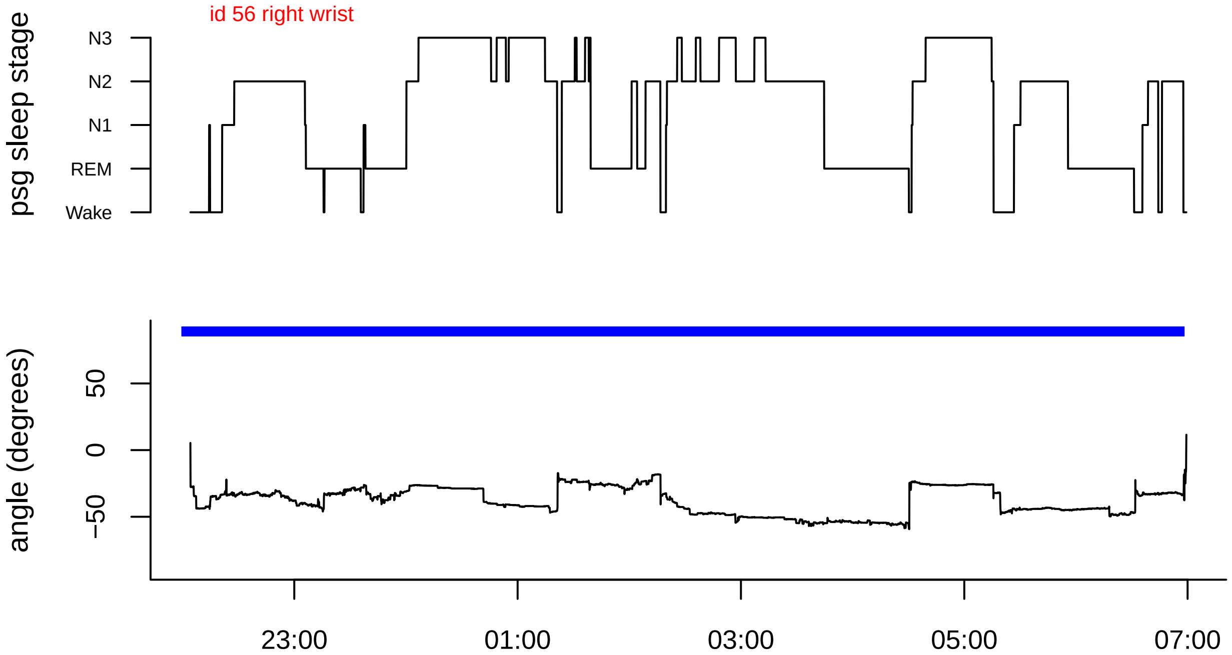

id 56 right wrist
psg sleep stage
N3
N2
N1
REM
Wake
angle (degrees)
50
0
−50
23:00
01:00
03:00
05:00
07:00

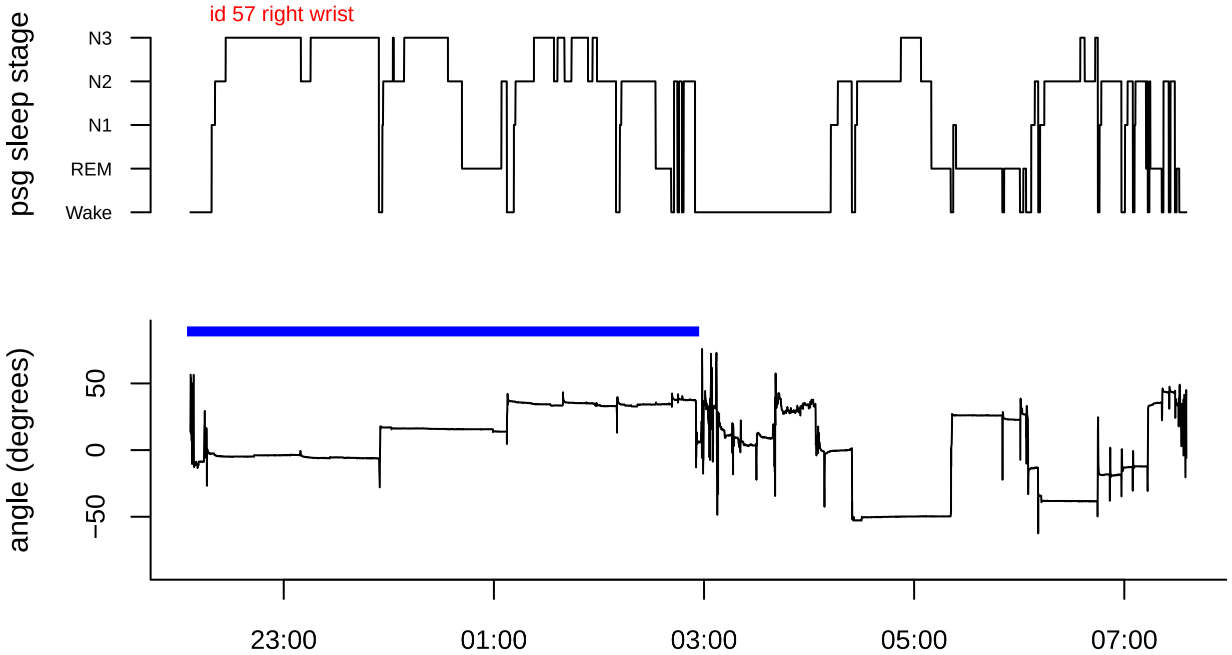
id 57 right wrist
psg sleep stage
N3
N2
N1
REM
Wake
angle (degrees)
50
0
−50
23:00
01:00
03:00
05:00
07:00

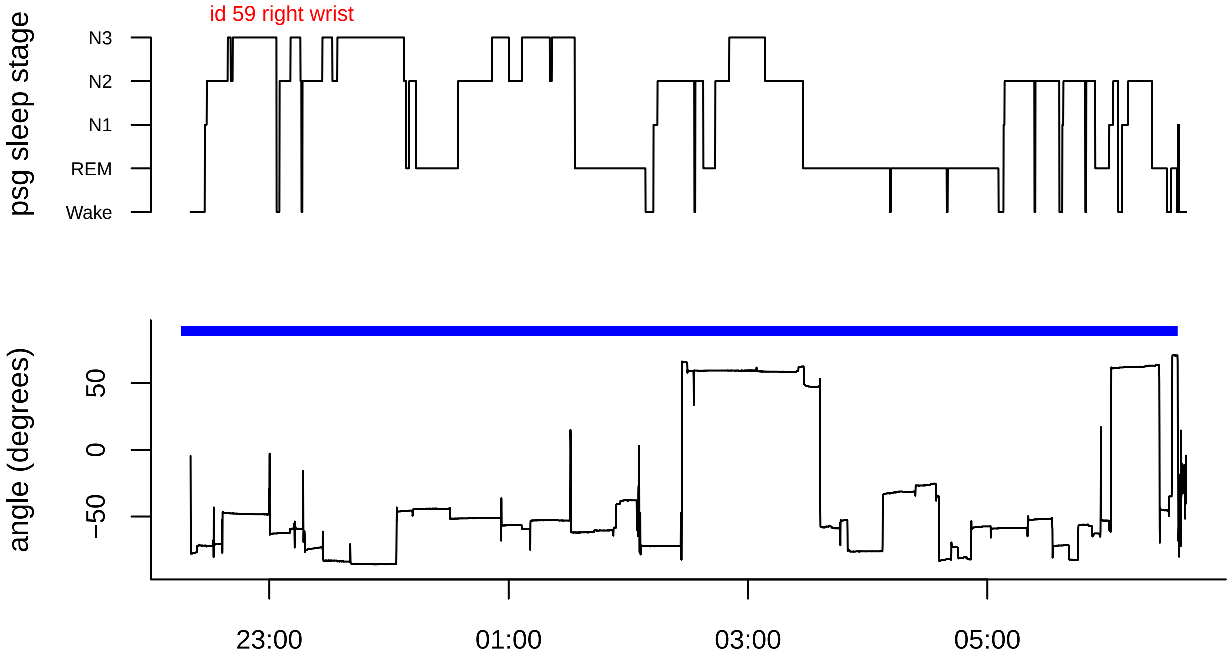

id 59 right wrist
psg sleep stage
N3
N2
N1
REM
Wake
angle (degrees)
50
0
−50
23:00
01:00
03:00
05:00

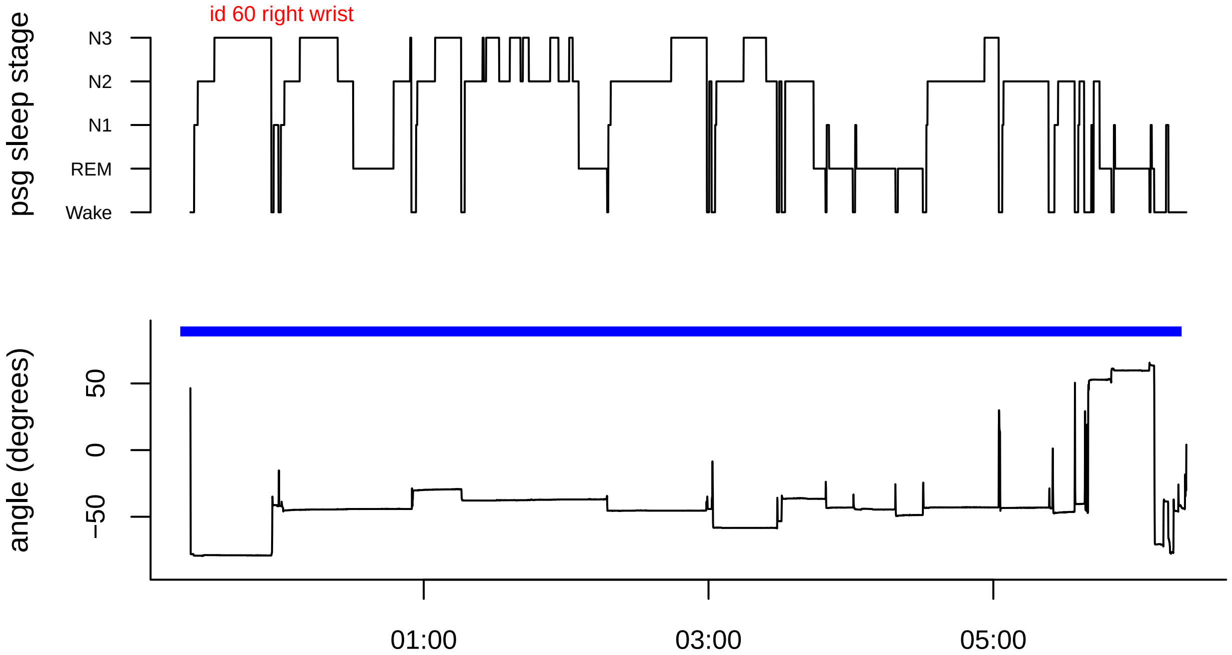
id 60 right wrist
psg sleep stage
N3
N2
N1
REM
Wake
angle (degrees)
50
0
−50
01:00
03:00
05:00

2 PSG healthy good sleepers (Pennsylvania) with accelerometer classifcation

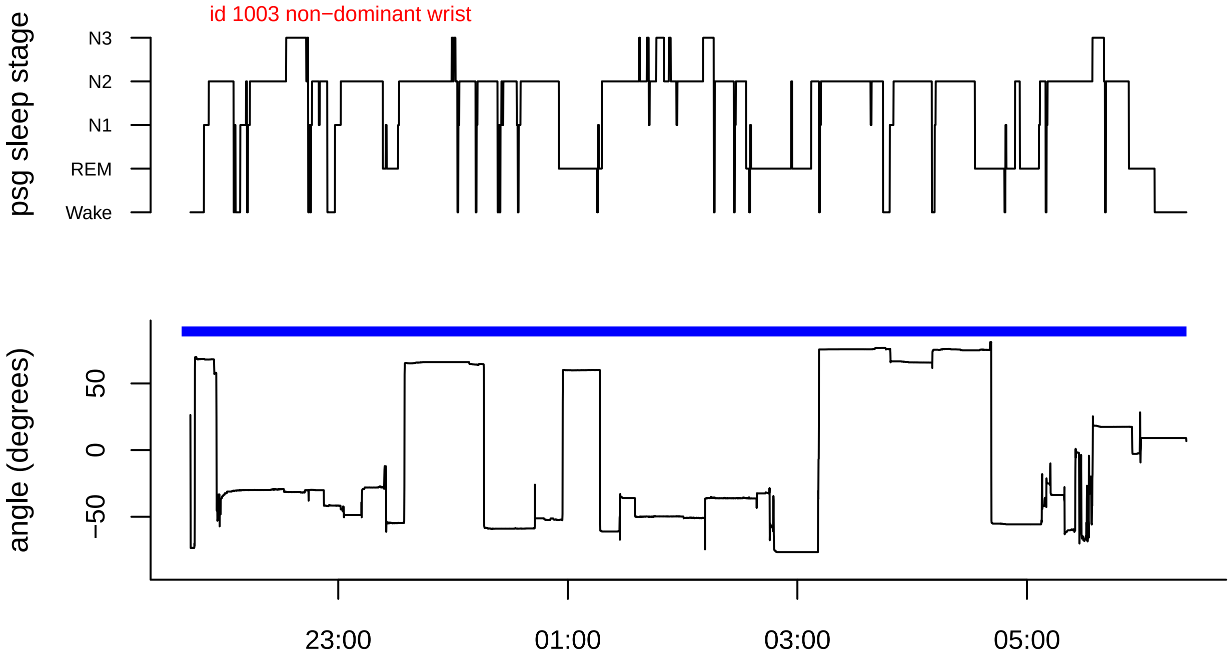

id 1003 non−dominant wrist
psg sleep stage
N3
N2
N1
REM
Wake
angle (degrees)
50
0
−50
23:00
01:00
03:00
05:00

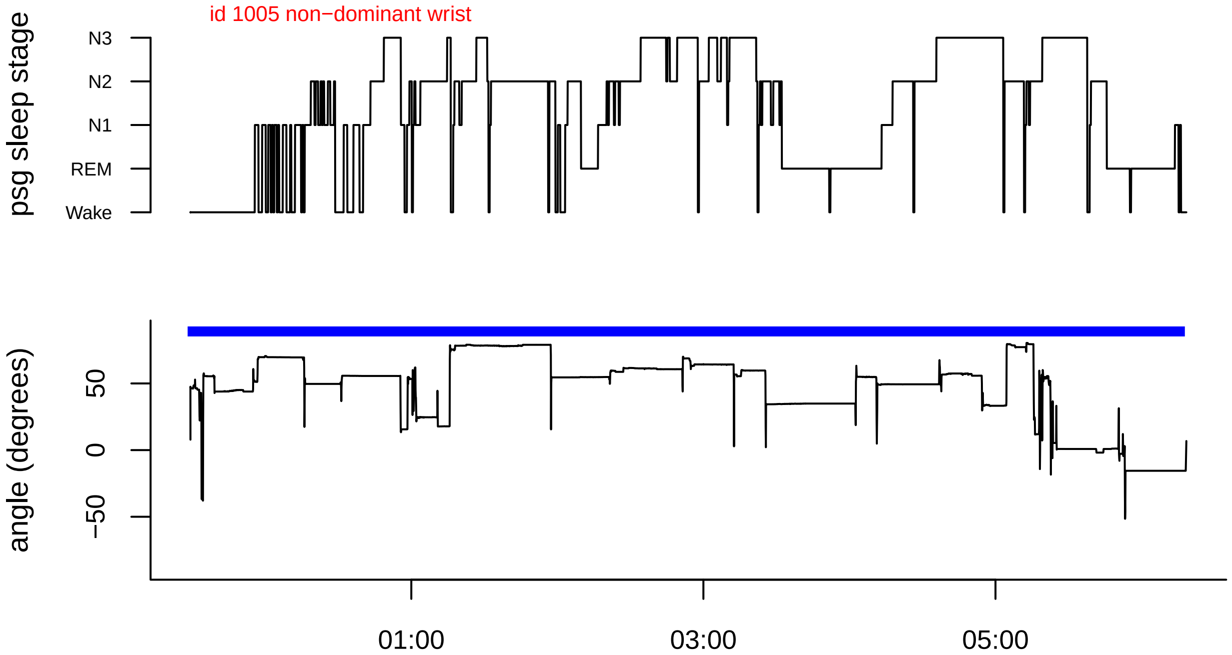
id 1005 non−dominant wrist
psg sleep stage
N3
N2
N1
REM
Wake
angle (degrees)
50
0
−50
01:00
03:00
05:00

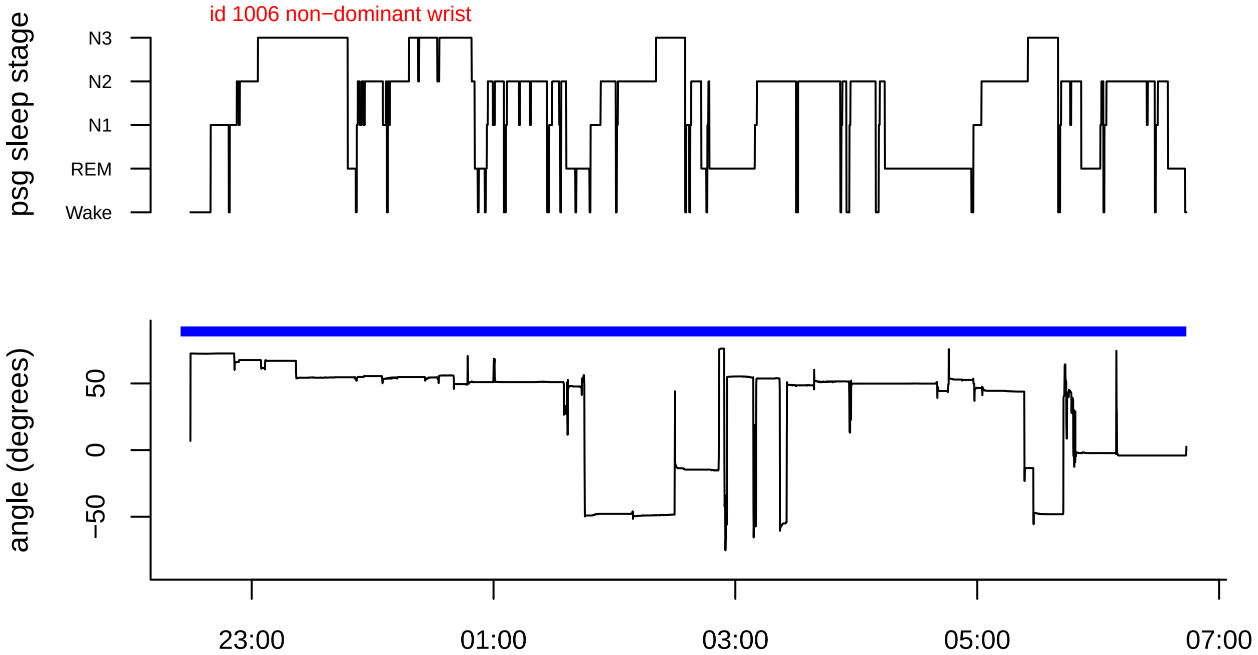
id 1006 non−dominant wrist
psg sleep stage
N3
N2
N1
REM
Wake
angle (degrees)
50
0
−50
23:00
01:00
03:00
05:00
07:00

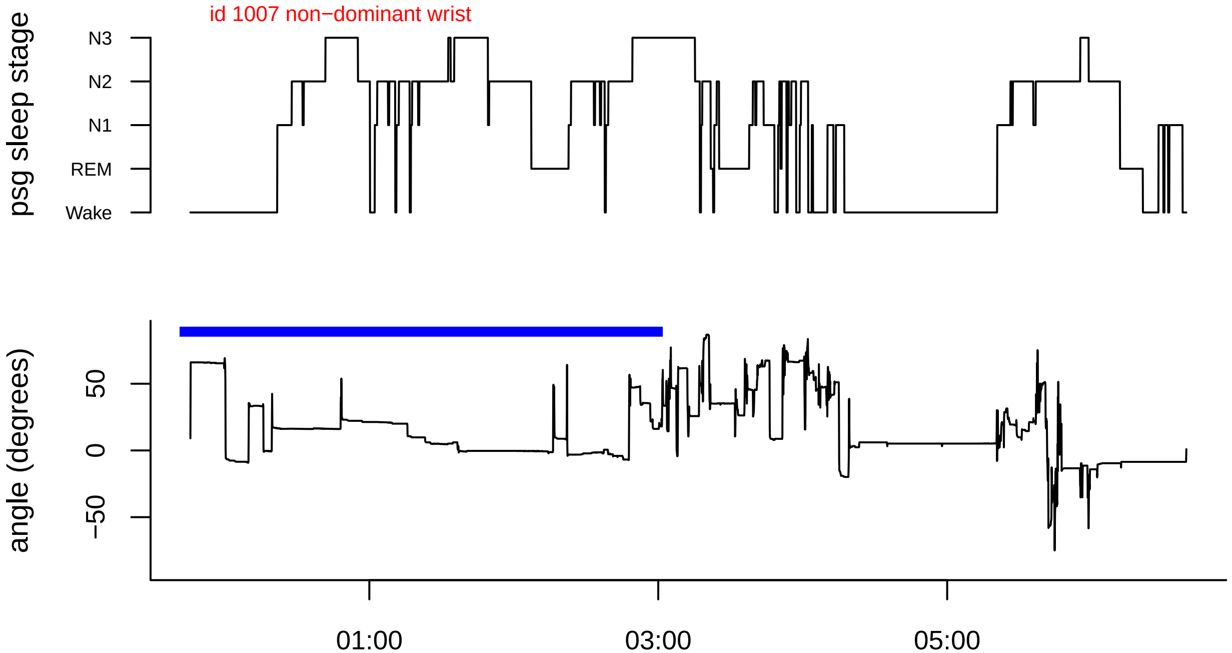

id 1007 non−dominant wrist
psg sleep stage
N3
N2
N1
REM
Wake
angle (degrees)
50
0
−50
01:00
03:00
05:00

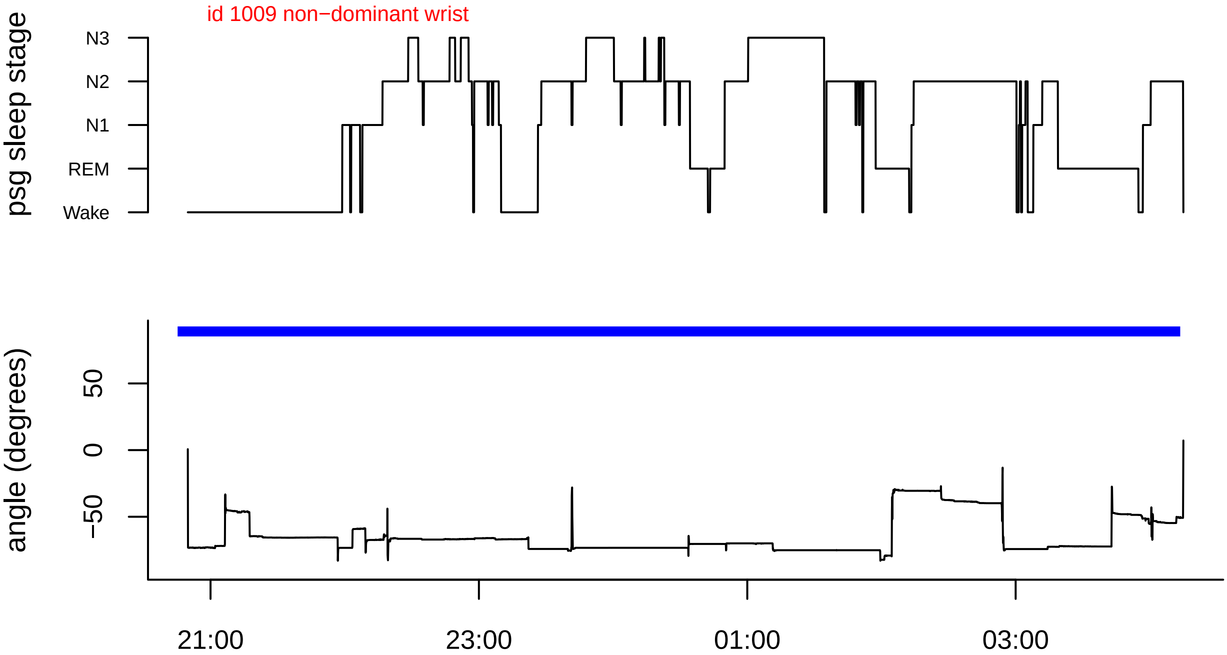

id 1009 non−dominant wrist
psg sleep stage
N3
N2
N1
REM
Wake
angle (degrees)
50
0
−50
21:00
23:00
01:00
03:00

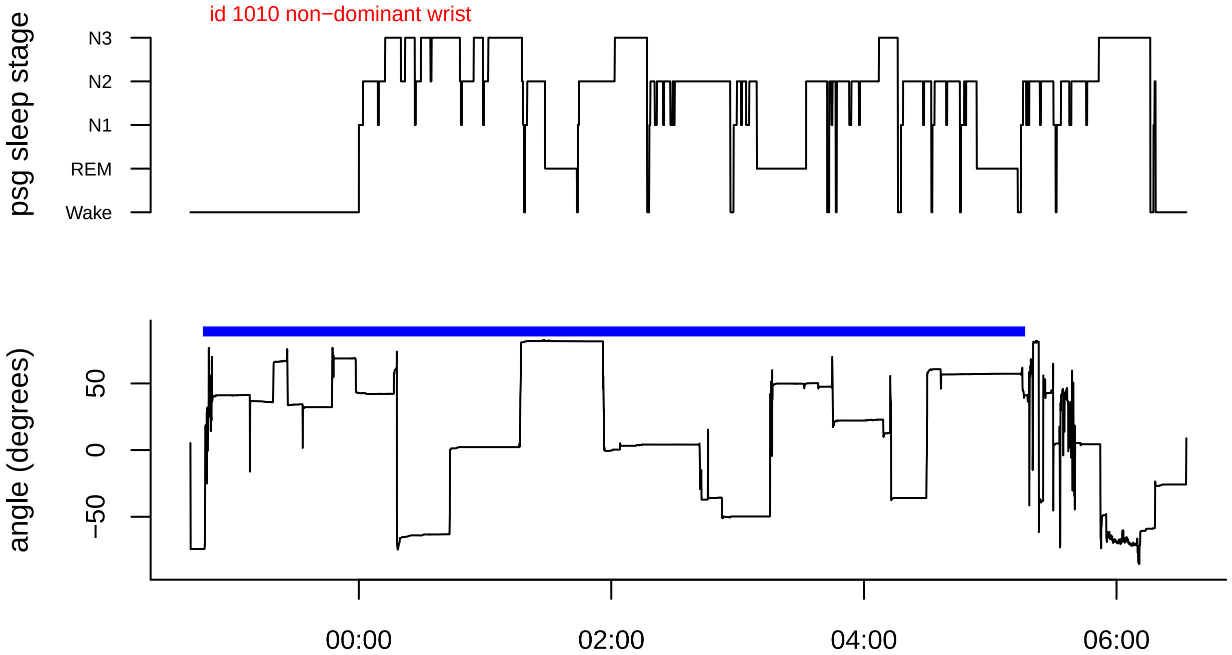

id 1010 non−dominant wrist
psg sleep stage
N3
N2
N1
REM
Wake
angle (degrees)
50
0
−50
00:00
02:00
04:00
06:00

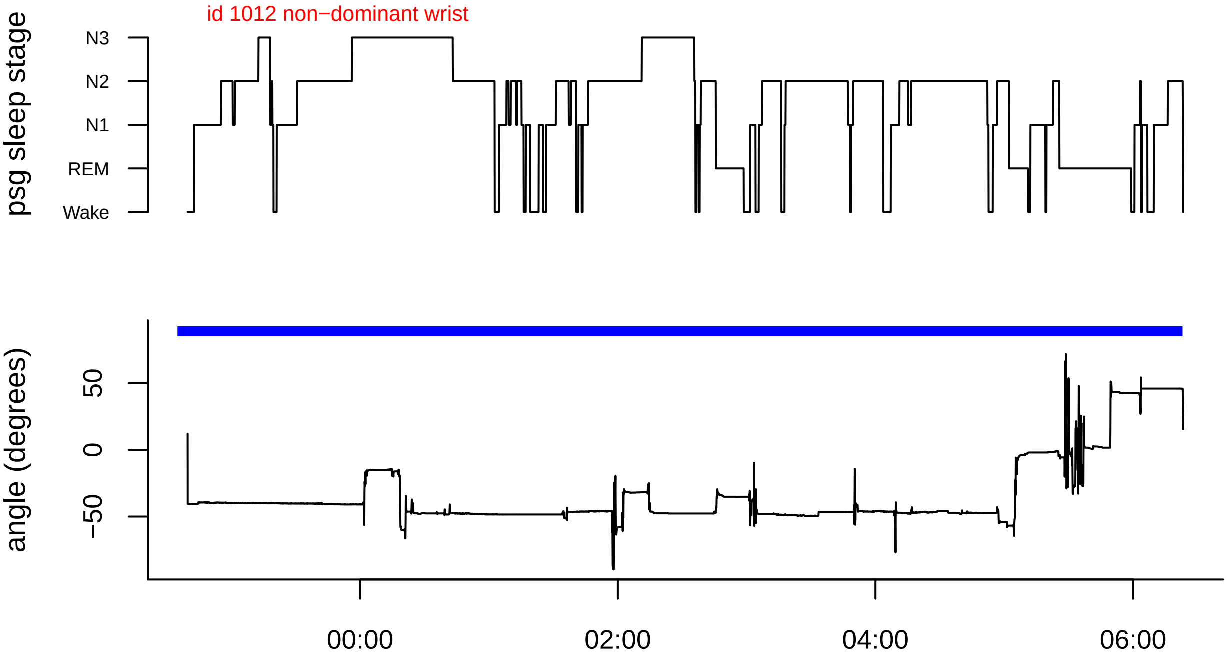

id 1012 non−dominant wrist
psg sleep stage
N3
N2
N1
REM
Wake
angle (degrees)
50
0
−50
00:00
02:00
04:00
06:00

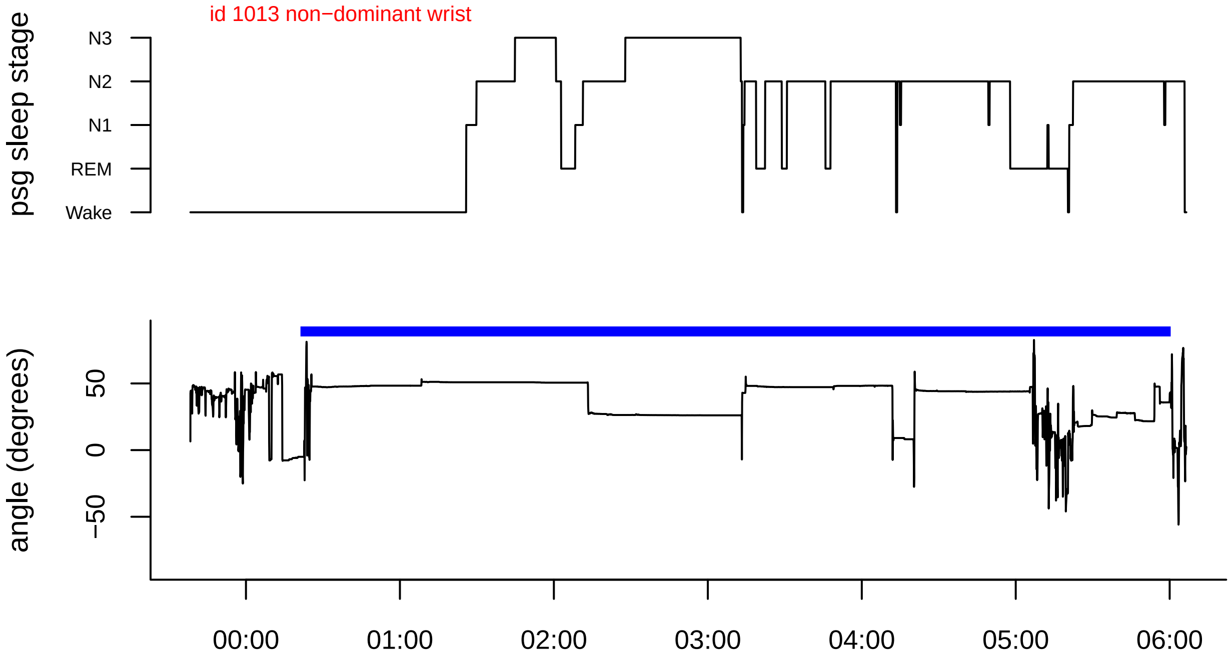
id 1013 non−dominant wrist
psg sleep stage
N3
N2
N1
REM
Wake
angle (degrees)
50
0
−50
00:00
01:00
02:00
03:00
04:00
05:00
06:00

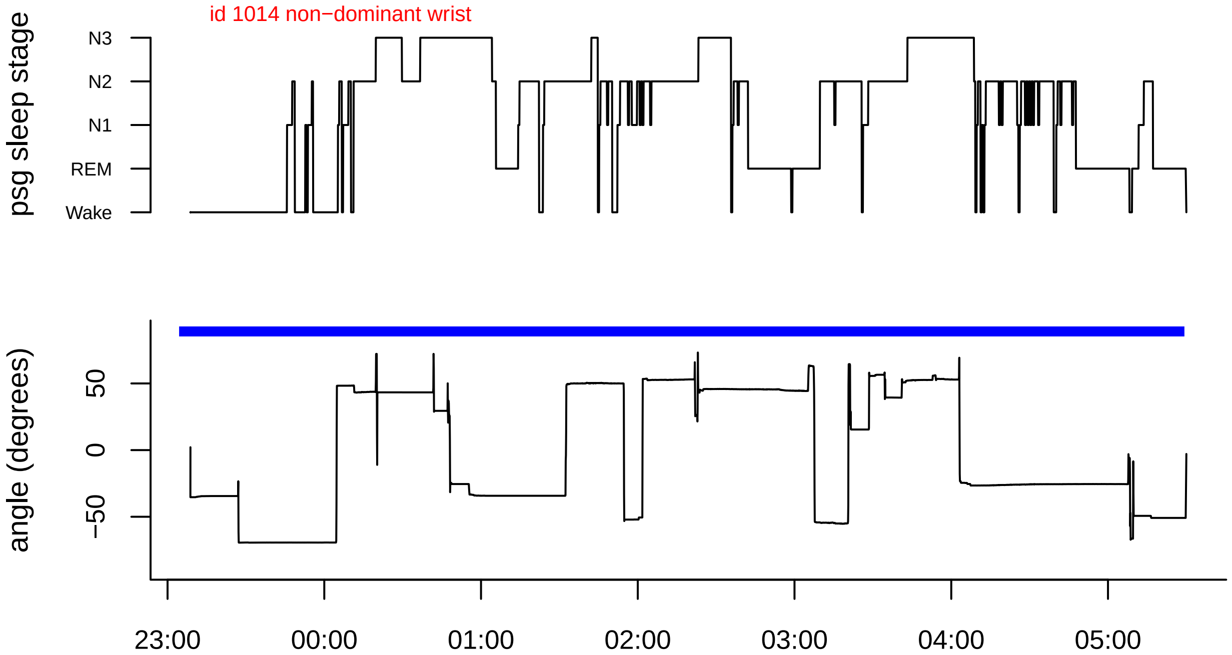

id 1014 non−dominant wrist
psg sleep stage
N3
N2
N1
REM
Wake
angle (degrees)
50
0
−50
23:00
00:00
01:00
02:00
03:00
04:00
05:00

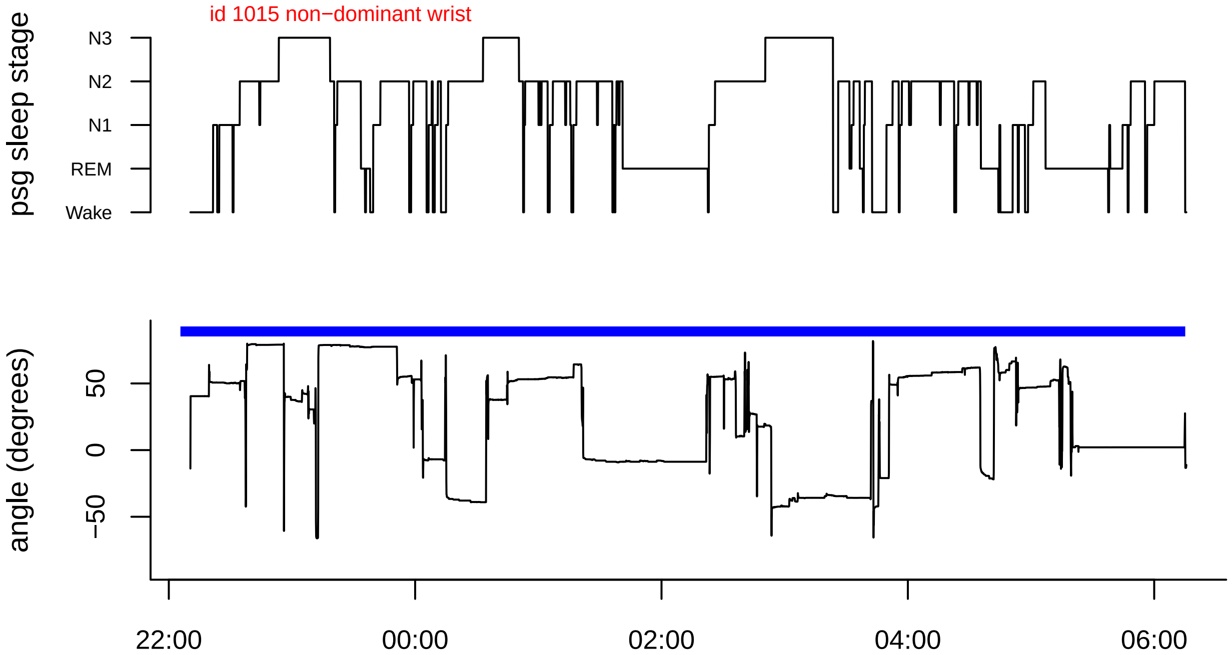
id 1015 non−dominant wrist
psg sleep stage
N3
N2
N1
REM
Wake
angle (degrees)
50
0
−50
22:00
00:00
02:00
04:00
06:00

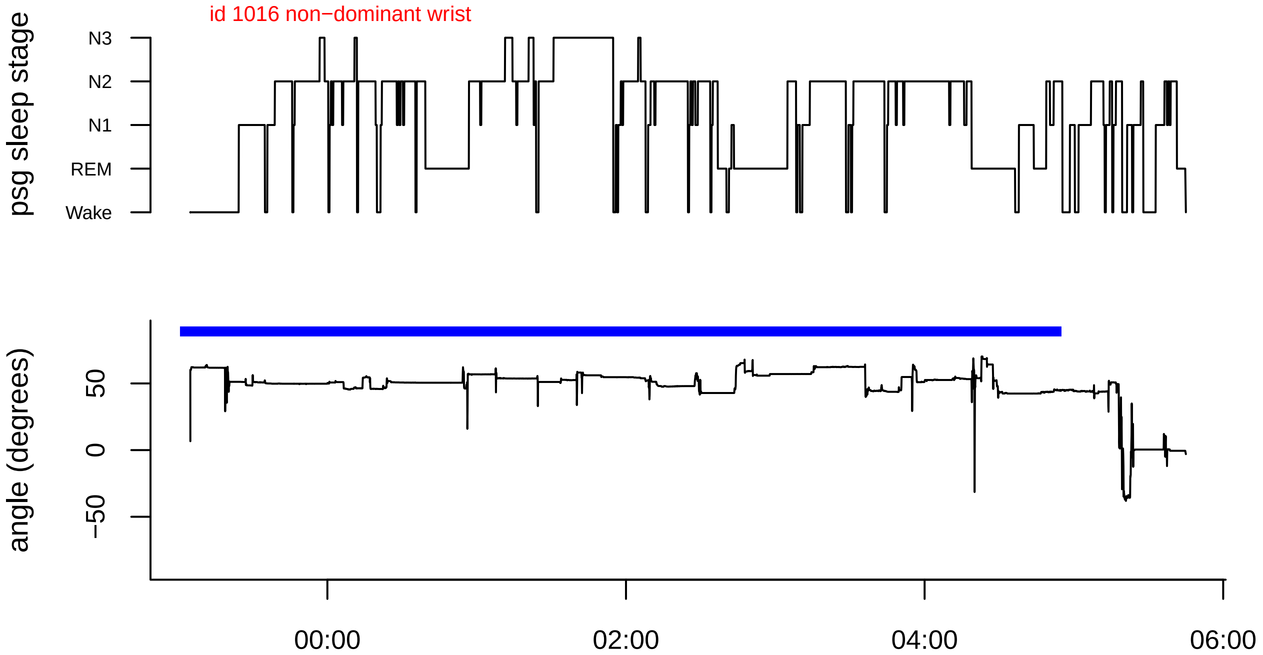
id 1016 non−dominant wrist
psg sleep stage
N3
N2
N1
REM
Wake
angle (degrees)
50
0
−50
00:00
02:00
04:00
06:00

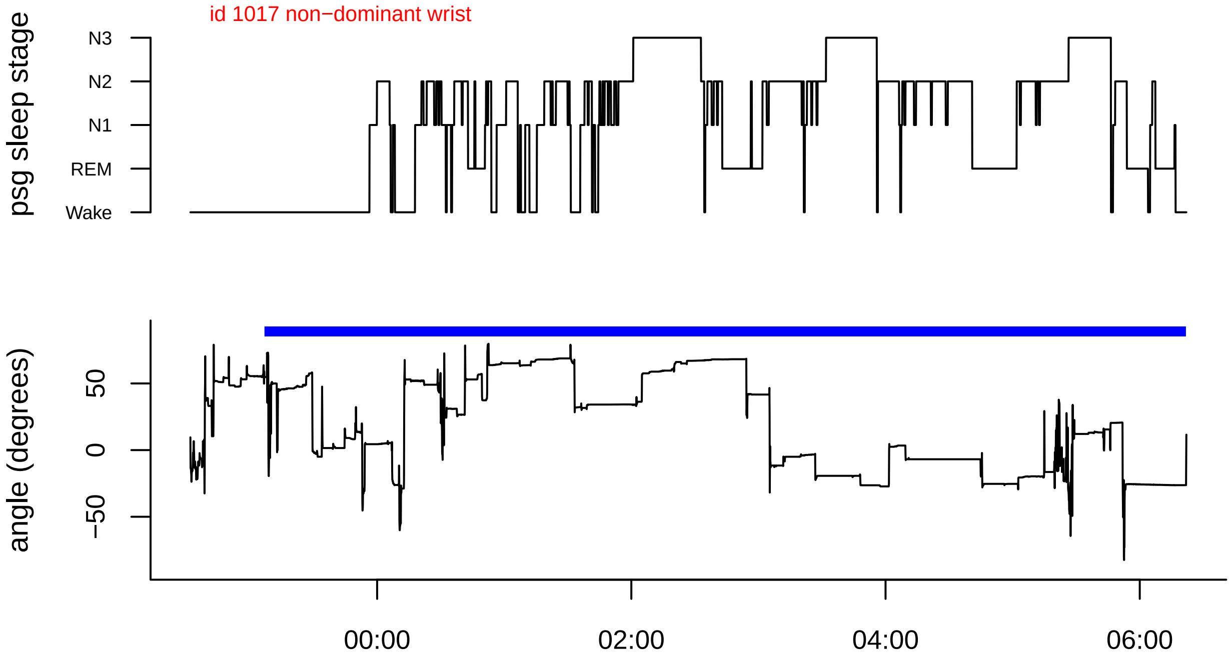

id 1017 non−dominant wrist
psg sleep stage
N3
N2
N1
REM
Wake
angle (degrees)
50
0
−50
00:00
02:00
04:00
06:00

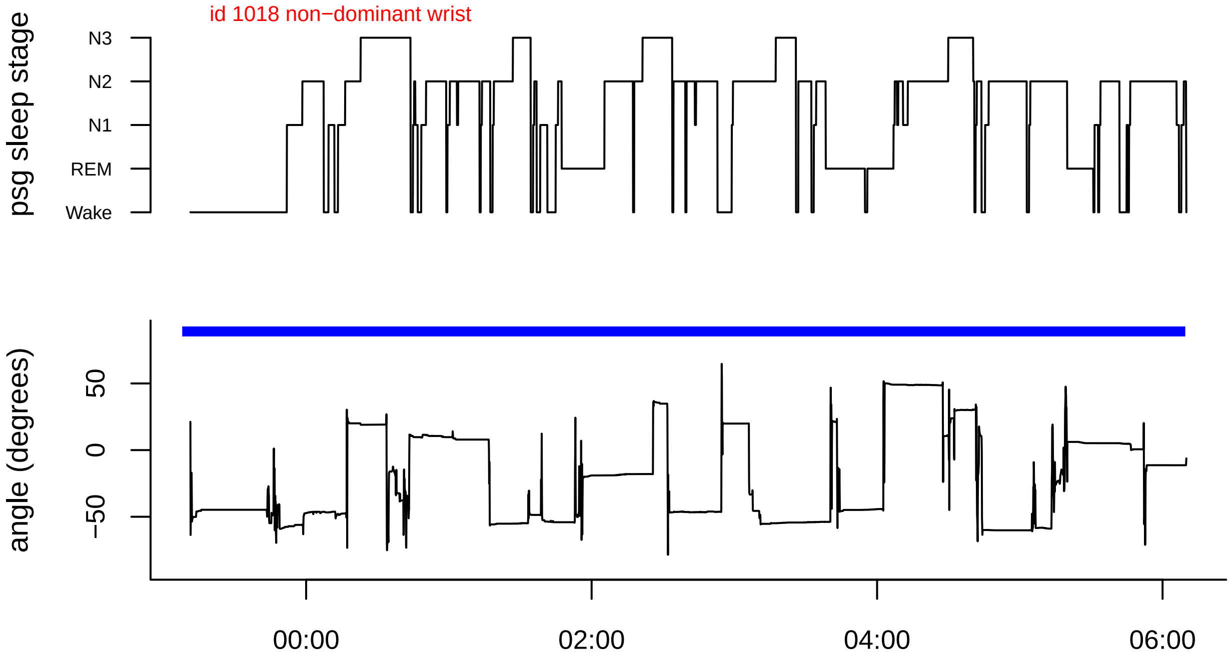
id 1018 non−dominant wrist
psg sleep stage
N3
N2
N1
REM
Wake
angle (degrees)
50
0
−50
00:00
02:00
04:00
06:00

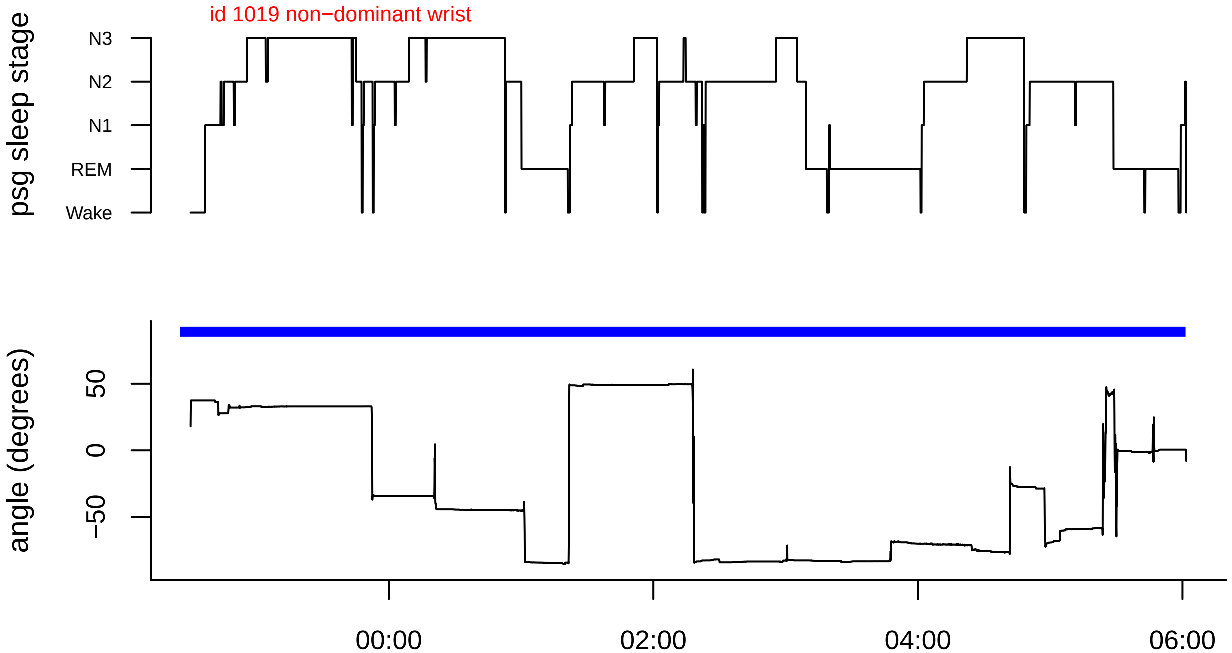

id 1019 non−dominant wrist
psg sleep stage
N3
N2
N1
REM
Wake
angle (degrees)
50
0
−50
00:00
02:00
04:00
06:00

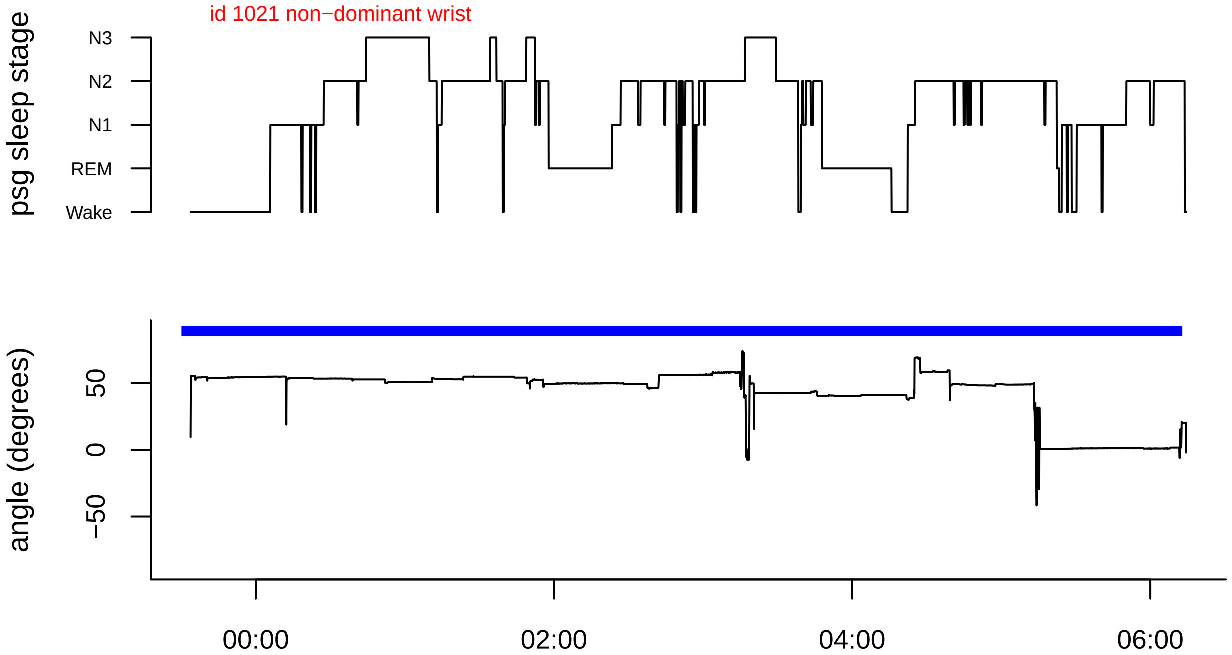

id 1021 non−dominant wrist
psg sleep stage
N3
N2
N1
REM
Wake
angle (degrees)
50
0
−50
00:00
02:00
04:00
06:00

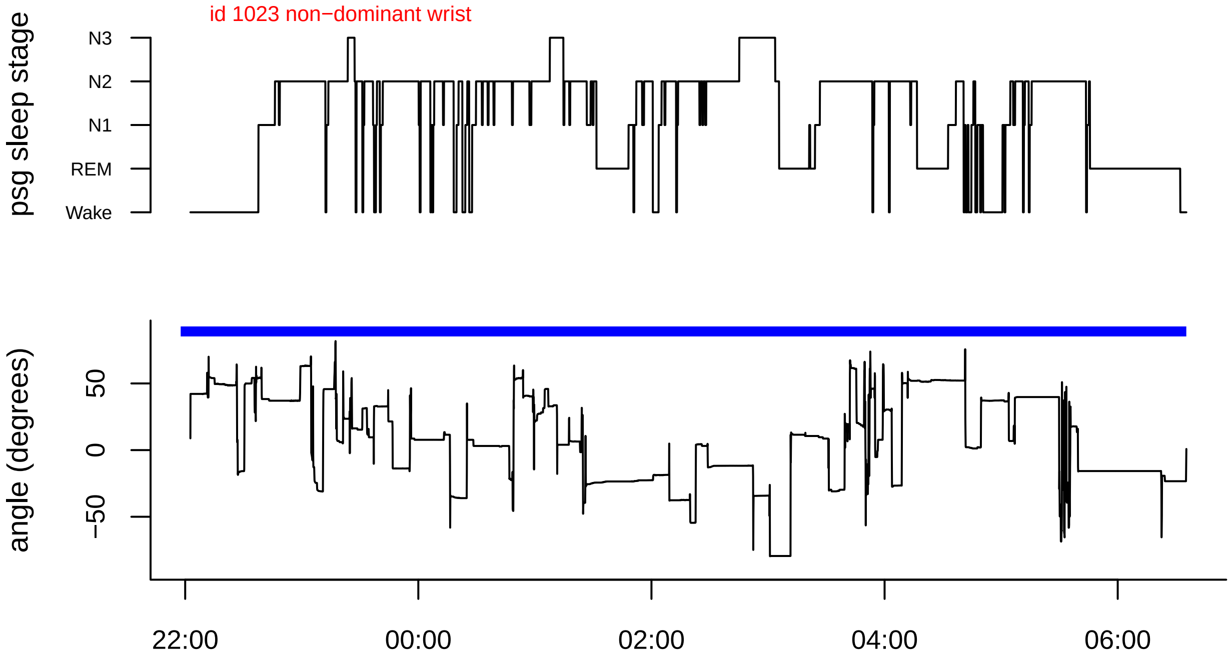

id 1023 non−dominant wrist
psg sleep stage
N3
N2
N1
REM
Wake
angle (degrees)
50
0
−50
22:00
00:00
02:00
04:00
06:00

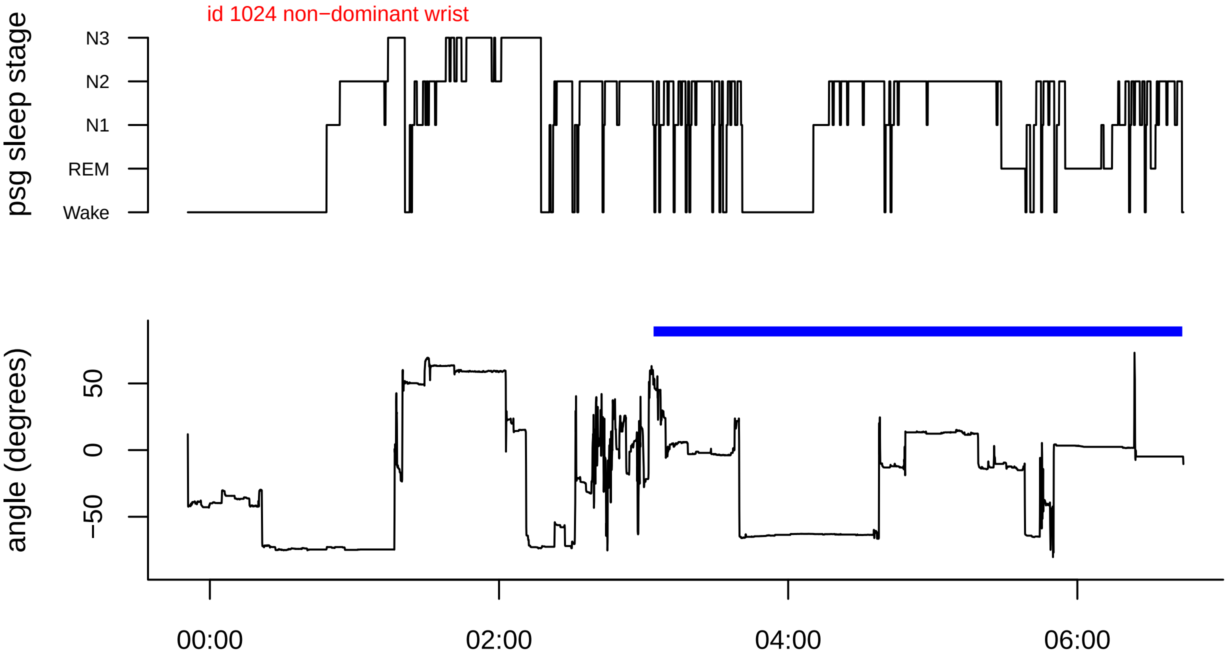
id 1024 non−dominant wrist
psg sleep stage
N3
N2
N1
REM
Wake
angle (degrees)
50
0
−50
00:00
02:00
04:00
06:00

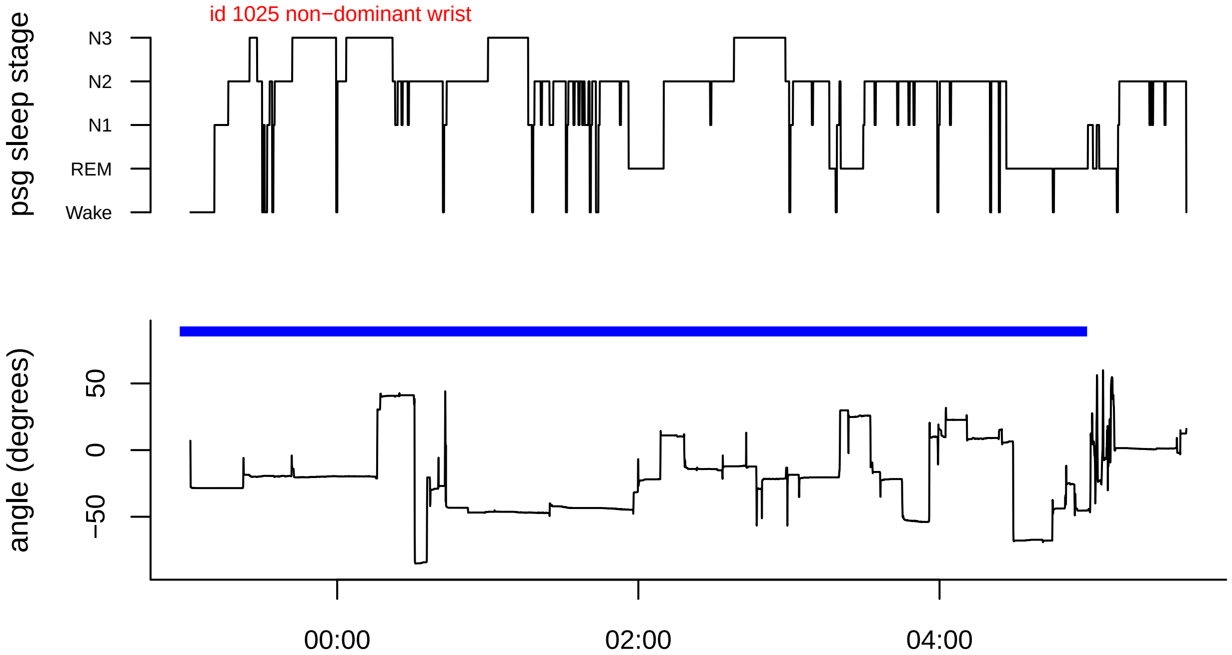

id 1025 non−dominant wrist
psg sleep stage
N3
N2
N1
REM
Wake
angle (degrees)
50
0
−50
00:00
02:00
04:00

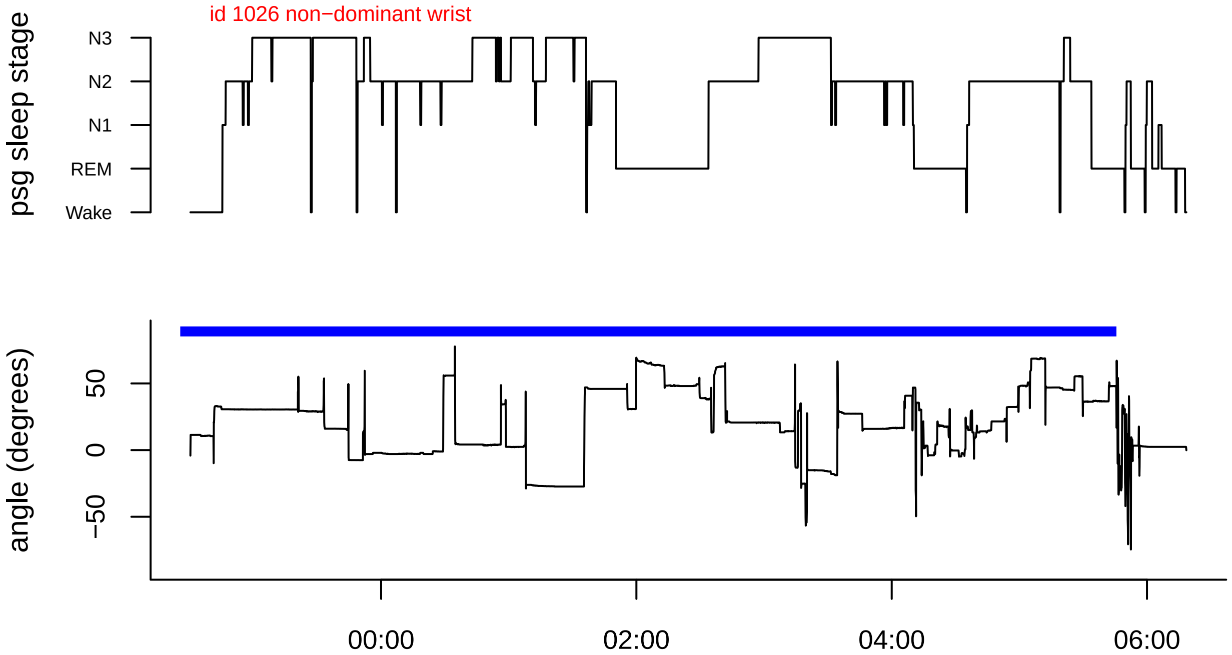
id 1026 non−dominant wrist
psg sleep stage
N3
N2
N1
REM
Wake
angle (degrees)
50
0
−50
00:00
02:00
04:00
06:00

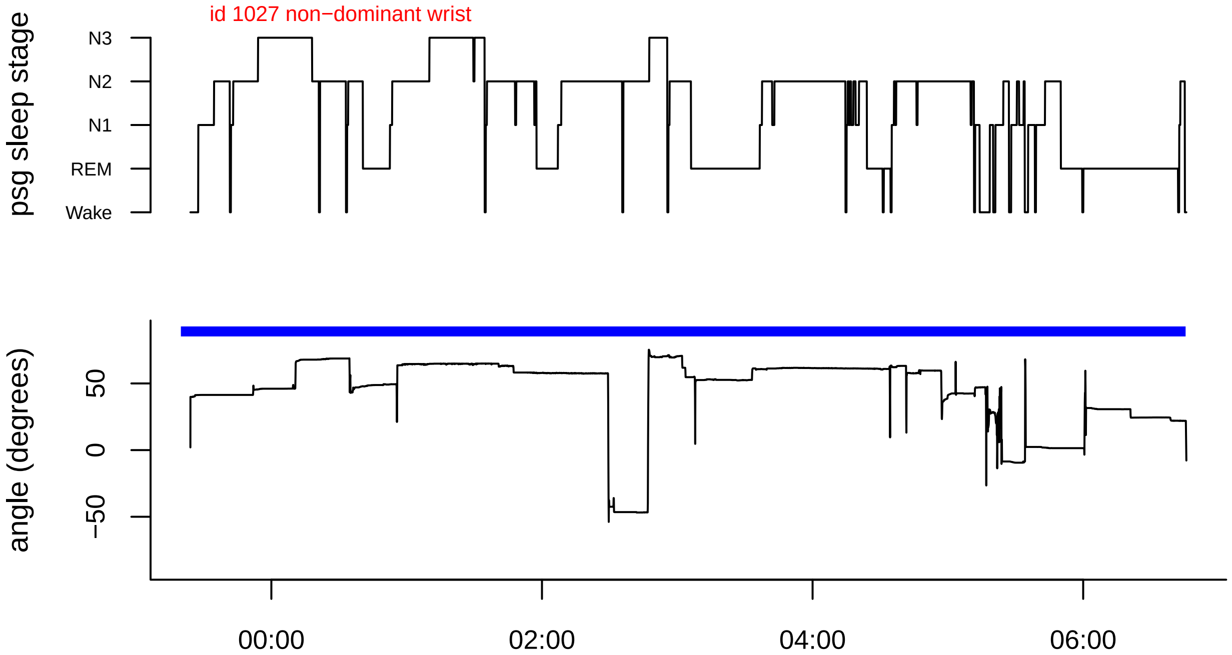

id 1027 non−dominant wrist
psg sleep stage
N3
N2
N1
REM
Wake
angle (degrees)
50
0
−50
00:00
02:00
04:00
06:00

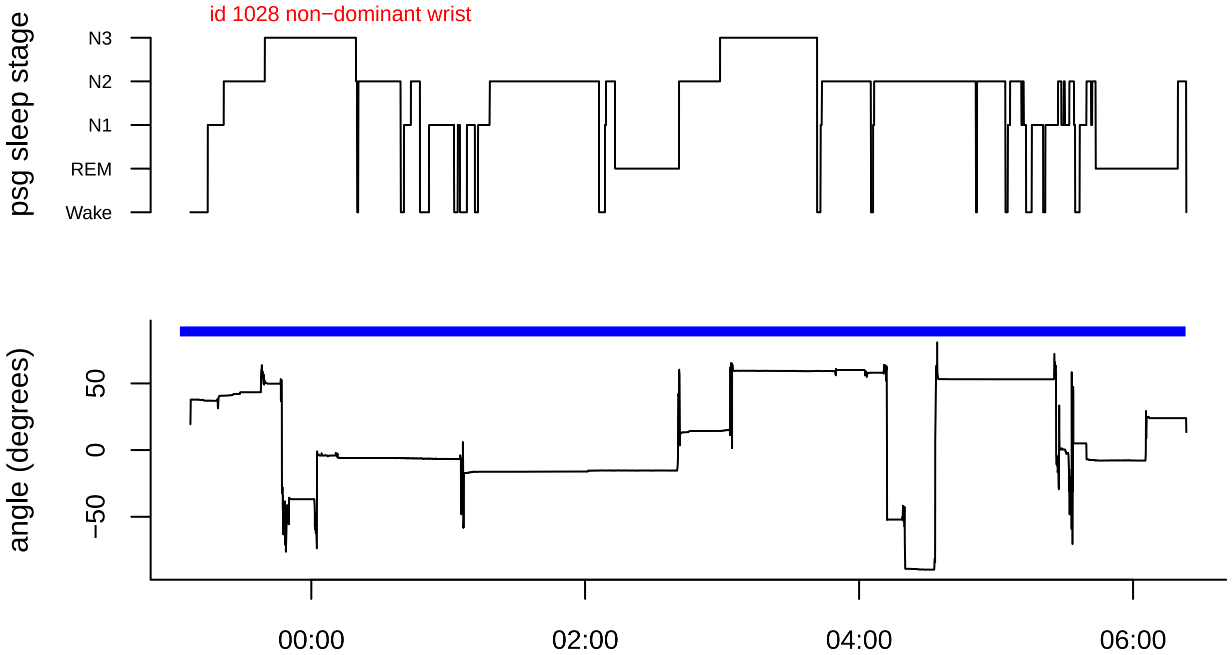

id 1028 non−dominant wrist
psg sleep stage
N3
N2
N1
REM
Wake
angle (degrees)
50
0
−50
00:00
02:00
04:00
06:00

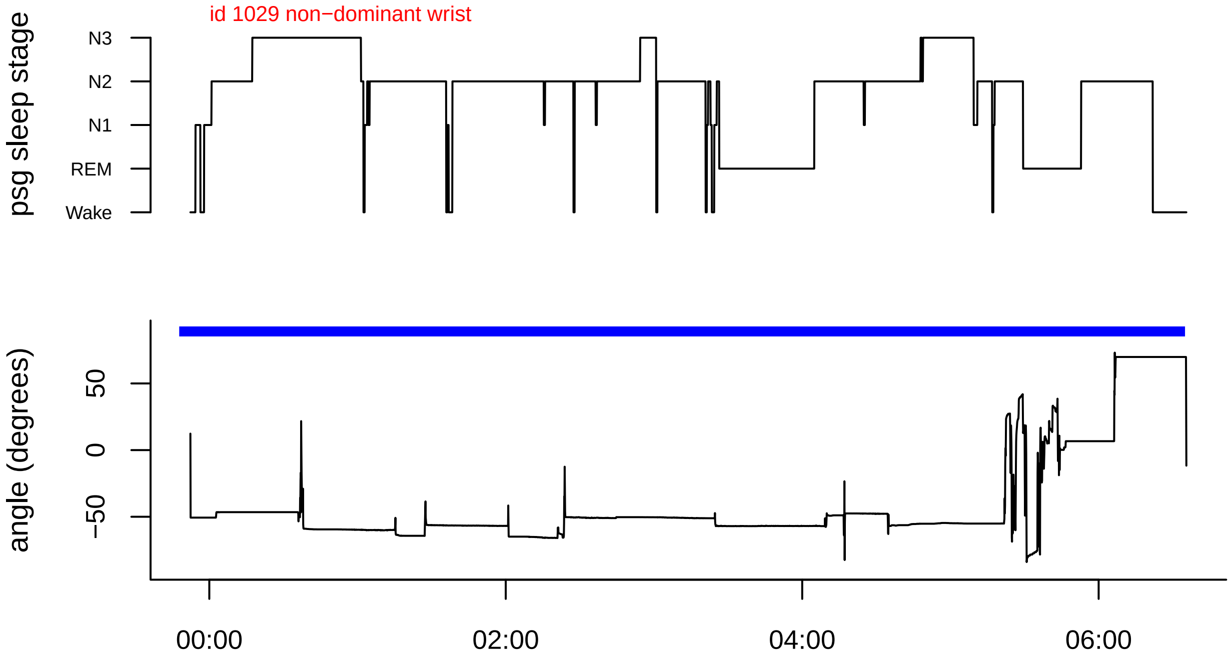

id 1029 non−dominant wrist
psg sleep stage
N3
N2
N1
REM
Wake
angle (degrees)
50
0
−50
00:00
02:00
04:00
06:00

3. Sensitivity analysis

Methods

In this supplement we present the findings from our sensitivity analysis on the HDCZA algorithm parameter configuration. To evaluate the performance of a parameter configuration we used mean absolute error (MAE) of the onset and waking up time, because it is a single number that captures both bias and variance in error. The sensitivity analysis was run on the four main parameters in the algorithm, being the 10th percentile (step 6), the factor 15 (step 6), the 30 minutes (step 7), and the 60 minutes (step 8). Considering the large search space, any choice of alternative parameters would seem arbitrary. In machine learning a random search is considered an acceptable way to optimize parameter configurations if there is little knowledge about the shape of the search space. Therefore, we created thirty random parameter configurations sampled uniformly from plausible value ranges that include the existing values. The search space we explored is:

- Percentile: uniform distribution between 5th and 15th percentile.
- Factor: uniform distribution between factor 10 and 20.
- Short time block: uniform distribution between 15 minutes and 45 minutes.
- Long time block: uniform distribution between 30 and 90 minutes.

The sensitivity analysis was replicated in each of the four full datasets: Sleep diary (Whitehall II Study), sleep clinic PSG left wrist (Newcastle), sleep clinic PSG right wrist (Newcastle), and the healthy good sleepers PSG (Pennsylvanian).

Results

The default algorithm configuration ranked 5th, 10th, 13th, and 16th in respectively the study with sleep diary, -sleep clinic PSG left wrist, sleep clinic PSG right wrist, and healthy good sleepers PSG, see Figures S1-S4. The average and standard deviation of the MAE estimates across the four study conditions are shown in Figure S5. For parameter configurations #8, one of the few configurations that outperformed the default configuration for the HDCZA algorithm we replicated the tables as represented in the main manuscript, see table S2-S5. Parameter configuration #8 entails: percentile = 0.12, threshold = 12; short block =31 minutes, and long block = 73 minutes.

Discussion/Conclusion

The sensitivty analysis indicates that improvement of the configuration within specific study conditions is possible. However, across the four study conditions the current default parameter configuration provides a relatively low (good) average and variation (robust) in MAE compared with other parameter configurations. The best configuration for the sleep clinic PSG study rigth wrist (#20 in Figure S3) is the one-but worst configuration when compared with sleep diaries (#20 in Figure S1), demonstrating the risk of overfitting. When inspecting one of the few parameter configurations (#8) that outperforms the default configuration based on average MAE in figure S5 we observed a few things: The configuration #8 provides similar associations with sleep diary (tables S2 and S3) and polysomnography in healthy good sleepers (table S5), but MAE are lower compared polysomnography in sleep clinic patients (table S4).

Further, it can be observed that the C-statistic, the accuracy and the sensitivity have wider inter quartile ranges in the sleep clinic data (compare table S4 with table 4 in manuscript), indicating that better average MAE can come at the cost of less robust performance on other performance metrics.

Figure S1: **MAE of the HDCZA algorithm against sleep diary per parameter configuration *(default configuration encircled with red)***

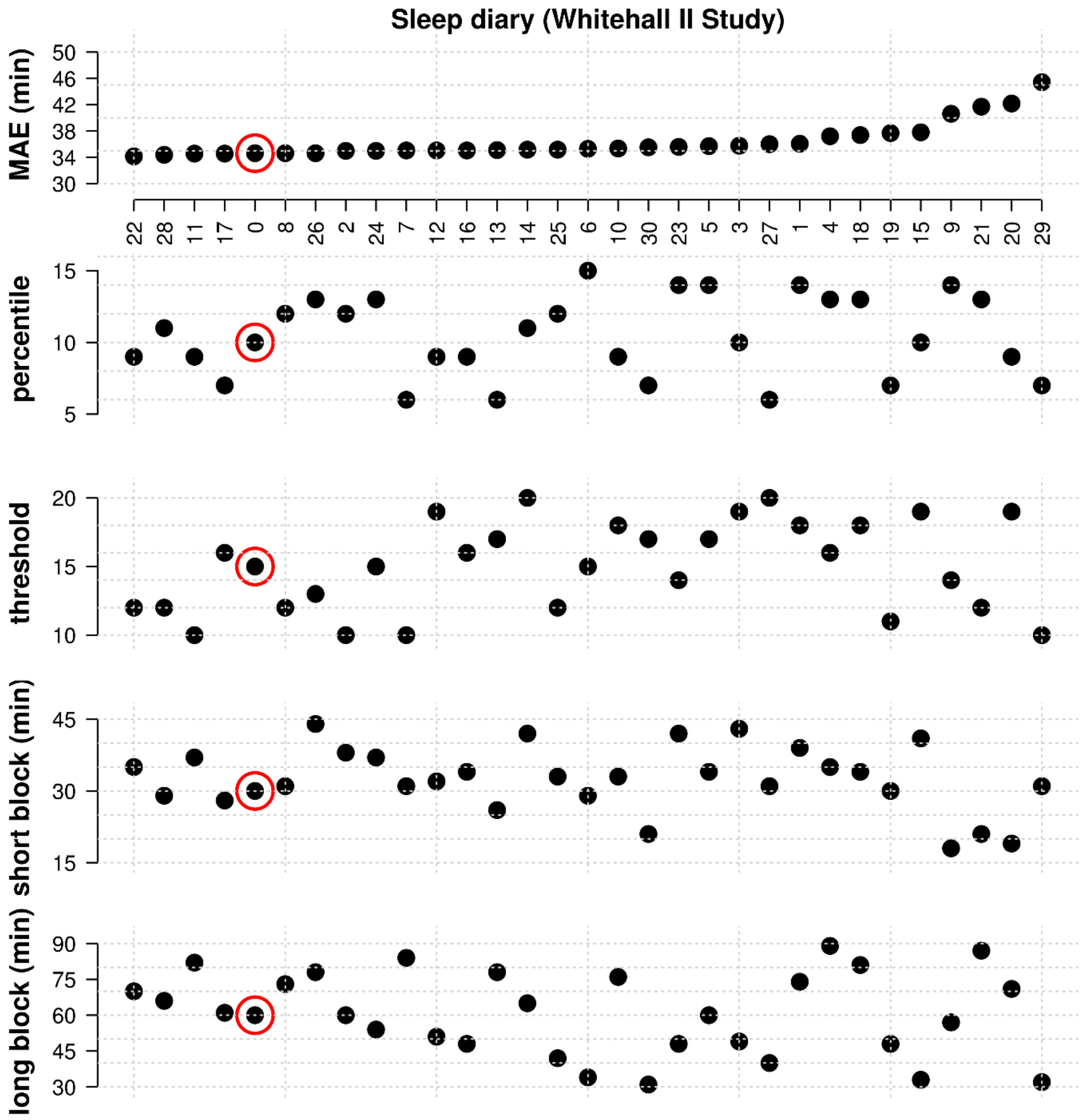

The figure represents MAE of HDCZA algorithm against sleep diary (Whitehall II Study) based on 30 random parameter settings sorted based on their MAE. The parameter setting numbered 0 corresponds to the default configuration (encircled with red).

Figure S2: **MAE of the HDCZA algorithm against sleep clinic PSG left wrist per parameter configuration *(default configuration encircled with red)***

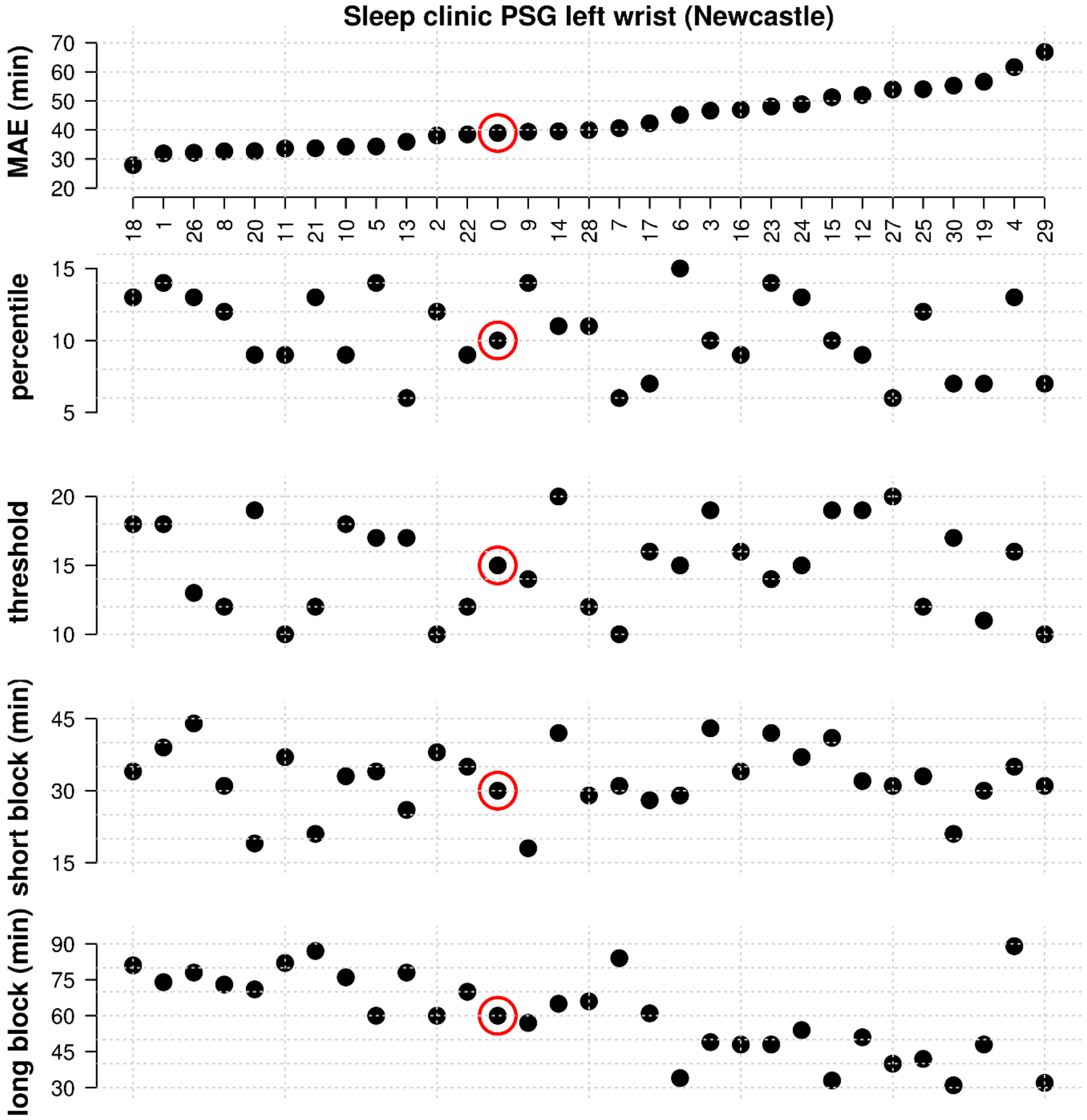

The figure represents MAE of HDCZA algorithm against sleep clinic PSG left wrist (Newcastle) based on 30 random parameter settings sorted based on their MAE. The parameter setting numbered 0 corresponds to the default configuration (encircled with red).

Figure S3: **MAE of the HDCZA algorithm against sleep clinic PSG right wrist per parameter configuration** ***(default configuration encircled with red)***

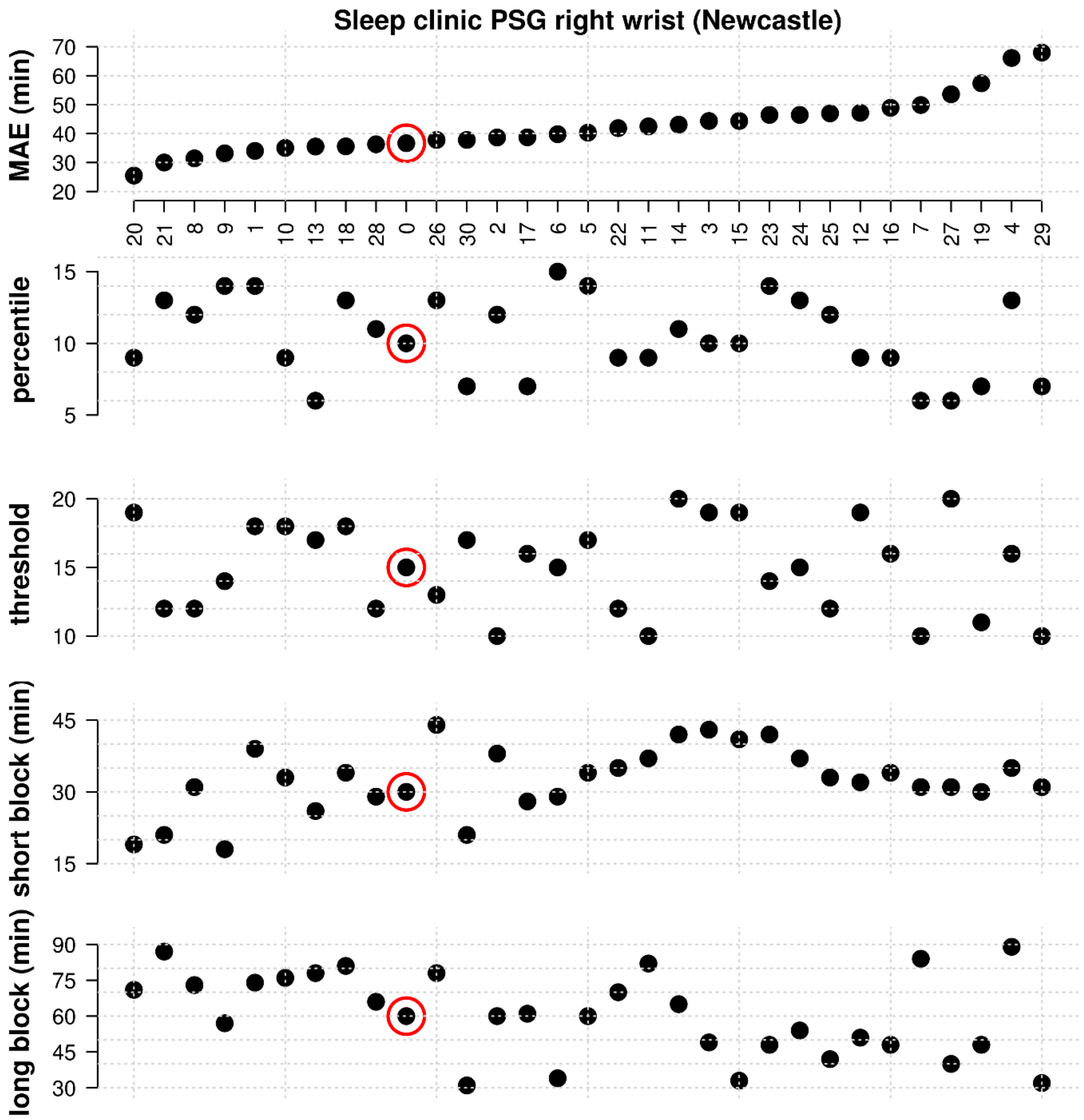

Figure S4: **MAE of the HDCZA algorithm against PSG non-dominant wrist in healthy good sleepers per parameter configuration *(default configuration encircled with red)***

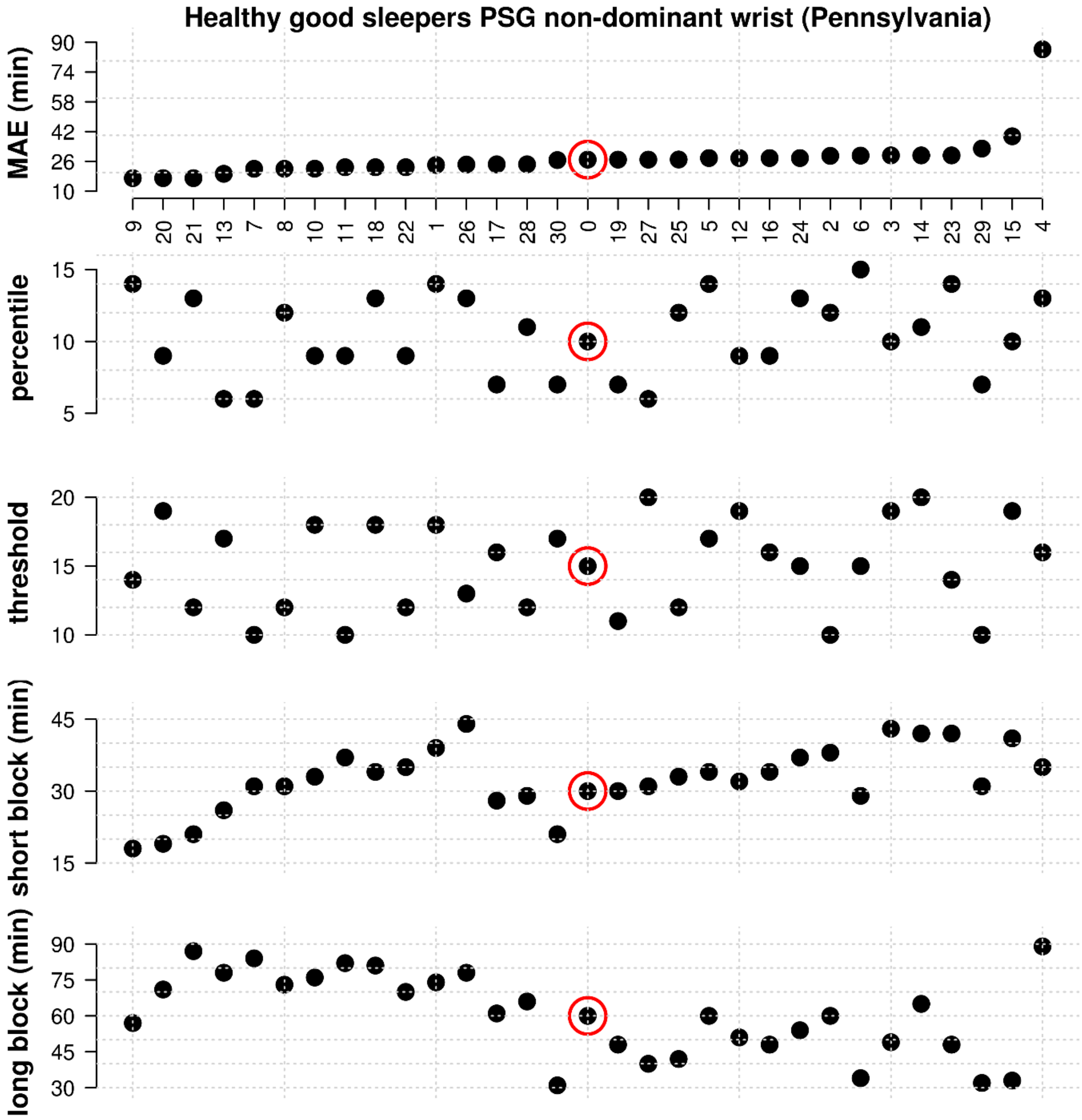

Figure S5: Mean and standard deviation of MAE across studies **per parameter configuration *(default configuration encircled with red)***

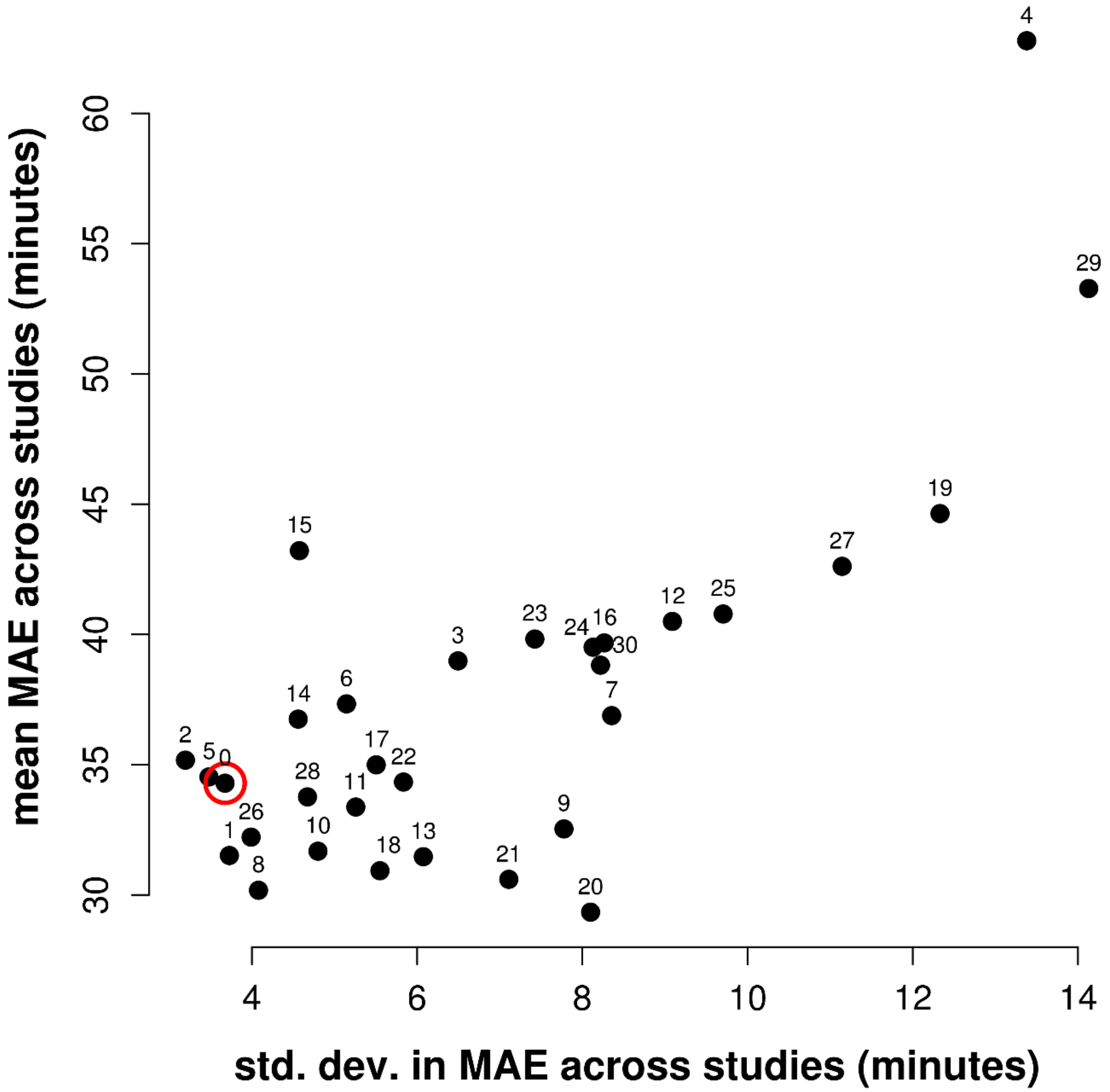

Table S2: Sleep parameter differences (minutes) between estimates from sleep diary and two accelerometer-based methods (N=25,640 nights) for HDCZA parameter configuration #8.

Sleep parameters	HDCZA		
**Method**	**Sleep onset time**	**Waking time**	**SPT-window duration**
**Y-intercept (SE)**	-15 (0.9) **	-0.7 (0.7) P=0.33	14.3 (1) **
**Betas (SE)**			
Women	4.8 (1.1) **	-3.2 (0.9) **	-8 (1.3) **
Ten years of age †	3.6 (0.8) **	-2.1 (0.7) *	-5.7 (1) **
Five BMI index points ‡	0.7 (0.5) P=0.17	-1.3 (0.5) *	-2 (0.7) *
Weekend	3.1 (1) *	2.4 (0.8) *	-0.8 (1.2) P=0.5
Winter	1 (0.9) P=0.26	-0.7 (0.8) P=0.35	-1.7 (1.1) P=0.12
Within individual residual SD	25	19.8	24.3
Between individual residual SD	67	56.8	83.7
AIC	81863	73017	91991

[Degrees of freedom=25,576; † relative to mean age of 69.1 years; ‡ relative to mean BMI of 26.4 kg / m^2; SE: Standard Error; SD: Standard Deviation; AIC = Akaike information coefficient, * P < .005, ** P < .0005]

Table S3: Correlation, mean absolute error, and concordance between sleep diary and accelerometer estimates (N=3,752) for HDCZA parameter configuration #8

Parameter	Metric	HDCZA		
		**Value**	**t; DF**	**P**
sleep onset time	Correlation in timing	0.78 (95% CI: 0.76 - 0.79)	76; 3750	**
	MAE (min)	40.5		
waking time	Correlation in timing	0.81 (95% CI: 0.8 - 0.83)	86; 3750	**
	MAE (min)	29.4		
SPT-window	Correlation in duration	0.54 (95% CI: 0.52 - 0.56)	39; 3750	**
	MAE (min)	39.7		
	c-statistic	0.95 (IQR: 0.94 - 0.98)	-	-

[DF: Degrees of freedom; MAE: mean absolute error; min: minutes; ** P < 0.0005]

Table S4: Comparison algorithm with polysomnography in sleep clinic patients (Newcastle study) for HDCZA parameter configuration #8

Parameters	Metric	Left wrist (N=28)			Right wrist (N=27)		
		Value	t; DF	P	Value	t; DF	P
Sleep onset	Difference (min)	-15 (95% CI: -33 - 3)	-1.68; 27	0.10	-10 (95% CI: -31 - 10)	-1.03; 26	0.31
	MAE (min)	27.8			33.0		
Sleep wake	Difference (min)	-29 (95% CI: -62 - 4)	-1.78; 27	0.09	-6 (95% CI: -35 - 22)	-0.45; 26	0.66
	MAE (min)	37.5			29.8		
SPT-window	Difference in duration (min)	-15 (95% CI: -53 - 24)	-0.77; 27	0.45	4 (95% CI: -35 - 42)	0.20; 26	0.84
	MAE (min)	55.7			54.0		
	c-statistic	0.87 (IQR: 0.57-0.94)	-	-	0.87 (IQR: 0. 63-0.95)	-	-
	c-statistic 24 hour†	0.95 (IQR: 0.59-0.99)	-	-	0.96 (IQR: 0.63-0.99)	-	-
	Accuracy (%)	89 (IQR: 28-98)	-	-	90 (IQR: 57-98)	-	-
	Accuracy 24 hour† (%)	95 (IQR: 73-99)	-	-	96 (IQR: 74-99)	-	-
Sleep within SPT	Difference in duration (min)	37 (95% CI: 13 - 61)	3.16; 27	*	48 (95% CI: 19 - 77)	3.37; 26	*
	Sensitivity (%)	94 (IQR: 15-100)	-	-	94 (IQR: 0-100)	-	-
Sleep efficiency within SPT	Difference (percent point)	7.2 (95% CI:3.5 - 11)	3.95; 27	*	8.7 (95% CI:3.0 - 14.5)	3.14; 26	*
	MAE (percent point)	8.6			10.5		

[* P < .005; MAE: mean absolute error; min: minutes; SPT-window: Sleep period time window; CI: Confidence Interval; DF: degrees of freedom; t: t-statistic; IQR: Inter quartile range; † recording expanded with simulated data of wakefulness to resemble 24 hours]

Table S5: Comparison algorithm with polysomnography in healthy good sleepers (N=22, Pennsylvania) for HDCZA parameter configuration #8

Parameters	Metric	Value	t; DF	P
Sleep onset	Difference (min)	-20 (95% CI: -39 - -2)	-2.31; 21	0.03
	MAE (min)	32.9	-	-
Sleep wake	Difference (min)	-8 (95% CI: -17 - 1)	-1.75; 21	0.10
	MAE (min)	11.1	-	-
SPT-window	Difference in duration (min)	12 (95% CI: -7 - 30)	1.29; 21	0.21
	MAE in duration (min)	31.2	-	-
	c-statistic	0.84 (IQR: 0.80-0.90)	-	-
	c-statistic 24 hour†	0.97 (IQR: 0.96-0.99)	-	-
	Accuracy (%)	91 (IQR: 89-97)	-	-
	Accuracy 24 hour† (%)	97 (IQR: 96-99)	-	-
Sleep within SPT	Difference in duration (min)	0 (95% CI: -19 - 19)	-0.01; 21	0.99
	Sensitivity (%)	96 (IQR: 98-100)	-	-
Sleep efficiency within SPT	Difference (percent point)	-2.5 (95% CI: -4.7 - -0.2)	-2.35; 21	0.04
	MAE (min)	4.2	-	-

[MAE: mean absolute error; min: minutes; SPT-window: Sleep period time window; CI: Confidence Interval; DF: degrees of freedom; t: t-statistic; IQR: Inter quartile range; † recording expanded with simulated data of wakefulness to resemble 24 hours]
